# Pharmacomodulation of the Antimalarial Plasmodione: Synthesis of Biaryl- and *N*-Arylalkylamine Analogues, Antimalarial Activities and Physicochemical Properties

**DOI:** 10.3390/molecules22010161

**Published:** 2017-01-19

**Authors:** Karène Urgin, Mouhamad Jida, Katharina Ehrhardt, Tobias Müller, Michael Lanzer, Louis Maes, Mourad Elhabiri, Elisabeth Davioud-Charvet

**Affiliations:** 1 UMR 7509 CNRS-Université de Strasbourg, Bioorganic and Medicinal Chemistry Team, European School of Chemistry, Polymers and Materials (ECPM), 25, rue Becquerel, F-67087 Strasbourg, France; urginkarene@gmail.com (K.U.); mhmd_jida@yahoo.com (M.J.); ehrhardt.ka@gmail.com or k.ehrhardt@ibmc-cnrs.unistra.fr (K.E.); tobias_mueller79@web.de (T.M.); elhabiri@unistra.fr (M.E.); 2 Zentrum für Infektiologie, Parasitologie, Universität Heidelberg, Im Neuenheimer Feld 324, 69120 Heidelberg, Germany; michael.lanzer@med.uni-heidelberg.de; 3 Biochemie-Zentrum der Universität Heidelberg, Im Neuenheimer Feld 328, D-69120 Heidelberg, Germany; 4 Laboratory of Microbiology, Parasitology and Hygiene (LMPH), Faculty of Pharmaceutical, Biomedical and Veterinary Sciences, University of Antwerp, Universiteitsplein 1, B-2610 Antwerp, Belgium; louis.maes@uantwerpen.be

**Keywords:** antimalarial, *N*-arylalkylamines, atovaquone, biaryls, menadione, 1,4-naphthoquinone, plasmodione, redox-cycling, reductive amination, Suzuki-Miyaura coupling

## Abstract

With the aim of increasing the structural diversity on the early antimalarial drug plasmodione, an efficient and versatile procedure to prepare a series of biaryl- and *N*-arylalkylamines as plasmodione analogues is described. Using the naturally occurring and commercially available menadione as starting material, a 2-step sequence using a Kochi-Anderson reaction and subsequent Pd-catalyzed Suzuki-Miyaura coupling was developed to prepare three representative biphenyl derivatives in good yields for antimalarial evaluation. In addition, synthetic methodologies to afford 3-benzylmenadione derivatives bearing a terminal -*N*(Me)_2_ or -*N*(Et)_2_ in different positions (*ortho*, *meta* and *para)* on the aryl ring of the benzylic chain of plasmodione were investigated through reductive amination was used as the optimal route to prepare these protonable *N*-arylalkylamine privileged scaffolds. The antimalarial activities were evaluated and discussed in light of their physicochemical properties. Among the newly synthesized compounds, the *para*-position of the substituent remains the most favourable position on the benzyl chain and the carbamate -*N*HBoc was found active both in vitro (42 nM versus 29 nM for plasmodione) and in vivo in *Plasmodium berghei*-infected mice. The measured acido-basic features of these new molecules support the cytosol-food vacuole shuttling properties of non-protonable plasmodione derivatives essential for redox-cycling. These findings may be useful in antimalarial drug optimization.

## 1. Introduction

Malaria is caused by four species of protozoan parasites of the genus *Plasmodium* in tropical and subtropical areas and is curable if treated promptly and adequately. The most dangerous and harmful species, *P. falciparum* is responsible for malignant malaria with complications such as cerebral malaria or severe anaemia, and is responsible for significant mortality and morbidity including the death of approximately 0.6 million people worldwide by year, mostly African children younger than 5 years [1]. Artemisinin-based Combination Therapy (ACT) is currently the first-line therapy for malaria worldwide; however, a decrease in artemisinin sensitivity, mainly in Southeast Asia, has recently been reported, thus underlining the need for alternative artemisinin-based combinations with novel agents as drug partners [2]. New antimalarials must exhibit pleiotropic mechanisms-of-action or target new metabolic pathways that counterbalance parasite resistance to current drugs. Additionally, there is a continuous demand for new drugs that are not only affordable, safe, available and effective but also easy to synthesize. Our approach is to target the thiol based redox-network of *P. falciparum*.

**Figure 1:**
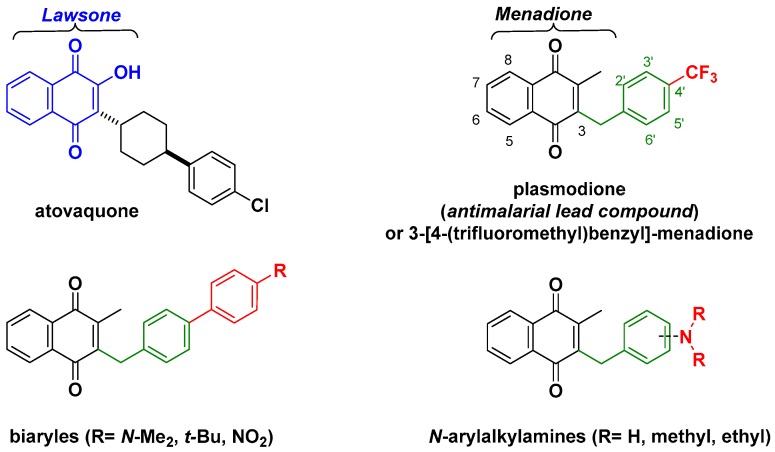
Structurally related antimalarial atovaquone (**left**) and the lead plasmodione (**right**) and envisaged synthetic 3-benzylmenadione/plasmodione derivatives (2nd line): biaryl- (**left**) and *N*-arylalkylamine (**right**) analogues of plasmodione.

Recently, we synthesized a series of 3-benzylmenadione derivatives [3,4], which were identified as potent antimalarial redox-active agents. Among these, the 3-[4-(trifluoromethyl)benzyl]-menadione (called plasmodione, Figure 1) showed the most promising ‘early lead’ features for drug optimization [5,6]. Mechanistic studies revealed that plasmodione may enter a cascade of redox reactions for drug bioactivation: in its oxidized state, the menadione derivative was proposed to be reduced by the NADPH-dependent glutathione reductases of the *Plasmodium*-parasitized erythrocytes; in its reduced state, the hydronaphthoquinone was shown to transfer 1e^−^ to methemoglobin (Fe^III^) (metHb), the major nutrient of the parasite [3,4,7]. By redox-cycling NADPH and hemoglobin, it was postulated that the increased oxidative stress leads to the death of the parasite, but only in parasitized erythrocytes.

In a previous study, it was found that the mitochondrial electron transport (mETC) chain of *P. falciparum* blood stages is dispensable for the antimalarial activities of plasmodione and methylene blue in contrast to the 2-hydroxy-naphthoquinone derivative atovaquone [5]. The latter drug is given as a highly efficacious fixed-dose combination (atovaquone-proguanil marketed as Malarone^®^) both for prophylaxis and treatment of multidrug-resistant *falciparum* malaria. Atovaquone inhibits complex III of the mETC system, inhibits the cell respiratory mechanism and depolarizes the mitochondrial membrane potential [8]. However, the major function of the mETC in *P. falciparum* blood stage cultures is to regenerate ubiquinone as the electron acceptor of mitochondrial dihydroorotate dehydrogenase (DHODH) which catalyzes a key step in pyrimidine biosynthesis [9]. Atovaquone has been shown to lose its antimalarial activity in the presence of a yeast gene encoding an alternative cytosolic DHODH (yDHODH) that does not require the mETC as electron acceptor [10]. Transgenic *P. falciparum* strains with or without yDHODH were established providing an excellent tool for the analysis of potential inhibitors of the mETC. The previous study [5] evaluated redox-active 1,4-naphthoquinones, including plasmodione and methylene blue, and were shown to retain potency against the transgenic *P. falciparum* strain expressing yDHODH, which had lost sensitivity to atovaquone. Furthermore, chemical modification on the menadione moiety of plasmodione derived analogues were functionalized with the atovaquone chain [11], but these hybrid molecules were found inactive against the atovaquone-sensitive Dd2 strain of *P. falciparum* because 1,4-naphthoquinones plasmodione and atovaquone do not share the same mode-of-action and redox potential values. In addition, atovaquone and plasmodione were clearly shown to result in different morphological changes during the intraerythrocytic parasitic cycle [5]. In agreement with previous reports [12], the morphologies of atovaquone-treated parasites were very similar to the untreated controls. The growth of plasmodione-treated parasites was retarded with a significant increase in abnormal morphologies, predominantly pyknotic ring stages. In contrast to atovaquone, the observations on morphology are in agreement with plasmodione’s pronounced activity against ring stages compared to later stages. Other electrochemical parameters for lawsone versus menadione (2-hydroxy versus 2-methyl-1,4-naphthoquinone, see Figure 1) reported in [11,13] support the distinct redox behaviour and mode-of-action of both 1,4-naphthoquinone antimalarials atovaquone and plasmodione.

The bioavailability and activity/solubility of the plasmodione series is however poor, hindering further drug development. Hence, investigations on its pharmaco-modulation are necessary to determine whether the 4-trifluoromethyl group can be replaced or whether the substituent on the aryl ring can be moved without sacrificing the antimalarial activity. Based on the literature on the activity of numerous.

4- or 8-aminoquinolines or phenothiazines, and as a part of our medicinal chemistry program on 1,4-naphthoquinone scaffolds, the present study investigated the antimalarial activity of several plasmodione derivatives in which the 4′-CF_3_ group was replaced either by a substituted aryl group or by a *N*-dialkylamino group. Modifications on the menadione core previously provided various plasmodione analogues with varying antiplasmodial activity [14]. In the present study the substitution at the benzylic chain of plasmodione was explored, in particular by using Pd-catalyzed reactions to prepare biphenyl analogues. Biaryls and their aryl-heteroaryl homologues represent an important class of organic compounds found in natural products that were shown to be useful in various applications, ranging from medicinal chemistry [15] to advanced materials and supramolecular chemistry [16,17,18]. For example, the diphenylmethyl chain of the potent substituted quinolone CK-2-68 was replaced by a pyridylaryl chain in the initial lead compound SL-2-25 without sacrificing its antimalarial activity [19,20].

**Figure 2:**
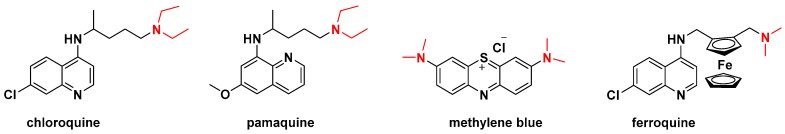
The most important clinically used antimalarial drugs presenting a terminal -*N*(Me)_2_ or -*N*(Et)_2_ group in their side chains.

Herein, biaryl- and *N*-arylalkylamine analogues of plasmodione were synthesized to investigate their antimalarial potential in comparison to plasmodione. For *N*-substitution, we incorporated *N*-dimethyl or *N*-diethyl moieties as found in the approved drugs chloroquine (CQ) [21,22] and related 4- or 8-aminoquinolines (e.g., ferroquine [23,24] or pamaquine [25,26]) and Methylene Blue (MB) [27,28,29] (Figure 2) to learn the structural requirements for antimalarial activity of optimized plasmodione derivatives. We further evaluated the impact of the free amino and *N*-dialkyl substituents on the physicochemical properties of derivatives **3**, **7**, **10**, **11** and **17**–**19** in comparison to those of two key reference drugs MB and CQ since p*K*_a_ values are important for antimalarials that mainly interact with Fe^III^ species.

MB, the first synthetic antimalarial dye discovered by Paul Ehrlich in 1891, has recently been rediscovered for its promising activity in drug combinations for the control of transmission of *P. falciparum* malaria in endemic regions [30] and against *Plasmodium vivax* [31]. MB is reduced by various NADPH-dependent disulfide reductases, including glutathione reductases from the cytosols of *Plasmodium*-infected erythrocytes [32]. Malarial parasites normally digest the host hemoglobin in the metHb form in a specialized acidic compartment, called food vacuole, mainly at the intraerythrocytic trophozoite stage. The blue dye is known to cycle between the oxidized form and its electron–reduced leucomethylene blue (LMB) form that is involved in intricate redox processes, in particular conversion of metHb to oxyhemoglobin (oxyHb) [29]. As oxyHb is not digested by the parasitic proteases, MB has been proposed to exert its antimalarial action by depriving the parasite from its major nutrient metHb. With its phenothiazine structure, MB displays redox cycling activity able to generate bound (hemoglobin or heme) Fe^II^ from Fe^III^ species, likely responsible for its potent activity in vivo. Upon reduction and different protonation states, MB has the ability to change its structure and display different lipophilicities [29]. Due to its very low p*K*_a_ (p*K*_a_ = 0), MB does not accumulate in acidic vesicles but may shuttle in and out the cell compartments depending on its redox state. The final result of MB-catalyzed redox-cycling is an arrest of parasite development [5].

In contrast, the broadly used CQ is a diprotic weak base (p*K*_a_ = 8.4 and 10.6), which accumulates in the food vacuole and binds to Fe^III^-heme leading to a stable 1:1 π-π complex (*K*_D_ ~ 2–15 µM at pH 7.5) [33]. Intraerythrocytic parasites detoxify the exogenous deleterious Fe^III^-heme by-product (i.e., predominantly existing as a dimeric π-π heme complex) of hemoglobin digestion through biomineralization to an insoluble and much less active crystal, named hemozoin. In the presence of CQ, the final result is the elevation of toxic free heme and inhibition of hemozoin formation. Free heme and heme CQ complexes are therefore thought to kill parasites by inducing severe oxidative stress [34], which successively leads to peroxidation of the parasite membrane lipids, DNA damage, protein oxidation and ultimately death of the parasite.

## 2. Results

### 2.1. Chemistry

Previously, we reported a new methodology for the preparation of 3-benzylmenadione derivatives [14] and polysubstituted 6-methylquinoline-5,8-dione scaffolds [35] (i.e., 5- and 8-aza-menadione analogues) by the Kochi-Anderson reaction. The silver-catalyzed coupling reaction represents an easy access and efficient method to synthesize certain 3-benzyl-substituted 1,4-naphthoquinones derivatives starting from readily commercially available carboxylic acids [36]. This reaction has also been applied in the synthesis of naturally occurring quinones [37,38] or in the synthesis of 2- and 3-substituted menadiones as subversive substrates of trypanothione reductase and lipoamide dehydrogenase from *Trypanosoma cruzi* [39,40] or of glutathione reductases of *Plasmodium*-parasitized erythrocytes [41].

**Scheme 1:**
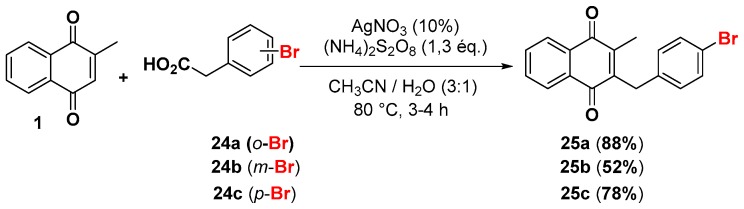
Synthesis of 3-[4-(bromol)benzyl]-menadione intermediate **25** (**a**–**c**) via a Kochi-Anderson reaction.

In continuation of our structure-activity relationships (SAR) studies on the plasmodione series (Scheme 1), the plasmodione bromine derivatives **25a**–**c** were prepared from menadione in moderate to excellent yields (52%–88%) by the described silver-catalyzed decarboxylation reaction between menadione **1** and the corresponding bromophenylacetic acid derivatives **24a**–**c**.

Transition metal catalyzed cross-coupling reactions have revolutionized organic synthesis. Many highly efficient and mild methods have been developed over the last two decades allowing rapid and versatile access to complex molecular structures [42,43,44,45,46,47]. In recent years, the Suzuki-Miyaura reaction in particular has become one of the most important cross-coupling reactions, allowing not only the formation of C(sp^2^)-C(sp^2^) and C(sp^2^)-C(sp^3^) but also C(sp^3^)-C(sp^3^) bonds [44,45,46].

**Scheme 2:**
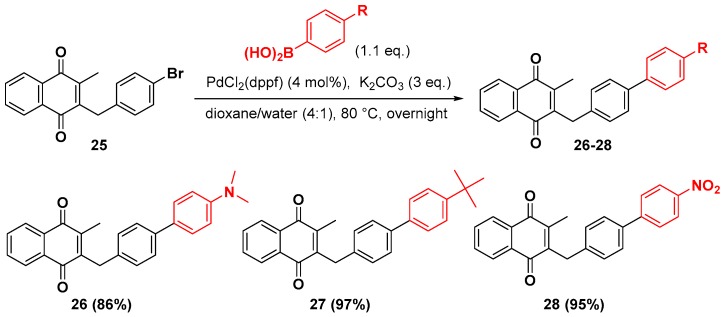
Synthesis of biphenyl derivatives of menadione by Suzuki-coupling reaction.

Subsequently, the biaryl menadione derivatives **26**–**29** (Scheme 2) were successfully synthesized in excellent yields (86%–97%) via a Suzuki-Miyaura coupling reaction. The reaction was performed in dioxane/water (*v/v* = 4/1) at 80 °C overnight under an atmosphere of argon. Aryl bromide **25c** was coupled with the corresponding *para*-substituted phenylboronic acid in the presence of K_2_CO_3_ as base and 4 mol % PdCl_2_(dppf) as catalyst. K_2_CO_3_ was chosen as a mild base because of the low stability of benzylmenadione derivative **25c** under basic conditions due to the acidic proton of the CH_3_ group. Using these reaction conditions, no side product was observed compared to the treatment with stronger bases leading to a rapid decomposition of the starting menadione derivatives. The expected Suzuki-products **26**–**28** were isolated in excellent yield. This methodology is straightforward and provides easy access to structurally diverse 1,4-naphthoquinones with a biphenyl motif, allowing to study the influence of a second phenyl ring in plasmodione structure on its antimalarial activity.

**Scheme 3:**
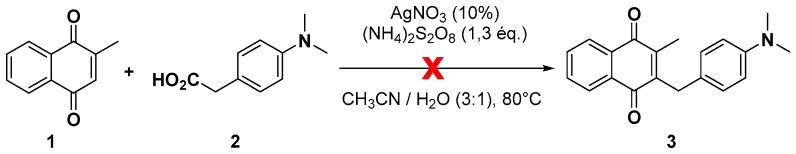
Silver-catalyzed decarboxylation reaction (Kochi-Anderson).

Regarding the 2nd series of *N*-arylalkylamines, we first prepared the *N*-dialkylamine analogues of 3-benzylmenadiones (Figure 2) using the Kochi-Anderson reaction. In earlier investigations to synthesize compound **3** (Scheme 3), the reported conditions used in the silver-catalyzed decarboxylation reaction of menadione **1** with 4-(dimethylamino)phenylacetic acid **2** had proven to be inappropriate and not compatible, leading mainly to degradation of the starting materials because the *N*-substituent becomes oxidized in the reaction by Ag(II) leading to the formation of Ag(I) and thereby destroying the competent catalyst Ag(II).

**Scheme 4:**
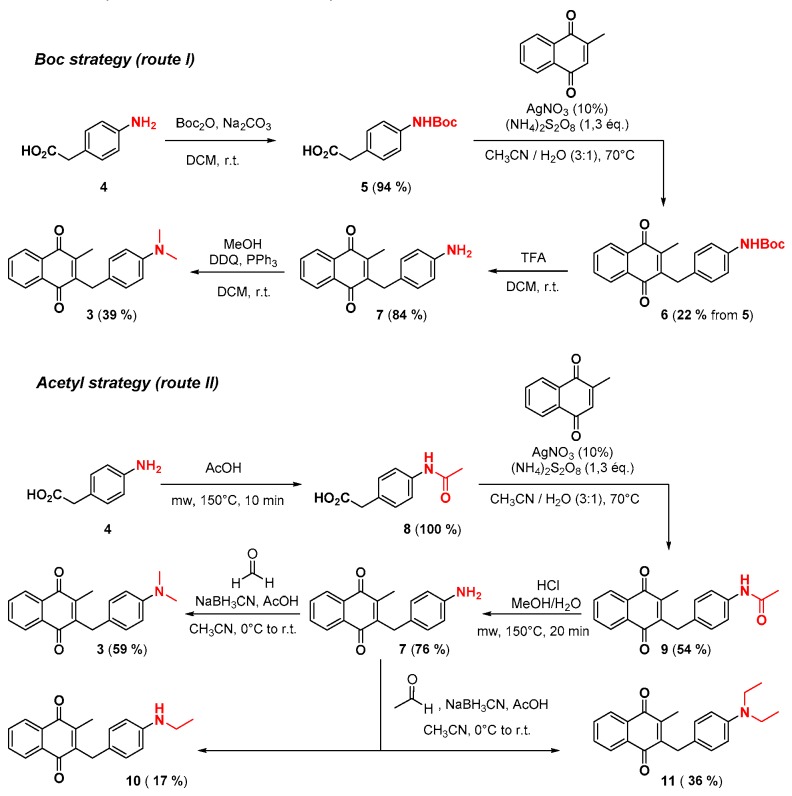
Synthesis of new 3-[4′-*N*-alkylated-benzyl]-menadione derivatives via a reductive amination procedure using the *N*-Boc or -acetyl protection strategies.

It was deemed of interest to explore a new pathway for this purpose. We attempted to achieve the synthesis of *para*-*N*-substituted-3-benzylmenadione derivatives **3** via a *N*-alkylation procedure using two synthetically *N*-protected routes through: (i) the *N*-*tert*-butyloxycarbonyl (*N*-Boc) and (ii) the *N*-acetyl protection strategies (Scheme 4).

Following the *N*-Boc route (i), the commercial 4-aminophenylacetic acid (**4**) was treated with Boc_2_O in the presence of Na_2_CO_3_ as base to give the corresponding *N*-Boc-protected phenylacetic acid **5** in 94% isolated yield (route I, Scheme 4). The 3-benzylmenadione intermediate **6** was synthesized in low yield (22%) by one-step Kochi-Anderson reaction between the phenylacetic acid derivative **5** and menadione (**1**) in the presence of Ag(I) as catalyst and ammonium peroxodisulfate. After removal of the *N*-Boc protecting group in the presence of trifluoroacetic acid (TFA), compound **7** was isolated in 84% yield as the precursor for *N*,*N*-dimethylation of the free amine group in the next step. Finally, the desired product **3** was obtained in 33% yield using Ph_3_P/DDQ [48] in methanol as an efficient reagent system for the immediate and selective *N*-alkylation of aromatic amines. However, to improve the synthesis and overall yields of the desired product **3**, we studied the effect of the *N*-protecting group by replacement of the Boc group by an acetyl group. Indeed, by following the *N*-acetyl route (II), the *N*-acetyl-protected phenylacetic acid **8** was obtained quantitatively by an acetylation reaction between the free-amine **4** and acetic acid under microwave irradiation conditions [49]. Next, the Kochi–Anderson reaction between the phenylacetic acid derivative **8** and menadione (**1**) gave access to the desired compound **9** in good yield (54%). The deprotection reaction of the acetyl group was performed in the presence of hydrochloric acid (HCl) under microwave irradiation conditions, affording the desired compound **7** in 76% yield [50]. As shown in Scheme 4, the expected compound *N*,*N*-dimethyl-3-benzylmenadione **3** was synthesized in good yield (59%) by one-step reductive amination reaction between the intermediate **7** and formaldehyde in the presence of acetic acid and sodium cyanoborohydride as reducing reagent (59% with NaBH_3_CN/AcOH versus 39% with DDQ/PPh_3_) [51]. Use of acetaldehyde instead of formaldehyde under the same reduction reaction conditions (NaBH_3_CN/AcOH) gave access to a separable mixture of *para*-*N*-ethyl-3-benzylmenadione (**10**, 17%) and *para*-*N*,*N*-diethyl-3-benzylmenadione (**11**, 36%) (Scheme 4).

**Scheme 5:**
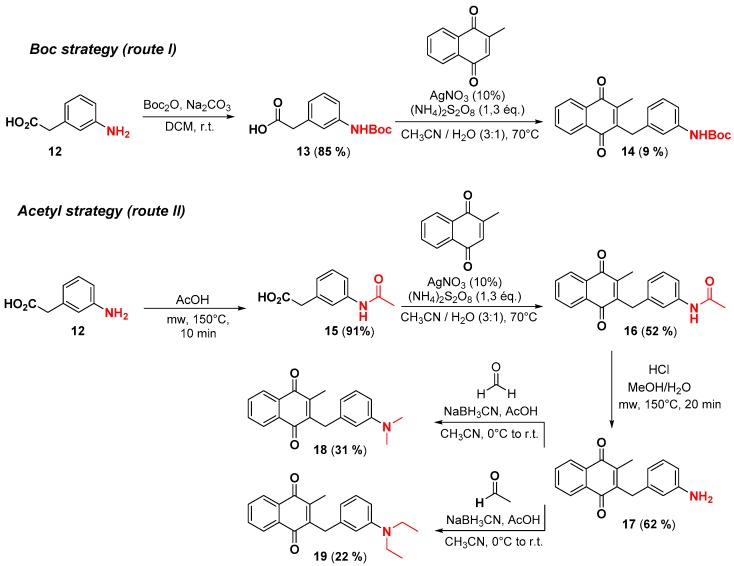
Synthesis of new 3-[3′-*N*-alkylated-benzyl]-menadione derivatives via a reductive amination procedure using the *N*-Boc or *N*-acetyl protection strategies.

We then applied the same *N*-alkylation procedures described in Scheme 4 (*N*-Boc and -acetyl protection) for the synthesis of *meta*-*N*-substituted-3-benzylmenadione derivatives **18** and **19** (Scheme 5). Following route I and starting from the commercial 3-aminophenylacetic acid (**12**), the *meta*-*N*-Boc-benzylmenadione intermediate **14** was obtained in two steps in low yield (7%) by protection of the free benzylamine group with a Boc group followed by the Kochi–Anderson reaction. As the desired compound **14** was obtained in low yield, we thought that the Boc protecting group should be changed to an acetyl group in order to increase the efficacy of this methodology when following route II.

Indeed, the *meta*-*N*-acetyl-benzylmenadione intermediate **16** was first isolated in moderate yields (47%) in two steps by *N*-acetyl protection followed by a subsequent Kochi-Anderson reaction. Then, an HCl-promoted acetyl removal followed by a reductive amination reaction of free amine **17** with formaldehyde and acetaldehyde respectively, resulted in the formation of the desired *meta*-*N*,*N*-dimethyl-3-benzylmenadione (**18**, 31%) and *meta*-*N*,*N*-diethyl-3-benzylmenadione (**19**, 22%) respectively (route II, Scheme 5).

**Scheme 6:**
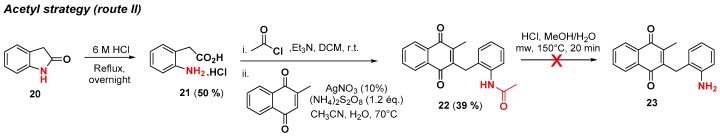
Synthesis of new 3-[2-*N*-alkylated-benzyl]-menadione derivatives via an *ortho*-*N*-alkylation procedure using the *N*-acetyl protection.

Next, we synthesized the *ortho*-*N*-substituted-3-benzylmenadione derivatives by applying the *N*-acetyl protection as depicted in Scheme 6 (route II), the *ortho*-*N*-acetyl-3-benzylmenadione intermediate was isolated in moderate yield (39%) by a sequential opening of the oxoindole [52] ring **20** to aminoacid **21** followed by a *N*-acetyl protection [53] of the generated free amine **21** and a final Kochi-Anderson reaction. To synthesize compound **23**, the optimal heating and microwave irradiation conditions for the *N*-acetyl deprotection reaction of compound **22** proved to be inappropriate, leading mainly to the degradation of the starting material **22** (Scheme 6).

### 2.2. Biological Activities

#### 2.2.1. In Vitro Antimalarial Activity

All final isolated compounds were tested for their ability to inhibit the in vitro growth of the multiresistant *P. falciparum* Dd2 strain by determining the concentration required to inhibit parasite development by 50% (IC_50_ values). The protocols are described in the experimental section. Two protocols to measure the growth inhibition were used: the tritiated hypoxanthine incorporation method and the SYBR_green_ method. This was due to the fact that the radioactive assay was abandoned in the laboratory due to cost issues related to radioactive waste disposal. The growth inhibition measured in the malarial parasite assays and the cytotoxicity against human fibroblasts of all the new compounds are listed in Table 1 and Table 2. For comparison, data for plasmodione and its bromo analogue described in our previous publications were also included in the tests. All data are mean values from one to four independent experiments, each consisting of eight drug concentrations in duplicate.

To explore the effect of modifications in plasmodione by replacing the substituted aryl by biaryl moieties bearing three distinct functionalities on antiplasmodial activity and cytotoxicity, we tested compounds **6**, **25c**, **26**–**28** in the hypoxanthine incorporation test (Table 1). Whatever the nature of the substituent (electron-donating t-Bu, electron-withdrawing NO_2_, protonable substituent NMe_2_), the potency was lower than that of plasmodione itself. The most active compound was the biaryl functionalized by a *p*-NMe_2_ exhibiting a 10-fold higher IC_50_ value (229 nM versus 29 nM for plasmodione).

This last result oriented the work towards the synthesis and testing of plasmodione analogues in which the 4′-CF_3_ was replaced by 4′-NMe_2_, 4′-NEt_2_, 3′-NMe_2_, or a 3′-NEt_2_. All intermediates (bearing NH_2_, NHBoc, NHAc, Br groups) were tested in the SYBR_green_ drug assay (Table 2). Interestingly, all benzylmenadione analogues bearing a *N*-dialkyl or a *N*-monoalkyl group in 4′ or 3′ were >10-fold less active than plasmodione itself, suggesting that the lysosomotropic compound concentration in acidic compartments was not a favourable parameter to enhance the antiplasmodial activity of redox-cyclers. In contrast, the non-protonable bromo- or the *NH*-protected amine analogues were much more potent compared to the *N*-dialkyl derivatives. The *para*- and *ortho*-substitutions were also found the most favourable in the bromo series (82 or 86 nM versus 127 nM for *para*- or *ortho*, versus -*meta*, compared to 58 nM for plasmodione). Unexpectedly, the 4′-NHBoc benzylmenadione was found very active, with an IC_50_ value of 109 nM.

#### 2.2.2. Cytotoxicity of Plasmodione Derivatives in Human Cell Assays

All compounds were tested for cytotoxicity on human lung MRC-5 fibroblasts using the Alamar Blue assay (Table 1 and Table 2). Most of the benzylmenadione derivatives exhibited a low cytotoxicity as evidenced by the high IC_50_ values above 32 μM.

#### 2.2.3. In Vivo Antimalarial Activity

The in vitro most active compound **6** among the newly synthesized derivatives was subsequently tested in the *P. berghei* mouse model using the chloroquine (CQ)-susceptible ANKA strain (Table 3). For comparative purposes, CQ was also included and dosed at 10 mg/kg intraperitoneally (ip) resulting in a decreased parasitemia by 86.3% and by 76.8% at days 4 and 7. Compound **6** showed significant activity following a 5-day treatment at 50 mg/kg ip resulting in decreased parasitaemia by 75.5% and 81.8% at days 4 and 7. For comparison, the previously described 4-bromo derivative **25c** showed 35.8% reduction of parasitemia in *P. berghei* strain ANKA-infected CD1 mice at day 4 (compound **1a** in Table 4 of [3]).

### 2.3. Physicochemical Properties

#### Acid-Base Properties

**Figure 3:**
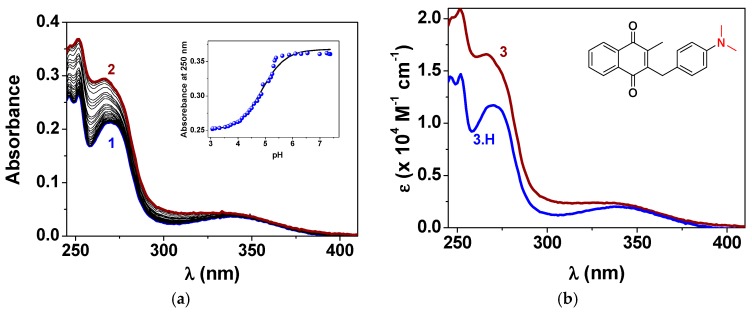
(**a**) Absorption spectrophotometric titration of compound **3** as a function of pH and (**b**) electronic spectra of **3** and its monoprotonated species **3**.H. Solvent: Water/DMSO (91/9 *v/v*); I = 0.1 M NaClO_4_; T = 25.0 ± 0.2 °C; l = 1 cm; [**3**]_tot_ = 1.81 × 10^−5^ M; (1) pH = 3.06; (2) pH = 7.25. The charges have been omitted for clarity. The inset of (A) shows the spectral variation at 250 nm as a function of pH. The UV-visible absorption spectra have not been corrected from dilution effects.

The acid-base properties of the relevant plasmodione derivatives were evaluated by absorption spectrophotometric titrations versus pH titrations. The derivatives are characterized by a set of three main absorptions centered at ~340 nm, ~270 nm and ~250 nm, respectively. The electronic transitions at low energies (>300 nm) were attributed to π-π* transitions centered on the 1,4-napthoquinone chromophore (benzoquinoidal structure), while those observed at higher energies (<300 nm) can be ascribed to π-π* benzene or benzyl subunits. Figure 3 depicts the spectral variations measured for compound **3** as a function of pH. The rise of pH does not affect the absorption of higher energies, while those centered below 300 nm are significantly affected by the acidity of the medium. These variations are in agreement with (de)protonation of the ammonium group substituted on the benzylic subunit. The absorption spectrophotometric titrations versus pH for the other plasmodione derivatives are provided as Supplementary Materials (Figures S1–S4).

**Figure 4:**
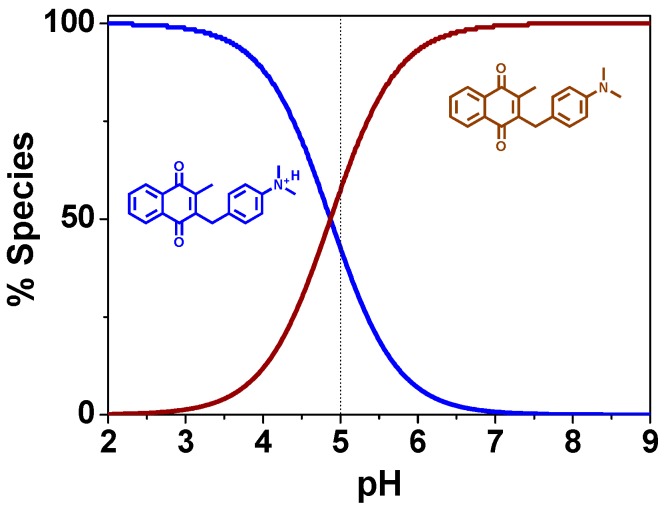
Distribution diagrams of the protonated species of plasmodione analogue **3** as a function of pH. Solvent: Water/DMSO (91/9 *v/v*); I = 0.1 M NaClO_4_; T = 25.0 ± 0.2 °C; [**3**]_tot_ = 2 × 10^−5^ M.

The statistical processing of the absorption and potentiometric datasets allowed the evaluation of a single protonation constant related to the benzylic ionizable site (i.e., benzylamine and its *N*-methylated analogues) whatever system considered (Table 4). As far as the plasmodione derivatives bearing diethyl tertiary amines are concerned (i.e., **10** and **19**), the p*K*_a_ values could not be accurately evaluated due to the precipitation at pH ~5 under our experimental conditions (H_2_O/DMSO, 91/1 *v/v*). This is likely due to the higher hydrophobic character brought by the ethyl substituent. The p*K*_a_ values ranged from 4.3 (compound **17**) to 5.07 (compound **11**) and are in excellent agreement with the values determined for adequate models such as aniline (p*K*_a_ = 4.72 [54] in 0.1 M KCl versus compound **7** p*K*_a_ = 4.74 ± 0.05 or compound **17** p*K*_a_ = 4.30 ± 0.02) or *N*-phenyldiethanolamine (p*K*_a_ = 4.13 [55] in 0.4 M KCl versus compound **3** p*K*_a_ = 4.87 ± 0.03 or compound **18** p*K*_a_ = 4.60 ± 0.04). This feature emphasizes the absence of any electronic effect of the menadione core on the acido-basic properties of the benzylamine subunit due to the presence of the methylenic moiety which can be regarded as a poor electronic relay. Taken as an example, Figure 4 depicts the distribution diagrams of the protonated species of compound **3** clearly showing that below pH 5 (anticipated pH of the food vacuole of *P. falciparum*) the plasmodione analogues bearing an amino substitution of a benzyl position exist predominantly in their ammonium form. Table 4 provides the ratios of the protonated species at pH 5 and 7.4 clearly evidencing accumulation in the food vacuole due to pH gradient.

## 3. Discussion

In an attempt to generate plasmodione analogues with improved activity/solubility profiles, we developed synthetic methodologies to prepare a biaryl series through a 2-step sequence using Kochi-Anderson reaction followed by a Pd-catalyzed Suzuki-Miyaura cross-coupling, and a second 3-benzylmenadione series bearing a terminal -*N*(Me)_2_ or -*N*(Et)_2_ in different positions (*ortho*, *meta* and *para)* on the aryl ring of the benzylic chain of plasmodione through reductive amination, as the optimal route to prepare these protonable *N*-arylalkylamines.

**Figure 5:**
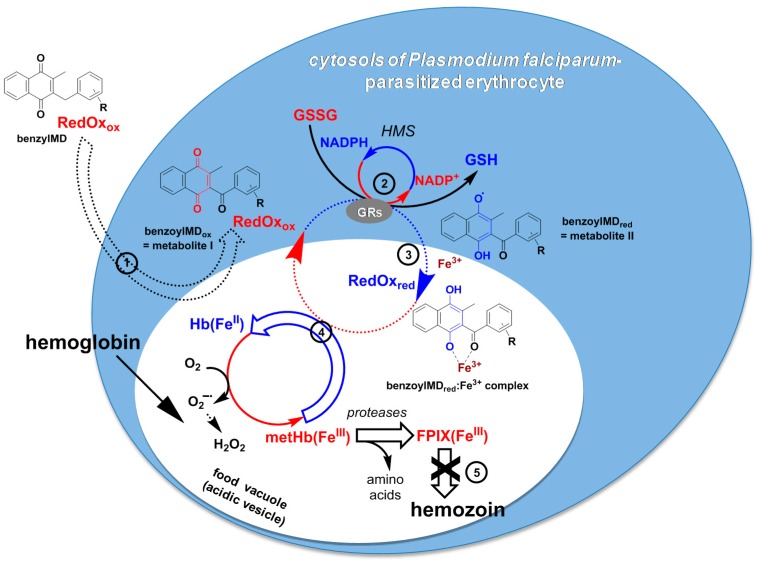
Putative model for cytosol-food vacuole shuttling of 3-benz(o)ylmenadione derivatives during redox-cycling affecting the redox homeostasis in the cytosols of *P. falciparum*-parasitized erythrocytes through a cascade of redox reactions accounting for the observed antimalarial activity. Blue arrows indicate reduction; red arrows oxidation; dashed arrows uptake processes. The lead 3-benzylmenadione is proposed to be taken up by infected red blood cells (step 1), be reduced in the cytosols of parasitized erythrocytes by human GR (step 2), and then be oxidized at the benzylic chain to 3-benz(o)ylmenadione (3-benz(o)ylMD) in the acidic vesicles or in the food vacuole in heme-catalyzed reactions (not shown). The reduced species of 3-benz(o)ylmenadione are subsequently assumed to be transported through Fe^III^ complexation into the acidic vesicles (step 3) where reduced species of 3-benz(o)ylmenadione transfer the electrons to oxidants (hematin or metHb, step 4). The final result is an inhibition of hemozoin formation (step 5) and the arrest of parasite development as shown previously [3]. Hence, the antimalarial benzylmenadione derivatives would act as prodrugs of redox-active principles, being cycled in and out of the acidic vesicles in infected red blood cells, thereby oxidizing major intracellular reductants (NADPH) and subsequently reducing oxidants like hematin or metHb. HMS, hexose monophosphate shunt.

From the mechanistic point of view, the benzoylmenadiones were proposed to be the key potential metabolites acting in oxidized form as the most efficient subversive substrates of reduced GR described so far and, in reduced form, to transfer electrons to methemoglobin. The reduced species of benzoylmenadiones are assumed to be transported through Fe^III^ complexation into the food vacuole where the electrons are transferred to oxidants (heme and methemoglobin). Regenerated in their oxidized form, the benzoylmenadiones might be transported into the cytosol where they are reduced by the cytosolic GR (either from human erythrocyte or from the parasite) in a continuous redox cycle (Figure 5). Evidence for cytosol-food vacuole shuttling of plasmodione during redox-cycling is difficult to prove, however, replacing the 4′-CF_3_ group in plasmodione by protonable terminal -*N*(Me)_2_ or -*N*(Et)_2_, the aim was to vectorize the final derivatives to the acidic compartments of the malarial parasite, e.g., the food vacuole, where hemoglobin digestion takes place. By partly losing antimalarial potency, these compounds provide indirect proof for the hypothesized cytosol-food vacuole shuttling of plasmodione during redox-cycling. Worthy of mention is the fact that the redox-active MB, which possesses potent antimalarial activity, shares similar physico-biochemical properties to those found for plasmodione and its active analogues, i.e., non-protonable *N*-dimethylamino groups at the acidic pH of the food vacuole, redox potential values in the same range, NADPH-dependent methemoglobin redox-cycling in the presence of the redox-active blue dye [3,29,33].

The most active derivatives presented in this work are the non-protonable plasmodione analogues, suggesting that lysosomotropic properties are not favourable to optimize the redox-active compounds that are cycled in and out of the food vacuole in parasitized erythrocytes.

## 4. Materials and Methods

### 4.1. General Information

#### 4.1.1. Solvents and Reagents

Commercially available starting materials were purchased from Sigma-Aldrich (St. Louis, MO, USA), ABCR GmbH & Co. KG (Karlsruhe, Germany), Alfa Aesar (Haverhill, MA, USA), and Apollo Scientific (Cheshire, UK) and were used without further purification. Solvents were obtained from Sigma-Aldrich and Carlos Erba (Val de Reuil, France). Unless noted, reagent grade chemicals were used for reactions and analytical grade ones for column chromatography and recrystallizations. When specified, anhydrous solvents were required; dichloromethane (DCM) was distilled over CaH_2_ under argon. Tetrahydrofuran (THF) was distilled over sodium/benzophenone under argon or dried by passage through an activated alumina column under argon. 1,4-Dioxane and dimethylformamide (DMF) were purchased anhydrous over molecular sieves from Sigma-Aldrich. (St. Louis, MO, USA) Triethylamine (Et_3_N), diisopropylethyl amine (DIPEA), pyrrolidine, piperidine were distilled over KOH under argon and stored over KOH. All reactions were performed in standard glassware. Microwave reactions were carried out on two different apparatus (Biotage Initiator™, Uppsala, Sweden and CEM, Orsay, France) with comparable results (cross compared); supplier standard microwave vials were used. Crude mixtures were purified either by recrystallization or by flash column chromatography. The latter were performed using silica gel 60 (230–400 mesh, 0.040–0.063 mm) purchased from E. Merck (Kenilworth, NJ, USA). Automatic flash chromatographies were carried out in a Biotage Puriflash apparatus (Uppsala, Sweden) with UV-Vis detection at 254 nm (unless otherwise specified). Monitoring and primary characterization of products were achieved by TLC on aluminium sheets coated with silica gel 60 F254 purchased from E. Merck. Eluted TLC’s were revealed under UV (325 nm and 254 nm) and with chemicals. Analytical TLC was carried out on pre-coated Sil G-25 UV_254_ plates from Macherey Nagel (Hoerdt, France). Flash chromatography was performed using silica gel G60 (230–400 mesh) from Macherey Nagel.

#### 4.1.2. Instruments

The Nuclear Magnetic Resonance (NMR, ^1^H-NMR 300 MHz, ^13^C-NMR 75 MHz) spectra were registered on a Bruker Avance 300 apparatus (Wissembourg, France). A Bruker Avance 400 apparatus was used (^1^H-NMR 400 MHz, ^13^C-NMR 100 MHz) for more complex spectra. All examples below are labelled as 300/75 MHz except for a few ^13^C spectra (compounds **7**, **18**, **22**). All chemical shifts (δ) are quoted in parts per million (ppm). The chemical shifts are referred to the used partial deuterated-NMR solvent (for CDCl_3_: ^1^H-NMR, 7.26 ppm and ^13^C-NMR, 77.16 ppm). The coupling constants (*J*) and the non-equivalence (Δν) are given in Hertz (Hz). Resonance patterns are reported with the following notations: br (broad), s (singlet), d (doublet), t (triplet), q (quartet), m (multiplet), dd (doublet of doublets), AB (AB system), (ABX) (AB system of an ABX) and A_2_B_2_ (A_2_B_2_ aromatic system). In addition, the following acronyms will be used: C=O carbonyl group; C_q_: quaternary carbon; CH_2_: secondary carbon; CH_3_: methyl group. Elemental analyses (EA) were obtained at “Service de Microanalyses” at the Institut de Chimie de Strasbourg. Mass spectra (ESI-MS) were obtained on a microTOF LC spectrometer (Bruker Daltonics, Bremen, Germany). High Resolution Mass (HRMS) spectra were measured and fitted with calculated data. Melting points (M.p.) were determined on a Büchi (Rungis, France) melting point apparatus and were not corrected.

### 4.2. Protection of Aminophenylacetic acids with Boc Groups (Synthesis of ***5*** and ***13***)

**Scheme 7:**
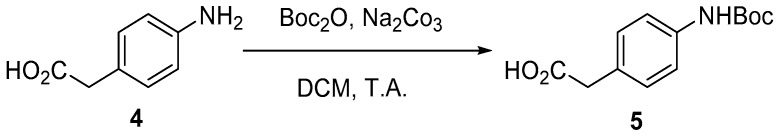
Synthetic chemical route to compound **5**.

*2-(4-((tert-Butoxycarbonyl)amino)phenyl)acetic Acid* (**5**)*.* A solution of 4-aminophenylacetic acid (**4**, 4 g, 26.46 mmol, 1 equiv.) in a mixture of dioxane (52 mL) and water (52 mL), and sodium carbonate (2.8 g, 26.42 mmol, 1 equiv., in 26 mL of water) was stirred and cooled in an ice bath. Di-*tert*-butyl dicarbonate (BOC-anhydride, 6.24 g, 28.59 mmol, 1.1 equiv.) was added in one portion, and stirring was continued at room temperature for 4 h. The dioxane was removed in vacuo and the aqueous layer chilled, covered with a layer of ethyl acetate, and acidified to pH 4 with dilute KHSO_4_. This was followed by extraction (ethyl acetate) and purification (1:1:1) of ethyl acetate–hexane–acetic acid; ethyl acetate) to yield the compound **5** (Scheme 7; 7.07 g; 94%) as a white solid. ^1^H-NMR (300 MHz, CDCl_3_): 7.29 (d, *J* = 8.40 Hz, 2H), 7.19 (d, *J* = 8.47 Hz, 2H), 3.70 (s, 1H), 3.58 (s, 2H), 1.52 (s, 9H).

**Scheme 8:**
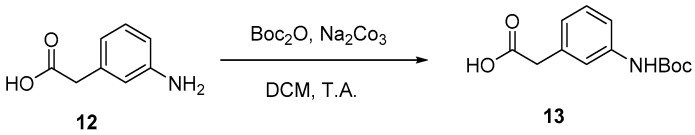
Synthetic chemical route to compound **13**.

*2-(3-((tert-Butoxycarbonyl)amino)phenyl)acetic Acid* (**13**)*.* A solution of 3-aminophenylacetic acid (**12**, 4 g, 26.46 mmol, 1 equiv.) in a mixture of dioxane (52 mL) and water (52 mL), and sodium carbonate (2.8 g, 26.42 mmol, 1 equiv., in 26 mL of water) was stirred and cooled in an ice bath. Di-*tert*-butyl dicarbonate (BOC-anhydride, 6.24 g, 28.59 mmol, 1.1 equiv.) was added in one portion, and stirring was continued at room temperature for 4 h. The dioxane was removed in vacuo and the aqueous layer chilled, covered with a layer of ethyl acetate, and acidified to pH 4 with dilute KHSO_4_. This was followed by extraction (ethyl acetate) and purification by a filtration on silica with dichloromethane followed by ethyl acetate to yield the compound **13** (Scheme 8; 6.65 g; 85%) of a white solid. ^1^H-NMR (300 MHz, CDCl_3_): 7.31 (d, 2H, *J* = 8.4 Hz), 7.20 (d, 2H, *J* = 8.4 Hz), 6.56 (br s, 1H), 3.59 (s, 2H), 1.5 (s, 9H).

### 4.3. Protection of Aminophenylacetic Acids with Acetyl Groups (Synthesis of ***8**, **15*** and ***21a***)

**Scheme 9:**
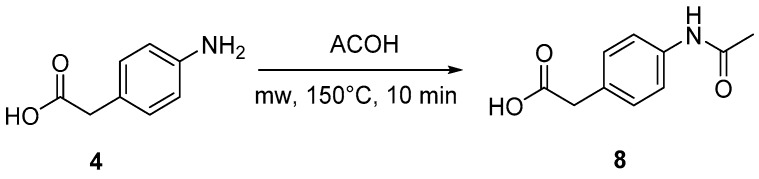
Synthetic chemical route to compound **8**.

*2-(4-Acetamidophenyl)acetic Acid* (**8**)*.* According to a reported procedure [49], 4-aminophenylacetic acid (**4**, 2.0 g) was added to acetic acid (20 mL) under microwave irradiation (150 °C) for 1 h. The reaction mixture was poured into ethyl acetate then washed with water and then allowed to dry to yield the compound **8** (Scheme 9; 2.252 g; 100%) as a white-grey solid. m.p. 166–168 °C [57]. ^1^H-NMR (300 MHz, DMSO-*d*_6_): δ = 9.92 (s, 1H), 7.51 (d, ^3^*J* = 8.6 Hz, 2H), 7.16 (d, ^3^*J* = 8.6 Hz, 2H), 3.49 (s, 1H), 2.03 (s, 3H). ^13^C-NMR (75 MHz, DMSO-*d*_6_): δ = 172.79 (C_q_), 172.02 (C_q_), 168.13 (C_q_), 137.83 (C_q_), 129.52 (CH), 118.87 (CH), 40.11 (CH_2_), 23.87 (CH_3_). IR: 3348 (w), 1709 (s), 1638 (m), 1601 (s), 1542 (s), 1538 (s), 1221 (m), 1195 (m), 968 (w), 806 (w), 723 (w). ^1^H-NMR (400 MHz, CD_3_OD): δ 7.48 (d, *J* = 8.8 Hz, 2H), 7.21 (d, *J* = 8.8 Hz, 2H), 3.55 (s, 2H), 2.10 (s, 3H); HRMS (FAB^+^): [M + H]^+^ calcd. for C_10_H_12_O_3_N, 194.0817; found, 194.0770.

**Scheme 10:**
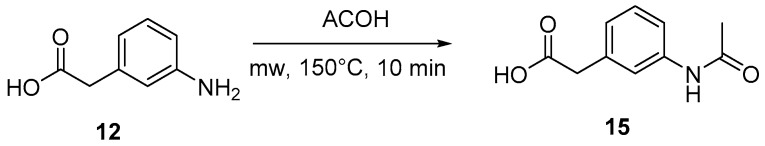
Synthetic chemical route to compound **15**.

*2-(3-Acetamidophenyl)acetic Acid* (**15**)*.* 3-Aminophenylacetic acid (**12**, 200 mg) was added to acetic acid (2 mL) under microwave irradiation (150 °C) for 10 min. The reaction mixture was poured into a mixture of ethyl acetate (1:50), then washed with water and then allowed to dry to yield the compound **15** (Scheme 10) (232 mg; 91%) as a white solid. m.p. 128–129 °C. ^1^H-NMR (300 MHz, DMSO-*d*_6_): δ = 12.27 (s, 1H), 9.90 (s, 1H), 7.47 (dd, ^3^*J* = 2.32 Hz and 4.57 Hz, 1H), 7.24–7.19 (mc, 1H), 6.91 (d, ^3^*J* = 7.70 Hz, 1H), 3.51 (s, 2H), 2.03 (s, 3H). ^13^C-NMR (75 MHz, DMSO-*d*_6_): δ = 172.52 (C_q_), 168.21 (C_q_), 139.26 (C_q_), 135.39 (C_q_), 128.48 (C_q_), 124.00 (C_q_), 119.76 (C_q_), 117.32 (C_q_), 40.86 (CH_2_), 23.94 (CH_3_). IR: 3298 (w), 1699 (s), 1662 (s), 1544 (m), 1492 (m), 1411 (m), 1308 (m), 1273 (m), 1232 (m), 886 (m), 782 (s), 714 (s).

**Scheme 11:**
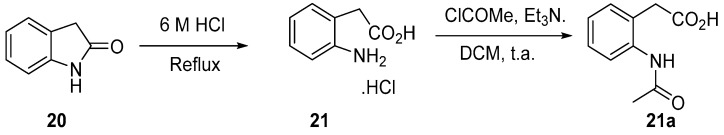
Synthetic chemical route to compound **21a**.

*2-(2-Acetamidophenyl)acetic Acid* (**21a**). Oxindole **20** (2.5 g, 14.7 mmol, 1 equiv.) was diluted in 6 M HCl (15 mL) and heated to reflux with stirring overnight. The solution was cooled and washed with dichloromethane (3 × 100 mL). The aqueous phase was concentrated to give **21** as a white solid (2.73 g, 99%). The crude product was involved directly in the next step. A solution of 2-aminophenylacetic acid hydrochloride (1.5 g, 8 mmol, 1 equiv.) in dichloromethane (110 mL) at room temperature was treated with triethylamine (2.35 mL, 17.6 mmol, 2.2 equiv.) and acetyl chloride (682 µL, 9.6 mmol, 1.2 equiv.). The reaction mixture was stirred at r.t. for 3 h. The reaction mixture was diluted with EtOAc and washed with water. The aqueous layer was extracted with ethyl acetate. The combined extracts were rinsed with brine, dried over MgSO_4_. Purification on silica gel with cyclohexane/ethyl acetate (20:5 to 1:1) gives the desired product **21a** (Scheme 11; 160 mg, 10%). m.p. 130–131 °C. ^1^H-NMR (300 MHz, CDCl_3_): δ = 8.21 (d, ^3^*J* = 8.29 Hz, 1H), 7.34–7.25 (m, 2H), 7.20–7.15 (m, 1H), 3.71 (s, 2H), 2.67 (s, 3H). ^13^C-NMR (75 MHz, CDCl_3_): δ = 175.47 (C_q_), 171.03 (C_q_), 141.54 (C_q_), 128.37 (CH), 125.09 (CH), 124.10 (CH), 123.57 (C_q_), 116.82 (CH), 36.80 (CH_2_), 26.83 (CH_3_).

### 4.4. General Procedure for the Kochi-Anderson Reaction of Menadione with N-Protected Phenylacetic Acid Derivatives (Synthesis of ***6**, **14**, **9**, **16**, **22*** and ***25***)

**Scheme 12:**
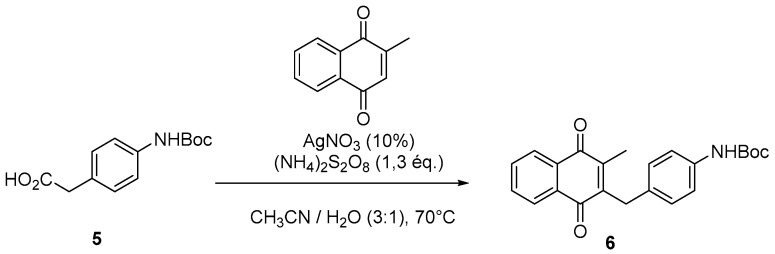
Synthetic chemical route to compound **6**.

*tert-Butyl(4-((3-Methyl-1,4-dioxo-1,4-dihydronaphthalen-2-yl)methyl)phenyl) carbamate* (**6**)*.* A solution of menadione (500 mg, 2.9 mmol, 1.0 equiv.) and phenylacetic acid **5** (1.455 g, 5.8 mmol, 2.0 equiv.) in acetonitrile (27 mL) and water (9 mL) was heated to 70 °C. AgNO_3_ (50 mg, 0.29 mmol, 0.1 equiv.) was added. (NH_4_)_2_S_2_O_8_ (860 mg, 3.77 mmol, 1.3 equiv.) in acetonitrile (3 mL) and water (1 mL) was added dropwise over a period of 45 min and then heated at reflux for 2 h. The acetonitrile was removed in vacuo. The product was extracted with EtOAc (1 × 10 mL), dichloromethane (4 × 10 mL), dried over MgSO_4_ and purified by chromatography on silica gel (cyclohexane/dichloromethane 30:5) to give the pure compound **6** (Scheme 12; 243 mg; 22% yield) as a yellow solid. m.p. 148–149 °C. ^1^H-NMR (300 MHz, CDCl_3_): δ = 8.05–8.08 (m, 2H), 7.66–7.71 (m, 2H), 7.23 (d, ^3^*J* = 8.26 Hz, 2H), 7.12 (d, ^3^*J* = 8.57 Hz, 2H), 6.36 (s, 1H), 3.95 (s, 2H), 2.21 (s, 3H), 1.47 (s, 9H). ^13^C-NMR (75 MHz, CDCl_3_): δ = 185.42 (C_q_), 184.68 (C_q_), 152.73 (C_q_), 145.38 (C_q_), 144.21 (C_q_), 136.70 (C_q_), 133.47 (CH), 133.44 (CH), 132.61 (C_q_), 132.10 (C_q_), 132.03 (C_q_), 129.14 (CH), 126.45 (CH), 126.25 (CH), 118.88 (CH), 80.49 (C_q_), 31.74 (CH_2_), 28.30 (CH_3_), 13.22 (CH_3_). EI MS (70 eV, *m/z* (%)): 377.2 ([M]^+^, 18), 321.1 (66), 305.9 (100), 261.1 (59), 201.3 (14), 160.1 (18), 121.1 (21). IR: 3439 (b, vs), 1704 (w), 1685 (w), 1660 (s), 1618 (m), 1596 (m), 1521 (m), 1370 (w), 1315 (m), 1296 (m), 1236 (w), 1162 (s), 709 (w). EA: obs. C, 72.94%; H, 6.16%; N, 3.74%, calcd. C, 73.19%; H, 6.14%, N, 3.71% for C_23_H_23_NO_4_.

**Scheme 13:**
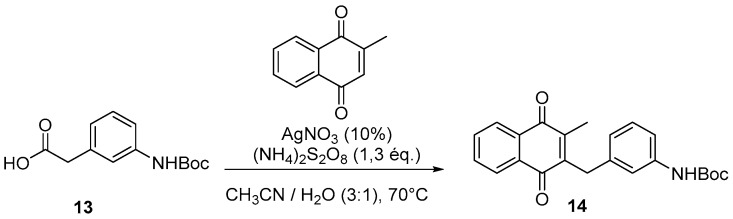
Synthetic chemical route to compound **14**.

*tert-Butyl (3-((3-Methyl-1,4-dioxo-1,4-dihydronaphthalen-2-yl)methyl)phenyl) carbamate* (**14**)*.* A solution of menadione (1.71 g, 9.93 mmol, 1.0 equiv.) and phenylacetic acid **13** (5.0 g, 19.9 mmol, 2.0 equiv.) in acetonitrile (36 mL) and water (12 mL) was heated to 70 °C. AgNO_3_ (173 mg, 0.29 mmol, 0.1 equiv.) was added. (NH_4_)_2_S_2_O_8_ (2.955 g, 3.77 mmol, 1.3 equiv.) in acetonitrile (11 mL) and water (3.5 mL) was added dropwise over a period of 45 min and then heated at reflux for 3 h. The acetonitrile was removed in vacuo. The product was extracted with EtOAc (2 × 10 mL), dichloromethane (4 × 10 mL), dried over MgSO_4_ and purified by chromatography on silica gel (toluene) a second chromatography on silica was necessary (cyclohexane/dichloromethane 10:1) to give the pure compound **14** (Scheme 13; 348 mg; 9% yield) as a yellow solid. ^1^H-NMR (300 MHz, CDCl_3_): δ = 8.10–8.05 (m, 2H), 7.72–7.66 (m, 2H), 7.28–7.26 (m, 1H), 7.20–7.14 (m, 2H), 6.86 (d, *J* = 7.52 Hz, 1H), 6.46 (s, 1H), 3.99 (s, 2H), 2.24 (s, 3H), 1.49 (s, 9H). ^13^C-NMR (75 MHz, CDCl_3_): δ = 185.45 (C_q_), 184.73 (C_q_), 152.73 (C_q_), 145.24 (C_q_), 144.70 (C_q_), 139.04 (C_q_), 138.76 (C_q_), 133.58 (CH), 133.57 (CH), 132.23 (C_q_), 132.12 (C_q_), 129.37 (CH), 126.60 (CH), 126.38 (CH), 123.31 (CH), 118.81 (CH), 116.79 (CH), 80.60 (CH), 32.48 (CH_2_), 28.43 (CH_3_), 13.44 (CH_3_). HRMS-ESI (*m/z*): [M + Na]^+^ calcd. for C_23_H_23_NO_4_Na 400.1519; found 400.1539.

**Scheme 14:**
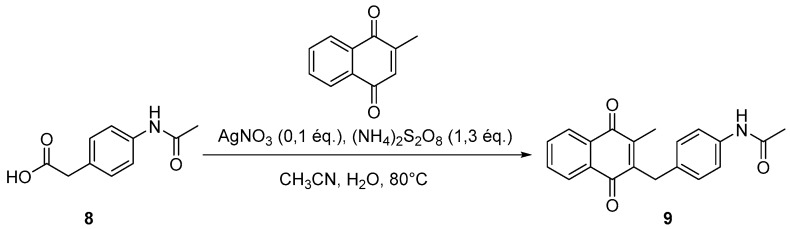
Synthetic chemical route to compound **9**.

*N-(4-((3-Methyl-1,4-dioxo-1,4-dihydronaphthalen-2-yl)methyl)phenyl)acetamide* (**9**)*.* A solution of menadione (600 mg, 3.48 mmol, 1.0 equiv.) and phenylacetic acid **8** (1.3 g, 6.72 mmol, 2.0 equiv.) in acetonitrile (12 mL) and water (4 mL) was heated to 70 °C. AgNO_3_ (59 mg, 0.35 mmol, 0.1 equiv.) was added. (NH_4_)_2_S_2_O_8_ (1.032 g, 4.52 mmol, 1.3 equiv.) in 4 mL acetonitrile and 2 mL water was added dropwise over a period of 45 min and then heated at reflux for 3 h. The acetonitrile was removed in vacuo. The product was extracted with dichloromethane (4 × 10 mL), dried over MgSO_4_ and purified by chromatography on silica gel (cyclohexane/ethyl acetate 80:20 to cyclohexane/ethyl acetate 50:50) to give the pure compound **9** (Scheme 14; 597 mg; 54% yield) as a yellow solid. m.p. 198–200 °C. ^1^H-NMR (300 MHz, CDCl_3_): δ = 8.12–8.05 (m, 2H), 7.72–7.67 (m, 2H), 7.38 (d, ^3^*J* = 8.40 Hz, 2H), 7.17 (d, ^3^*J* = 8.40 Hz, 2H), 3.99 (s, 2H), 2.24 (s, 3H), 2.14 (s, 3H). ^13^C-NMR (75 MHz, CDCl_3_): δ = 185.50 (C_q_), 184.80 (C_q_), 168.28 (C_q_), 145.38 (C_q_), 144.49 (C_q_), 136.39 (C_q_), 134.13 (C_q_), 133.64 (CH), 132.27 (C_q_), 132.17 (C_q_), 129.29 (CH), 126.60 (CH), 126.44 (CH), 120.36 (CH), 32.01 (CH_2_), 24.67 (CH_3_), 13.40 (CH_3_); IR: 3359 (w), 1690 (s), 1661 (s), 1648 (s), 1593 (m), 1533 (s), 1411 (w), 1371 (m), 1334 (m), 1311 (s), 1295 (vs), 1259 (m), 972 (w), 818 (m), 735 (m), 709 (s), 679 (w).

**Scheme 15:**
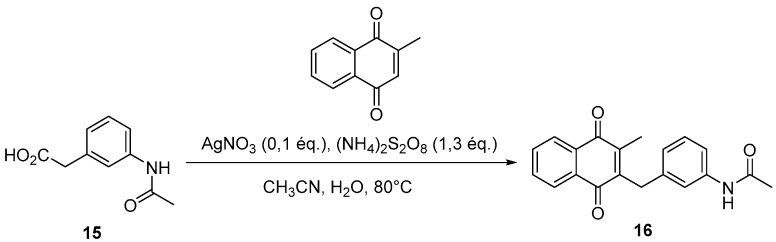
Synthetic chemical route to compound **16**.

*N-(3-((3-Methyl-1,4-dioxo-1,4-dihydronaphthalen-2-yl)methyl)phenyl)acetamide* (**16**)*.* A solution of menadione (380 mg, 2.1 mmol, 1.0 equiv.) and phenylacetic acid **15** (812 mg, 4.2 mmol, 2.0 equiv.) in acetonitrile (6 mL) and water (2 mL) was heated to 70 °C. AgNO_3_ (36 mg, 0.21 mmol, 0.1 equiv.) was added. (NH_4_)_2_S_2_O_8_ (623 mg, 2.73 mmol, 1.3 equiv.) in acetonitrile (1.5 mL) and water (0.5 mL) was added dropwise over a period of 45 min and then heated at reflux for 3 h. The acetonitrile was removed in vacuo. The product was extracted with dichloromethane (4 × 10 mL), dried over MgSO_4_ and purified by chromatography on silica gel (cyclohexane/ethyl acetate 10:1) to give the pure compound **16** (Scheme 15; 670 mg; 52% yield) as a yellow solid. m.p. 163–165 °C. ^1^H-NMR (300 MHz, CDCl_3_): δ = 8.10–8.03 (m, 2H), 7.71–7.65 (m, 2H), 7.41 (d, ^3^*J* = 8.30 Hz, 1H), 7.32 (s, 2H), 7.20 (t, ^3^*J* = 7.82 Hz, 1H), 6.96 (d, ^3^*J* = 7.80 Hz, 1H), 3.99 (s, 2H), 2.24 (s, 3H), 2.12 (s, 3H). ^13^C-NMR (75 MHz, CDCl_3_): δ = 185.41 (C_q_), 184.79 (C_q_), 168.41 (C_q_), 145.15 (C_q_), 144.80 (C_q_), 139.09 (C_q_), 138.36 (C_q_), 133.63 (CH), 132.25 (C_q_), 132.12 (C_q_), 129.41 (CH), 126.59 (CH), 126.43 (CH), 124.59 (CH), 119.99 (CH), 118.16 (CH), 32.50 (CH_2_), 24.71 (CH_3_), 13.44 (CH_3_); IR: 1661 (m), 01609 (w), 1551 (w), 1490 (w), 1374 (w), 1319 (w), 1293 (m), 726 (w).

**Scheme 16:**
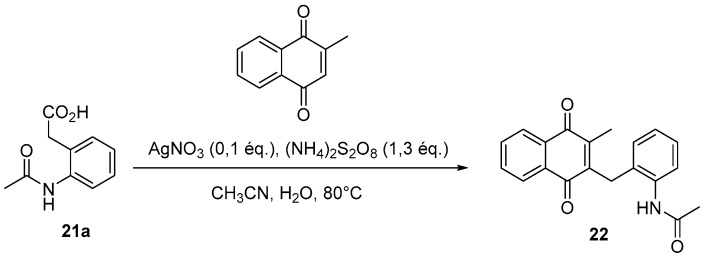
Synthetic chemical route to compound **22**.

*N-(2-((3-Methyl-1,4-dioxo-1,4-dihydronaphthalen-2-yl)methyl)phenyl)acetamide* (**22**)*.* A solution of 2-aminophenylacetic acid hydrochloride (**21a**, 4.55 g, 24.25 mmol, 1 equiv.) in dichloromethane (110 mL) at room temperature was treated with triethylamine (7.15 mL, 48.5 mmol, 2.2 equiv.) and acetyl chloride (2.06 mL, 29.12 mmol, 1.2 equiv.). The reaction mixture was stirred at r.t. for 3 h. The reaction mixture was diluted with EtOAc and washed with water. The aqueous layer was extracted with ethyl acetate. The combined extracts were rinsed with brine, dried over MgSO_4_. Thus, a solution of the crude and menadione (890 mg, 5.17 mmol, 1.0 equiv.) in acetonitrile (32 mL) and water (11 mL) was heated to 70 °C. AgNO_3_ (88 mg, 0.52 mmol, 0.1 equiv.) was added. (NH_4_)_2_S_2_O_8_ (1.533 g, 6.72 mmol, 1.3 equiv.) in acetonitrile (10 mL) and water (5 mL) was added dropwise over a period of 45 min and then heated at reflux for 3 h. The acetonitrile was removed in vacuo. The product was extracted with dichloromethane (4 × 10 mL), dried over MgSO_4_ and purified by chromatography on silica gel (cyclohexane/ethyl acetate 10:1 to cyclohexane/ethyl acetate 5:5) to give the pure compound **22** (Scheme 16. 673 mg; 39% yield) as a yellow solid. m.p. 218–220 °C. ^1^H-NMR (300 MHz, CDCl_3_): δ = 8.99 (s, 1H), 8.11–8.05 (m, 2H), 7.86 (d, ^3^*J* = 8.01 Hz, 1H), 7.73–7.70 (m, 2H), 7.25–7.19 (m, 2H), 7.07–7.02 (m, 1H), 3.95 (s, 2H), 2.43 (s, 3H), 2.31 (s, 3H). ^13^C-NMR (100 MHz, CDCl_3_): δ = 186.32 (C_q_), 184.90 (C_q_), 168.86 (C_q_), 145.77 (C_q_), 145.07 (C_q_), 135.98 (C_q_), 134.12 (CH), 133.70 (CH), 132.10 (C_q_), 131.81 (C_q_), 129.95 (CH), 129.24 (C_q_), 127.72 (CH), 126.63 (CH), 125.08 (CH), 124.74 (CH), 28.43 (CH_2_), 24.39 (CH_3_), 14.14 (CH_3_). IR: 3394 (w), 1674 (m), 1660 (s), 1505 (m), 1467 (m), 1295 (vs), 794 (m), 762 (s), 716 (s), 690 (m).

**Scheme 17:**
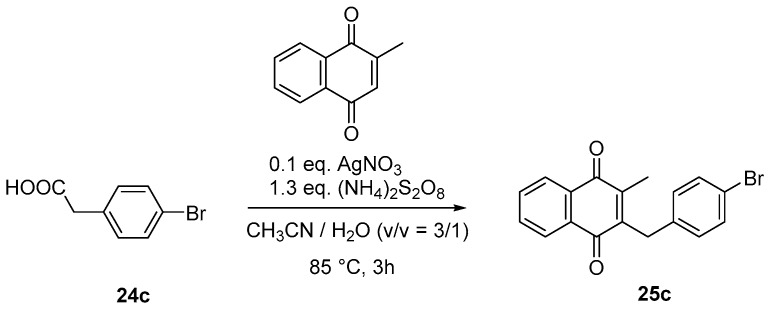
Synthetic chemical route to compound **25c**.

*2-Methyl-3-(4-bromo-benzyl)-4a,8a-dihydro-[1,4]naphthoquinone* (**25c**). A solution of menadione (5.81 mmol) and a phenylacetic acid derivative **24c** (11.58 mmol) in a mixture of (52.5 mL of acetonitrile and 17.5 mL of water) was heated to 85 °C. Then AgNO_3_ (90 mg, 0.58 mmol) was added and (NH_4_)_2_S_2_O_8_ (1.72 g, 7.54 mmol) in 15 mL of acetonitrile and 5 mL of water was added dropwise over a period of 45 min and then heated at reflux for two hours. The acetonitrile was removed in vacuo. The aqueous phase was extracted with dichloromethane (4 × 10 mL), dried over MgSO_4_ and purified by flash-chromatography on silica gel (petroleum ether/dichloromethane 1:1) to give the compound **25c** (Scheme 17; 3.10 g; 9.12 mmol, 78% yield) as a yellow solid. m.p. 121–122 °C. ^1^H-NMR (300 MHz, CDCl_3_): δ = 8.03–8.10 (m, 2H), 7.66–7.71 (m, 2H), 7.36 (dt, ^3^*J* = 8.46 Hz, ^4^*J* = 1.95 Hz, 2H), 7.09 (d, ^3^*J* = 8.53 Hz, 2H), 3.96 (s, 2H), 2.22 (s, 3H). ^13^C-NMR (75 MHz, CDCl_3_): δ = 185.20 (C_q_), 184.54 (C_q_), 144.75 (C_q_), 144.57 (C_q_), 137.06 (C_q_), 133.58 (CH), 132.08 (C_q_), 131.94 (C_q_), 131.71 (CH), 130.32 (CH), 126.50 (CH), 126.35 (CH), 120.31 (C_q_), 31.93 (CH_2_), 13.31 (CH_3_). EI MS (70 eV, *m/z* (%)): 340.1 ([M]^+^, 13), 325.0 (100), 246.1 (63), 215.1 (41), 202.1 (49), 128.1 (72), 76.0 (74). IR (KBr): 3449 cm^−1^ (b, w), 3068 (w), 2962 (w), 1661 (vs), 1624 (m), 1618 (m), 1594 (s), 1486 (s), 1376 (m), 1332 (s), 1315 (s), 1294 (vs), 1071 (m), 1010 (s), 971 (w), 815 (m), 787 (s), 730 (m), 702 (m), 629 (w), 426 (w). EA: obs. C, 63.02%; H, 3.84%, calcd. C, 63.36%; H, 3.84% for C_18_H_13_BrO_2_.

**Scheme 18:**
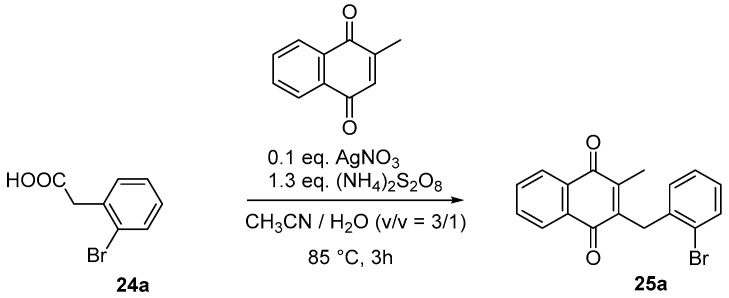
Synthetic chemical route to compound **25a**.

*2-Methyl-3-(2-bromo-benzyl)-4a,8a-dihydro-[1,4]naphthoquinone* (**25a**). After chromatography on silica gel (petroleum ether/dichloromethane 1:1), 1.75 g (5.14 mmol, 88% yield) of **25a** (Scheme 18) were isolated as a yellow solid. m.p. 94–95 °C. ^1^H-NMR (300 MHz, CDCl_3_): δ = 8.06–8.11 (m, 2H), 7.66–7.72 (m, 2H), 7.56 (dd, ^3^*J* = 7.87 Hz, ^4^*J* = 1.32 Hz, 1H), 7.13 (dt, ^3^*J* = 7.47 Hz, ^4^*J* = 1.35 Hz, 1H), 7.04 (dt, ^3^*J* = 7.69 Hz, ^4^*J* = 1.75 Hz, 1H), 6.89 (dd, ^3^*J* = 7.61 Hz, ^4^*J* = 1.56 Hz, 1H), 4.11 (s, 2H), 2.10 (s, 3H). ^13^C-NMR (75 MHz, CDCl_3_): δ = 184.98 (Cq), 184.30 (Cq), 145.85 (Cq), 144.53 (Cq), 137.31 (Cq), 133.55 (CH), 133.53 (CH), 132.87 (CH), 132.13 (Cq), 131.96 (Cq), 128.59 (CH), 127.93 (CH), 127.55 (CH), 126.53 (CH), 126.33 (CH), 124.67 (Cq), 32.65 (CH_2_), 13.26 (CH_3_). EI MS (70 eV, *m/z* (%)): 261.1 ([M − Br]^+^, 100), 231.1 (11), 202.1 (11), 130.1 (8), 76.0 (10). IR: 3441 cm^−1^ (b, s), 3068 (w), 3017 (w), 2923 (w), 1660 (vs), 1621 (s), 1594 (s), 1467 (m), 1439 (m), 1376 (w), 1318 (m), 1296 (vs), 1263 (m), 1223 (w), 1184 (w), 1025 (m), 976 (m), 787 (w), 749 (s), 729 (m), 695 (w), 663 (w). EA: obs. C, 63.12%; H, 3.91%; Br, 23.31%, calcd. C, 63.36%; H, 3.84%; Br, 23.42% for C_18_H_13_BrO_2_.

**Scheme 19:**
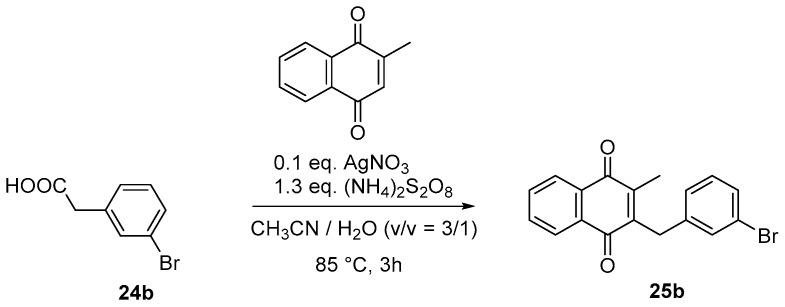
Synthetic chemical route to compound **25b**.

*2-Methyl-3-(3-bromo-benzyl)-4a,8a-dihydro-[1,4]naphthoquinone* (**25b**). After chromatography on silica gel (petroleum ether/dichloromethane 1:1), 328 mg (9.96 mmol, 52% yield) of **25b** (Scheme 19) were isolated as a yellow solid. M.p. 108–109 °C. ^1^H-NMR (300 MHz, CDCl_3_): δ = 7.98–8.02 (m, 2H), 7.59–7.65 (m, 2H), 7.28 (s, 1H), 7.24 (td, ^3^*J* = 6.78 Hz, ^4^*J* = 1.97 Hz, 1H), 7.02–7.09 (m, 2H), 3.92 (s, 2H), 2.16 (s, 3H). ^13^C-NMR (75 MHz, CDCl_3_): δ = 185.16 (Cq), 184.45 (Cq), 144.78 (Cq), 144.50 (Cq), 140.35 (Cq), 133.59 (CH), 132.10 (Cq), 131.94 (Cq), 131.52 (CH), 130.16 (CH), 129.65 (CH), 127.26 (CH), 126.54 (CH), 126.36 (CH), 122.72 (Cq), 32.09 (CH_2_), 13.35 (CH_3_). EI MS (70 eV, *m/z* (%)): 340.0 ([M]^+^, 28), 325.06 (100), 246.13 (18), 215.14 (7), 202.12 (8), 184.99 (12), 76.0 (10). IR (KBr): 3430 cm^−1^ (b, w), 1658 (vs), 1620 (vs), 1595 (vs), 1568 (s), 1474 (s), 1431 (m), 1381 (s), 1334 (vs), 1290 (vs), 1261 (s), 1180 (s), 1074 (m), 974 (s), 955 (s), 793 (s), 780 (vs), 728 (vs), 692 (s), 687 (s), 422 (m). EA: obs. C, 63.55%; H, 3.94%; Br, 23.69%, calcd. C, 63.36%; H, 3.84%; Br, 23.42% for C_18_H_13_BrO_2_.

### 4.5. Deprotection of the Benzylamine Acteyl Group (Synthesis of ***7*** and ***17***)

**Scheme 20:**
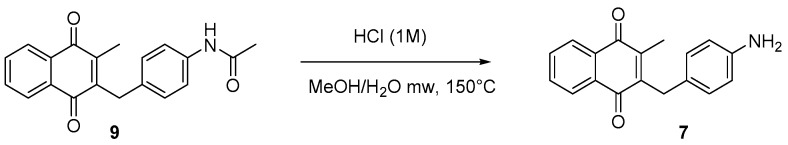
Synthetic chemical route to compound **7**.

*2-(4-Aminobenzyl)-3-methylnaphthalene-1,4-dione* (**7**)*.* 4-Acetamide derivative **9** (200 mg, 0.62 mmol) was added to HCl (1 mL) in a mixture of water and methanol (6 mL, *v/v*, 1/1) under microwave irradiation (150 °C) for 2 h. The reaction mixture was poured into ethyl acetate then washed with a saturated Na_2_CO_3_ solution and then allowed to dry. 172 mg (76%) of compound **7** (Scheme 20) was isolated as a purple solid with no further purification. m.p. 162–164 °C. ^1^H-NMR (300 MHz, CDCl_3_): δ = 8.09–8.06 (m, 2H), 7.69–7.67 (m, 2H), 7.03 (d, *J* = 8.30 Hz, 2H), 6.60 (d, *J* = 8.27 Hz, 2H), 3.91 (s, 2H), 2.24 (s, 3H). ^13^C-NMR (100 MHz, CDCl_3_): δ = 185.68 (C_q_), 184.93 (C_q_), 145.99 (C_q_), 144.95 (C_q_), 143.97 (C_q_), 133.53 (CH), 133.49 (CH), 132.27 (C_q_), 132.25 (C_q_), 129.67 (CH), 127.97 (C_q_), 126.54 (CH), 126.33 (CH), 115.50 (CH), 31.69 (CH_2_), 13.33 (CH_3_). IR: 3474 (w), 3377 (w), 2932 (w), 1653 (s), 1617 (s), 1590 (m), 1515 (s), 1336 (m), 1293 (vs), 1182 (m), 973 (m) 816 (m), 768 (m), 708 (vs). HRMS-ESI (*m/z*): [M + H]^+^ calcd. for C_18_H_16_NO_2_ 278.1176; found 278.1154.

**Scheme 21:**
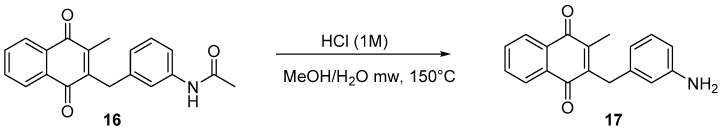
Synthetic chemical route to compound **17**.

*2-(3-Aminobenzyl)-3-methylnaphthalene-1,4-dione* (**17**)*.* 3-Acetamide derivative **16** (278 mg, 0.87 mmol) was added to HCl (1 mL) in a mixture of water and methanol (8 mL, *v/v*, 1/1) under microwave irradiation (150 °C) for 20 min. The reaction mixture was poured into ethyl acetate then washed with a saturated Na_2_CO_3_ solution and then allowed to dry. After chromatography on silica gel (cyclohexane/ethyl acetate 75:25 to cyclohexane/ethyl acetate 1:1), 150 mg (0.54 mmol, 62% yield) of compound **17** (Scheme 21) was isolated as a purple solid. m.p. 159–160 °C. ^1^H-NMR (300 MHz, CDCl_3_): δ = 8.12–8.06 (m, 2H), 7.73–7.67 (m, 2H), 7.05 (t, ^3^*J* = 7.67 Hz, 1H), 6.62 (d, ^3^*J* = 7.56 Hz, 1H), 6.56–6.48 (m, 2H), 3.95 (s, 3H), 2.24 (s, 3H). ^13^C-NMR (75 MHz, CDCl_3_): δ = 185.67 (C_q_), 184.95 (C_q_), 146.88 (C_q_), 145.63 (C_q_), 144.66 (C_q_), 139.46 (C_q_), 133.69 (CH), 133.37 (CH), 132.40 (C_q_), 132.32 (CH), 129.75 (CH), 126.72 (CH), 126.50 (CH), 119.19 (CH), 115.49 (CH), 113.54 (CH), 32.56 (CH_2_), 13.52 (CH_3_); ESI MS: 278.12 (M^+^). IR: 3452 cm^−1^ (w), 3372 (w), 2921 (w), 1649 (m), 1615 (s), 1588 (s), 1492 (m), 1335 (m), 1292 (vs), 974 (m), 867 (m), 716 (s).

### 4.6. Dialkylation of Benzylamine Menadione Derivatives (Synthesis of ***3**, **18**, **11*** and ***19***)

**Scheme 22:**
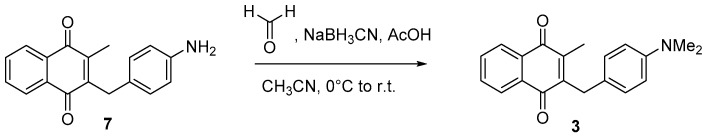
Synthetic chemical route to compound **3**.

*2-(4-(Dimethylamino)benzyl)-3-methylnaphthalene-1,4-dione* (**3**). Sodium cyanoborohydride (52 mg, 0.82 mmol, 3 equiv.) was added by small portions to a stirred solution of **7** (76 mg, 0.27 mmol, 1 equiv.) in acetonitrile (5 mL) and 37% aqueous formaldehyde (220 µL, 2.7 mmol, 10 equiv.) in acetonitrile (1 mL) placed in a cool bath was then added. An additional glacial acetic acid portion (45 µL, 0.46 mmol, 1.7 equiv.) was added and stirring for 2 h. The reaction mixture was poured into diethyl ether (10 mL) and then washed with 1N potassium hydroxide (10 mL) and brine. The organic phase was dried and evaporated in vacuo. The crude product was purified by chromatography on silica gel using a mixture of cyclohexane/ethyl acetate (10:1 to 5:5) affording the compound **3** (Scheme 22; 48 mg, 59%) as a purple solid. ^1^H-NMR (300 MHz, CDCl_3_): δ = 8.09–8.06 (m, 2H), 7.71–7.67 (m, 2H), 7.12 (d, ^3^*J* = 8.78 Hz, 2H), 6.67 (d, ^3^*J* = 7.73 Hz, 2H), 3.93 (s, 2H), 2.89 (s, 6H), 2.27 (s, 3H). ^13^C-NMR (75 MHz, CDCl_3_): δ = 185.72 (C_q_), 185.00 (C_q_), 145.96 (C_q_), 143.65 (C_q_), 142.94 (C_q_), 133.51 (CH), 133.47 (CH), 132.28 (C_q_), 129.52 (CH), 126.55 (CH), 126.31 (CH), 113.26 (C_q_), 40.96 (CH_3_), 31.56 (CH_2_), 13.35 (CH_3_). IR: 2923 cm^−1^ (w), 2852 (w), 1660 (s), 1615 (m), 1594 (m), 1520 (s), 1291 (vs), 1130 (m), 945 (m), 814 (m), 689 (s).

**Scheme 23:**
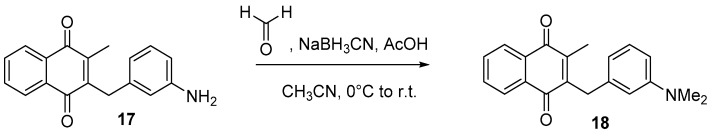
Synthetic chemical route to compound **18**.

*2-(3-(Dimethylamino)benzyl)-3-methylnaphthalene-1,4-dione* (**18**)*.* Sodium cyanoborohydride (52 mg, 0.82 mmol, 4.5 equiv.) was added by small portions to a stirred solution of **17** (50 mg, 0.18 mmol, 1 equiv.) in acetonitrile (5 mL) and 37% aqueous formaldehyde (220 µL, 2.7 mmol, 15 equiv.) in acetonitrile (1 mL) was then added. An additional glacial acetic acid portion (45 µL, 0.46 mmol, 5.1 equiv.) was added and stirring for 2 h. The reaction mixture was poured into diethyl ether (10 mL) and then washed with potassium hydroxide 1 N (10 mL) and brine. The organic phase was dried and evaporated in vacuo. The crude product was purified by chromatography on silica gel using a mixture of cyclohexane/ethyl acetate (10:1 to 5:5) affording the compound **18** (Scheme 23; 17 mg, 31%) as a purple solid. ^1^H-NMR (300 MHz, CDCl_3_): δ = 8.12–8.06 (m, 2H), 7.72–7.66 (m, 2H), 7.12 (t, ^3^*J* = 7.89 Hz, 1H), 6.65 (sl, 1H), 6.60–6.55 (m, 2H), 4.00 (s, 2H), 2.91 (s, 6H), 2.27 (s, 3H). ^13^C-NMR (100 MHz, CDCl_3_): δ = 185.58 (C_q_), 184.82 (C_q_), 150.93 (C_q_), 145.74 (C_q_), 144.35 (C_q_), 138.95 (C_q_), 133.51 (CH), 133.46 (CH), 132.26 (C_q_), 132.25 (C_q_), 129.37 (CH), 126.56 (CH), 126.31 (CH), 116.97 (CH), 113.17 (CH), 110.94 (CH), 40.69 (CH_3_), 32.90 (CH_2_), 13.42 (CH_3_). IR: 2920 cm^−1^ (w), 2807 (w), 1661 (vs), 1594 (vs), 1501 (m), 1361 (m), 1292 (m), 1292 (vs), 997 (m), 974 (m), 842 (m), 758 (s), 744 (s), 691 (vs).

**Scheme 24:**
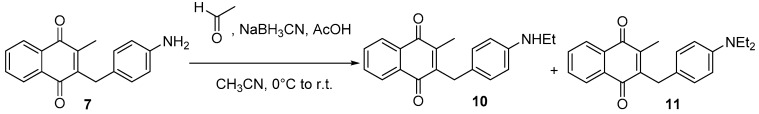
Synthetic chemical route to compound **11**.

*2-(4-(Diethylamino)benzyl)-3-methylnaphthalene-1,4-dione* (**11**)*.* Sodium cyanoborohydride (67 mg, 1.08 mmol, 3 equiv.) was added by small portions to a stirred solution of **7** (100 mg, 0.36 mmol, 1 equiv.) and acetaldehyde (200 µL, 3.6 mmol, 10 equiv.) in acetonitrile (1 mL) placed in a cool bath was added. Glacial acetic acid (60 µL, 0.61 mmol, 1.7 equiv.) was then added and stirring for 2 h. The reaction mixture was poured into diethyl ether (10 mL) and then washed with potassium hydroxide 1N (10 mL) and brine. The organic phase was dried and evaporated in vacuo. The crude product was purified by chromatography on silica gel using a mixture of cyclohexane/ethyl acetate (10:1 to 5:5) affording the compounds **10** (17%) and **11** (Scheme 24; 36%) as a purple solid. m.p. 94–96 °C. ^1^H-NMR (300 MHz, CDCl_3_): δ = 8.11–8.04 (m, 2H), 7.71–7.65 (m, 2H), 7.08 (d, ^3^*J* = 8.70 Hz, 2H), 6.58 (d, ^3^*J* = 8.70 Hz, 2H), 3.91 (s, 2H), 3.29 (q, ^3^*J* = 7.06 Hz, 4H), 2.28 (s, 3H), 1.11 (t, ^3^*J* = 7.06 Hz, 6H); ^13^C-NMR (75 MHz, CDCl_3_): δ = 185.81 (C_q_), 185.02 (C_q_), 146.60 (C_q_), 146.21 (C_q_), 143.69 (C_q_), 133.48 (C_q_), 133.43 (CH), 132.32 (CH), 129.73 (C_q_), 129.62 (CH), 126.55 (CH), 126.30 (CH), 124.56 (C_q_), 112.25 (CH), 44.48 (CH_2_), 31.50 (CH_2_), 13.37 (CH_3_), 12.71 (CH_3_). ESI MS: 333.17 (M^+^); EA: obs. C, 80.19%; H, 8.01%; N, 5.35%, calcd. C, 79.25%; H, 6.25%, N, 4.20% for C_22_H_23_NO_2_; HRMS-ESI (*m/z*): [M + H]^+^ calcd. for C_22_H_24_NO_2_ 334.1802; found 334.1765.

**Scheme 25:**
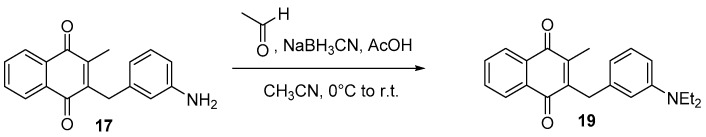
Synthetic chemical route to compound **19**.

*2-(3-(Diethylamino)benzyl)-3-methylnaphthalene-1,4-dione* (**19**). Sodium cyanoborohydride (52 mg, 0.82 mmol, 4.5 equiv.) was added by small portions to a stirred solution of **17** (50 mg, 0.18 mmol, 1 equiv.) and acetaldehyde (150 µL, 2.7 mmol, 15 equiv.) in acetonitrile (1 mL) placed in a cool bath was added. Glacial acetic acid (45 µL, 0.46 mmol, 5.1 equiv.) was then added and stirring for 2 h. The reaction mixture was poured into diethyl ether (10 mL) and then washed with 1N potassium hydroxide (10 mL) and brine. The organic phase was dried and evaporated in vacuo. The crude product was purified by chromatography on silica gel using a mixture of cyclohexane/ethyl acetate (10:1 to 5:5) to afford the compound **19** (Scheme 25; 13 mg, 22%) as a purple solid. ^1^H-NMR (300 MHz, CDCl_3_): δ = 8.12–8.05 (m, 2H), 7.72–7.66 (m, 2H), 7.09 (t, ^3^*J* = 7.92 Hz, 1H), 6.60 (sl, 1H), 6.53–6.47 (m, 2H), 3.99 (s, 2H), 3.31 (q, ^3^*J* = 7.06 Hz, 4H), 2.29 (s, 3H), 1.13 (t, ^3^*J* = 7.03 Hz, 6H). ^13^C-NMR (75 MHz, CDCl_3_): δ = 185.66 (C_q_), 184.87 (C_q_), 148.13 (C_q_), 145.88 (C_q_), 144.16 (C_q_), 139.07 (C_q_), 133.52 (CH), 133.45 (CH), 132.27 (C_q_), 132.24 (C_q_), 129.57 (CH), 126.54 (CH), 126.31 (CH), 115.67 (CH), 112.40 (CH), 110.10 (CH), 44.47 (CH_2_), 32.98 (CH_2_), 13.45 (CH_3_), 12.70 (CH_3_). IR: 2975 cm^−1^ (w), 2929 (w), 1658 (s), 1595 (vs), 1498 (m), 1290 (vs), 1023 (m), 975 (m), 755 (m), 731 (s), 693 (s). HRMS-ESI (*m/z*): [M + H]^+^ calcd. for C_22_H_24_NO_2_ 334.1802; found 334.1819.

### 4.7. General Procedure for the Suzuki-Miyaura Coupling Reaction (Synthesis of ***26**–**28***)

A Schlenk-tube was flushed with argon and successively filled with **25** (100 mg, 0.29 mmol, 1 equiv.), boronic acid derivative (0.32 mmol, 1.1 equiv.), K_2_CO_3_ (122 mg, 0.88 mmol, 3.0 equiv.) and finally dissolved in dioxane/water (12 mL/3 mL). The solution was degassed by bubbling argon through the mixture for 20 min. Then 4 mol % PdCl_2_(dppf) (10 mg, 0.012 mmol) was added, the Schlenk-tube was sealed and heated overnight at 80 °C. The reaction was quenched by adding 10 mL H_2_O. All volatiles solvents were removed in vacuo, the product was extracted with CH_2_Cl_2_, dried over MgSO_4_ and purified by flash-chromatography.

**Scheme 26:**
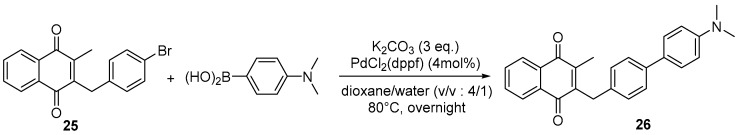
Synthetic chemical route to compound **26**.

*2-(4′-Dimethylamino-biphenyl-4-ylmethyl)-3-methyl-[1,4]naphthoquinone* (**26**). 4-Dimethylamino phenylboronic acid was used as the boronic acid coupling partner for compound **25**. After chromatography on silica gel (dichloromethane:petroleum ether/3:1), 96 mg (0.25 mmol, 86% yield) of **26** (Scheme 26) was isolated as a black solid. m.p. 170–171 °C. ^1^H-NMR (300 MHz, CDCl_3_): δ = 8.05–8.11 (m, 2H), 7.65–7.71 (m, 2H), 7.43 (d, ^3^*J* = 8.39 Hz, 4H), 7.24 (d, ^3^*J* = 8.17 Hz, 2H), 6.76 (d, ^3^*J* = 8.09 Hz, 2H), 4.03 (s, 2H), 2.96 (s, 6H), 2.27 (s, 3H). ^13^C-NMR (75 MHz, CDCl_3_): δ = 185.47 (C_q_), 184.74 (C_q_), 145.43 (C_q_), 144.34 (C_q_), 139.43 (C_q_), 135.69 (C_q_), 133.49 (CH), 133.46 (CH), 132.15 (C_q_), 132.09 (C_q_), 128.94 (CH), 127.58 (CH), 126.53 (CH), 126.52 (CH), 126.28 (CH), 112.75 (CH), 40.60 (CH_3_), 32.08 (CH_2_), 13.37 (CH_3_). EI MS (70 eV, *m/z* (%)): 381.2 ([M]^+^, 100), 366.1 (19). IR (KBr): 3432 cm^−1^ (b, m), 3028 (w), 2922 (w), 2855 (w), 2803 (w), 1660 (vs), 1612 (vs), 1595 (s), 1534 (w), 1504 (vs), 1444 (w), 1375 (w), 1357 (m), 1333 (m), 1294 (vs), 1226 (w), 1168 (w), 946 (w), 810 (s), 790 (s), 766 (w), 722 (w), 711 (w), 692 (w). EA: obs. C, 81.58%; H, 619%; N, 3.60%, calcd. C, 81.86%; H, 6.08%; N, 3.67% for C_26_H_23_NO_2_.

**Scheme 27:**
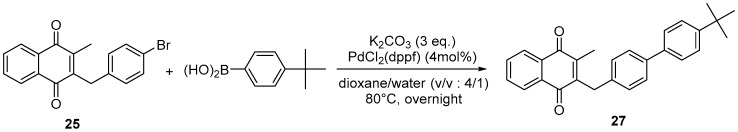
Synthetic chemical route to compound **27**.

*2-(4′-tert-Butyl-biphenyl-4-ylmethyl)-3-methyl-[1,4]naphthoquinone* (**27**)*. tert*-Butylphenylboronic acid was used as the boronic acid coupling partner for compound **25**. After chromatography on silica gel (dichloromethane:petroleum ether/3:1), 113 mg (0.286 mmol, 97% yield) of **27** (Scheme 27) was isolated as a yellow solid. M.p. 112–114 °C. ^1^H-NMR (300 MHz, CDCl_3_): δ = 8.06–8.13 (m, 2H), 7.66–7.71 (m, 2H), 7.41–7.50 (m, 6H), 7.24–7.30 (m, 2H), 4.06 (s, 2H), 2.28 (s, 3H), 1.35 (s, 9H). ^13^C-NMR (75 MHz, CDCl_3_): δ = 185.34 (C_q_), 184.63 (C_q_), 150.13 (C_q_), 145.25 (C_q_), 144.40 (C_q_), 139.24 (C_q_), 137.85 (C_q_), 136.75 (C_q_), 133.45 (CH), 132.12 (C_q_), 132.03 (C_q_), 128.93 (CH), 127.21 (CH), 126.59 (CH), 126.46 (CH), 126.25 (CH), 125.64 (CH), 34.47 (C_q_), 32.08 (CH_2_), 31.32 (CH_3_), 13.30 (CH_3_). EI MS (70 eV, *m/z* (%)): 394.3 ([M]^+^, 42), 379.2 (100). IR: 3439 cm^−1^ (b, m), 2962 (w), 1659 (vs), 1620 (m), 1595 (m), 1498 (w), 1462 (w), 1377 (w), 1334 (w), 1295 (s), 1182 (w), 1114 (w), 976 (w), 815 (m), 790 (w), 713 (w), 568 (w). EA: obs. C, 85.16%; H, 6.66%, calcd. C, 85.25%; H, 6.64% for C_28_H_26_O_2_.

**Scheme 28:**
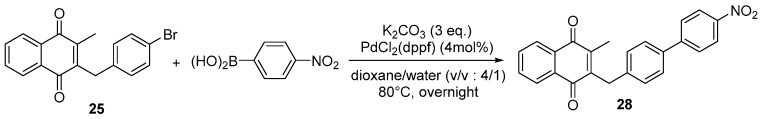
Synthetic chemical route to compound **28**.

*2-(4′-nitro-Butyl-biphenyl-4-ylmethyl)-3-methyl-[1,4]naphthoquinone* (**28**)*.* 4-Nitrophenylboronic acid was used as the boronic acid coupling partner for compound **25**. After chromatography on silica gel (dichloromethane:petroleum ether/3:1), 67 mg (0.175 mmol, 95% yield) of **28** (Scheme 28) was isolated as a yellow solid. M.p. 197–199 °C. ^1^H-NMR (300 MHz, CDCl_3_): δ = 8.23–8.26 (m, 2H), 8.05–8.11 (m, 2H), 7.65–7.73 (m, 4H), 7.51 (d, ^3^*J* = 8.27 Hz, 2H), 7.34 (d, ^3^*J* = 8.22 Hz, 2H), 4.08 (s, 2H), 2.27 (s, 3H). ^13^C-NMR (75 MHz, CDCl_3_): δ = 185.27 (C_q_), 184.65 (C_q_), 147.18 (C_q_), 147.00 (C_q_), 144.86 (C_q_), 144.68 (C_q_), 139.16 (C_q_), 136.95 (C_q_), 133.63 (CH), 132.12 (C_q_), 131.98 (C_q_), 129.40 (CH), 127.65 (CH), 127.59 (CH), 126.52 (CH), 126.38 (CH), 124.12 (CH), 32.22 (CH_2_), 13.40 (CH_3_). EI MS (70 eV, *m/z* (%)): 383.3 ([M]^+^, 41), 368.2 (100). IR (KBr): 3436 cm^−1^ (b, m), 3073 (w), 2934 (w), 1661 (vs), 1620 (m), 1596 (vs), 1513 (vs), 1485 (m), 1375 (w), 1344 (vs), 1295 (vs), 1261 (w), 1182 (w), 1111 (m), 974 (w), 852 (m), 821 (m), 787 (w), 745 (m), 711 (m), 693 (m), 555 (w). EA: obs. C, 72.95%; H, 4.47%; N, 3.56%, calcd. C, 72.79%; H, 4.68%; N, 3.54% for C_24_H_17_NO_4_ 0.7 H_2_O.

### 4.8. *In Vitro* Antimalarial Activity

#### 4.8.1. Hypoxanthine Method Determination of IC_50_ Values of Dd2 Growth Inhibition

The IC_50_ values were determined using standard in vitro proliferation assays [58]. Erythrocytes infected with ring-stage parasites (0.5% parasitemia, 2.5% hematocrit) were exposed to the compounds for 48 h and then to radioactive hypoxanthine for 24 h in 96-well plates. The amount of radioactivity in precipitable material served as an index of parasite proliferation.

#### 4.8.2. SYBR_green_ Method for Determination of IC_50_ Values of *P. falciparum* Dd2

In vitro antiplasmodial activity is expressed as the 50% inhibitory concentration (IC_50_) of intraerythrocytic parasite development, using the SYBR_green_ I assay as described before [5,59]. Briefly, synchronous ring-stage parasites were incubated for 72 h in the presence of decreasing drug concentrations in microtiter plates (final parasitemia 0.5%; final hematocrit 1.5%). Each inhibitor was analyzed in 3-fold serial dilutions in duplicate and in at least three independent experiments. Parasite replication was assessed by fluorescent SYBR_green_ staining of parasitic DNA [60] as previously described [59]. IC_50_ values were calculated using SigmaPlot.

### 4.9. Cytotoxicity against Human MRC-5 Cells

MRC-5_SV2_ cells are cultured in Earls MEM +5% FCSi. Assays are performed in 96-well microtiter plates, each well containing about 10^4^ cells/well. After 3 days incubation, cell viability is assessed fluorimetrically after addition of resazurin and fluorescence is measured (λ_ex_ 550 nm, λ_em_ 590 nm). The results are expressed as % reduction in cell growth/viability compared to untreated control wells and CC50 is determined. Compounds are tested at 5 concentrations (64–16–4–1–0.25 µM). When the CC_50_ is lower than 16 µM, the compound is classified as toxic.

### 4.10. In Vivo Antimalarial Activity

#### 4.10.1. Animals

Swiss mice (female—BW ~ 25 g; Janvier France, Le Genest Saint Isle, France) were allocated randomly to five groups of four animals each. Drinking water and food were available *ad libitum* throughout the experiment. The weight of the individual animals did not differ too much from the group mean.

#### 4.10.2. Infection and Drug Treatment of *P. berghei*-Infected Mice

*P. berghei* (ANKA-strain) is maintained in the laboratory by weekly mechanical subpassage in Swiss mice. The infection inoculum was prepared by taking heparinized blood was collected from a clinically ill donor mouse (approximately 20% parasitaemia) and diluted in PBS to obtain an infection inoculum of 0.15 mL with about 4 × 10^8^ infected erythrocytes. The infection-inoculum was given intraperitoneally. The untreated infected controls developed severe malaria with most animals showing severe clinical signs on day 4. Chloroquine was used as reference treatment (10 mg/kg ip for 5 days). Treatment with chloroquine resulted in 100% survival until day 7 with low parasitaemia at day 4 post infection. The tested compounds were evaluated after ip dosing at 50 mg/kg for 5 days.

### 4.11. Physicochemistry

#### 4.11.1. Starting Materials and Solvents

Distilled water was further purified by passing it through a mixed bed of ion-exchanger (R3-83002, M3-83006, Bioblock Scientific, Illkirch, France) and activated carbon (Bioblock Scientific ORC-83005) and was de-oxygenated by CO_2_- and O_2_-free argon (Sigma Oxiclear cartridge) before use. All the stock solutions were prepared in spectroscopic grade dimethylsulfoxide (DMSO, Bioreagent for Molecular Biology, >>99.9%, Sigma) by weighing solid products using a XA105 Dual Range (0.01/0.1 mg–41/120 g) balance Mettler Toledo (Viroflay, France) and the complete dissolution was achieved using an ultrasonic bath. The ionic strength was maintained at 0.1 M with sodium perchlorate (NaClO_4_.H_2_O, Merck, p.a.), and all measurements were carried out at 25.0 ± 0.2 °C. *CAUTION! Perchlorate salts combined with organic ligands are potentially explosive and should be handled in small quantities and with the adequate precautions* [61].

#### 4.11.2. Spectrophotometric Titrations Versus pH

The acido-basic properties of plasmodione derivatives **7** and **17** were performed in water (0.1 M NaClO_4_) while those of compounds **3**, **10**, **11**, **18** and **19** were performed in NaClO_4_ 0.1 M/DMSO (91:9 *v/v*) for solubility reasons*.* Absorption spectrophotometric titrations versus pH titrations of plasmodione derivatives **3** (1.81 × 10^−5^ M), **7** (2.00 × 10^−5^ M), **10** (1.81 × 10^−5^ M), **11** (2.18 × 10^−5^ M), **17** (2.00 × 10^−5^ M), **18** (1.84 × 10^−5^ M) and **19** (1.79 × 10^−5^ M) were carried out by diluting stock solutions freshly prepared in spectroscopic grade DMSO in 40 mL of the corresponding solvent contained in a jacketed cell (Metrohm, Villebon sur Yvette, France) maintained at 25.0 ± 0.2 °C (thermostat E200, Lauda, Roissy Charles de Gaulle, France). The free hydrogen ion concentration was measured with a combined glass electrode (Metrohm 6.0234.500, Long Life) filled with 0.1 M NaCl in water and an automatic titrator system 794 Basic Titrino (Metrohm) connected to a microcomputer (Tiamo light 1.2 program for the acquisition of the potentiometric data). The combined glass electrode was calibrated as a hydrogen concentration probe by titrating known amounts of hydrochloric acid (~1.06 × 10^−1^ M from HClO_4_, normapur, 70% min Prolabo) with CO_2_-free sodium hydroxide solution (~9.35 × 10^−2^ M from NaOH, BdH Prolabo Chemicals, AnalaR, via VWR distribution) [62]. The HClO_4_ and NaOH solutions were freshly prepared just before use and titrated with sodium tetraborate decahydrate (B_4_Na_2_O_7_·10H_2_O, puriss, p.a, Fluka) and potassium hydrogen phthalate (C_8_H_5_KO_3_, Fluka, puriss, p.a.), respectively, using methyl orange (RAL) and phenolphthalein (Prolabo, purum) as the indicators. The GLEE program [62] was applied for the glass electrode calibration (standard electrode potential E_0_/mV and slope of the electrode/mV pH^−1^) and to check carbonate levels of the NaOH solutions used (<5%). The cell was thermostated at 25.0 ± 0.2 °C by the flow of a Lauda E200 thermostat. A stream of argon, pre-saturated with water vapour, was passed over the surface of the solution. The initial pH was adjusted to ~2–3 with HClO_4_ (Prolabo, normapur, 70% min) and the absorption spectrophotometric titrations of the plasmodione derivatives versus pH (~3 < pH < ~9–10) were carried out by addition of known volumes of NaOH solutions using the automatic titrator of the 794 Basic Titrino device (DET method). After each addition, an absorption spectra was automatically and repeatedly recorded using a CARY 50 spectrophotometer (Varian, Les Ulis, France) fitted with Hellma optical fibers (041.002-UV, Hellma, Plainview, NY, USA) and an immersion probe made of quartz suprazil (Hellma, 661.500-QX) and interfaced (Cetrib) with the potentiometric unit. The distribution curves of the protonated species of the plasmodione derivatives as a function of pH were calculated using the Hyss program [63].

#### 4.11.3. Analysis and Processing of the Spectroscopic Data

The spectrophotometric data were analyzed with the Specfit program [64,65,66] which adjusts the absorptivities and the stability constants of the species formed at equilibrium. Specfit uses factor analysis to reduce the absorbance matrix and to extract the eigenvalues prior to the multiwavelength fit of the reduced data set according to the Marquardt algorithm [67,68].

## 5. Conclusions

In summary, new synthetic procedures were successfully developed for the preparation of biaryl analogues of the antimalarial plasmodione in a 2-step sequence based on Kochi-Anderson and Suzuki-Miyaura coupling reactions. 3-Benzylmenadione derivatives bearing a terminal -*N*(Me)_2_ or -*N*(Et)_2_ in the different position of the benzylic chain were prepared in a 4-step synthesis for antimalarial drug evaluation using the multiresistant *P. falciparum* Dd2 strain. Interestingly, the non-protonable 4′-*N*HBoc-amino-3-benzylmenadione was selected for its potent in vitro activity, suggesting that cytosol-food vacuole shuttling of redox-cyclers may not need to accumulate in the food vacuole through lysosomotropic effects. This compound also revealed to be active in vivo in *P. berghei*-infected mice. These observations encourage pursuing further optimization of 3-benzyl-menadiones to improve the pharmacokinetic profile of plasmodione that has been shown to fulfill the criteria required for early antimalarial lead compounds.

## Figures and Tables

**Table 1:** Mean IC_50_ values (nM) for plasmodione derivatives determined with the tritiated hypoxanthine incorporation method against *P. falciparum* Dd2 ^a^, and with the Alamar blue method against the human MRC-5 fibroblasts.

Compound Code	Structure	IC_50_ (nM)	Tox/MRC-5 (µM)
Plasmodione ^b^	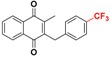	29 ± 2 (3)	>32.0
**6**	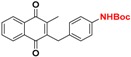	42.5 (2)	>64.0
**25c** ^b^	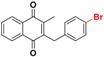	46 ± 4 (5)	>32.0
**26**	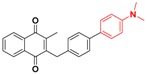	223	>64.0
**27**	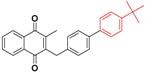	485	>64.0
**28**	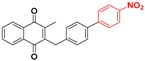	474	>64.0
Chloroquine ^b^	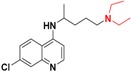	110 ± 20 (4)	51.5

^a^ In vitro activity against *P. falciparum* Dd2 strain is presented as mean IC_50_ values of benzylmenadione derivatives using the tritiated hypoxanthine incorporation method; ^b^ Chloroquine and plasmodione were used as standard drugs. The listed IC_50_ values of chloroquine, plasmodione and its bromo analogue **25c** (named benzylMD **1c** and **1a** in [3]) are previously reported values.

**Table 2:** Mean IC_50_ values (nM) for plasmodione derivatives determined in the SYBR_green_ method with *P. falciparum* Dd2.

Compound Code	Structure	IC_50_ (nM) ± SD (n) ^a^	Tox/MRC-5 (µM)
**3**	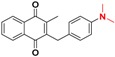	567 ± 217(3)	>64.0
**6**	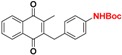	109 ± 46 (3)	>64.0
**7**	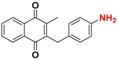	303 ± 138 (3)	>64.0
**9**	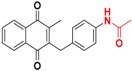	411 ± 220 (3)	>64.0
**10**	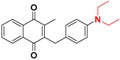	418 ± 166 (3)	>64.0
**11**	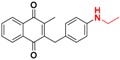	2056 ± 1073 (3)	29.3
**16**	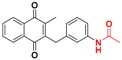	310 ± 134 (3)	4.5
17 *	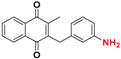	1198 ± 1651 (3)	>64.0
**18**	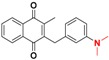	386 ± 151 (3)	>64.0
**19**	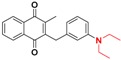	236 ± 97 (3)	>64.0
**22**	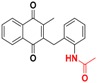	282 ± 86 (3)	4.4
**25a**	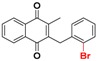	86.3 (1)	>64.0
**25b**	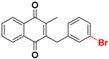	127.6 (1)	20.8
**25c**	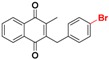	82 ± 2 (7)	>32.0 ^b^
Plasmodione	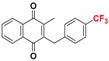	58 ± 11 (9)	>32.0 ^b^
Chloroquine	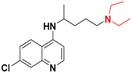	99 ± 19 (6)	51.5 ^b^

^a^ In vitro activity against *P. falciparum* Dd2 is presented as mean IC_50_ values ± standard deviation (SD) determined from (n) independent growth inhibition assays in triplicate using the SYBR_green_^®^ technique; ^b^ Chloroquine was used as reference drug. IC_50_ values of chloroquine, plasmodione and its bromo analogue **25c** (named benzylMD **1c** and **1a** in [3]) are previously reported values. * Precipitates in DMSO.

**Table 3:** In vivo antimalarial activity in *P. berghei* ANKA-infected Swiss mice after a 5-day intraperitoneal dosing.

Compound	Treatment (ip for 5 Days)	Animals (n)	% Parasitaemia (dpi)				% Suppression (dpi)				Health Status
			4	7	11	14	4	7	11	14	
Untreated control	-	5	38.3	42.2	67.7	61.9	-	-	-	-	From day 4 post-infection (p.i.) onwards:poor appearance, tremor, body weight loss, rough hair, 4 animals died before day 7 p.i.
Chloroquine	10 mg/kg	5	5.2	9.8	32.1	56.2	86.3	76.8	52.7	9.2	From day 4 p.i. onwards: poor appearance, tremor, body weight loss, rough hair, one animal died before day 14 p.i.
Compound 6	50 mg/kg	4	9.4	7.7	41.9	69.3	75.5	81.8	38.1	12.0	From day 4 p.i. onwards: poor appearance (intermittent), one animal died before day 14 p.i.

**Table 4:** p*K*_a_ values for selected plasmodione derivatives ^a^.

Compound Code	Structure	p*K*_a_ ± σ [LH^+^]_pH5_/[LH^+^]_pH7.4_
**3**	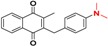	4.87 ± 0.03 144.7
**7** ^b^	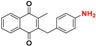	4.74 ± 0.05 162.5
**10**	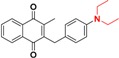	^e^
**11**	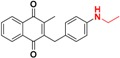	5.07 ± 0.06 112.0
**17** *^,b^	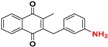	4.30 ± 0.02 209.6
**18**	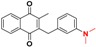	4.60 ± 0.04 179.9
**19**	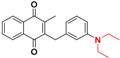	^e^
Methylene blue ^c^	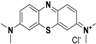	p*K*_a1_ = 1.7 p*K*_a2_ = 4.5 p*K*_a3_ = 5.9
Chloroquine ^d^	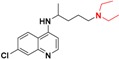	p*K*_a1_ = 8.4 ± 0.2 p*K*_a2_ = 10.6 ± 0.2 1.1

^a^ Water/DMSO (0.91/0.09 *v/v*); I = 0.1 M NaClO_4_; T = 25 °C; ^b^ Water; I = 0.1 M NaClO_4_; T = 25 °C; ^c^ p*K*_a_ values were reported [4,29]; ^d^ p*K*_a_ values were measured in [56]; ^e^ Precipitation prevented the accurate determination of the p*K*_a_ values which were estimated to be ~5–5.5. Error = 1σ with σ = standard deviation. * Precipitates in DMSO.

## Supplementary Materials

Supplementary materials can be accessed at: http://www.mdpi.com/1420-3049/22/1/161/s1., on pages S2-S5: Physico-chemical data for compounds **7**, **11**, **17**, **18** (Figures S1–S4), and pages S6–S31: ^1^H-NMR and ^13^C-NMR spectra of compounds **3**, **5**–**9**, **11**, **14**–**19**, **21**–**22**, **25**–**28**.

## Abbreviations

The following abbreviations are used in this manuscript:

ACT	Artemisinin-based Combination Therapy
CQ	chloroquine
DHODH	dihydroorotate dehydrogenase
DMSO	dimethylsulfoxide
EA	Elemental analyses
equiv.	equivalent
ESI-MS	Electronspray-Mass spectrometry
HRMS	High Resolution Mass Spectra
ip	intraperitoneal
MB	methylene blue
mETC	mitochondrial electron transport
M.p.	Melting point
*N-*Boc	*N-tert-*butyloxycarbonyl
yDHODH	dihydroorotate dehydrogenase from yeast
