# Supplementary material for: Pharmacomodulation of the Antimalarial Plasmodione: Synthesis of Biaryl- and N-Arylalkylamine Analogues, Antimalarial Activities and Physicochemical Properties
Source: Molecules. 2017 Jan 19;22(1):161. doi: 10.3390/molecules22010161 (PMC6155649; doi:10.3390/molecules22010161)
Supplement: Supplementary file 1 [file molecules-22-00161-s001.pdf]

Supplementary materials: Pharmacomodulation of the Antimalarial Plasmodione: Synthesis of Biaryl- and *N*-Arylalkylamine Analogues, Antimalarial Activities and Physicochemical Properties

Karène Urgin, Mouhamad Jida, Katharina Ehrhardt, Tobias Müller, Michael Lanzer, Louis Maes, Mourad Elhabiri, and Elisabeth Davioud-Charvet

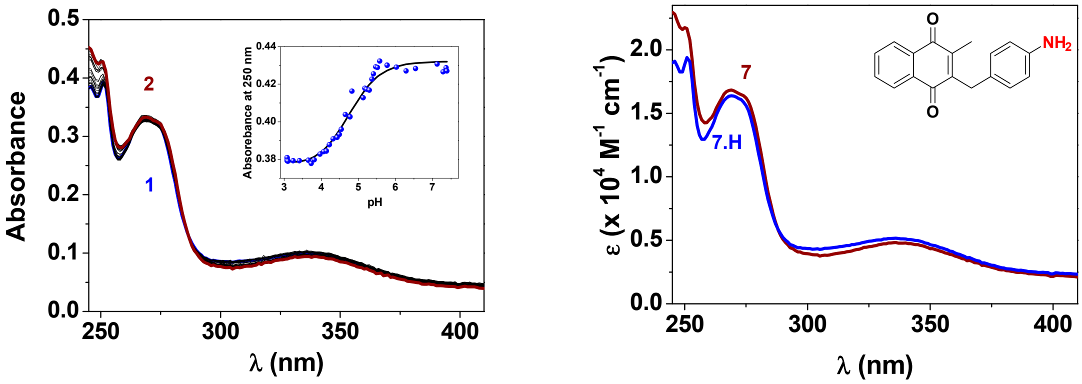

(**A**) (**B**)

Figure S1. (**A**) Absorption spectrophotometric titration of compound 7 as a function of pH and (**B**) electronic spectra of 7 and its protonated species 7.H. Solvent: Water; I = 0.1 M $NaClO_4$; T = 25.0 ± 0.2 °C; l = 1 cm; $[7]_{tot}$ = 2.00 × 10^{-5} M; (1) pH = 3.08; (2) pH = 7.39. The charges have been omitted for the sake of clarity. The inset of (**A**) shows the spectral variation at 250 nm as a function of pH. The UV-visible absorption spectra have not been corrected from dilution effects.

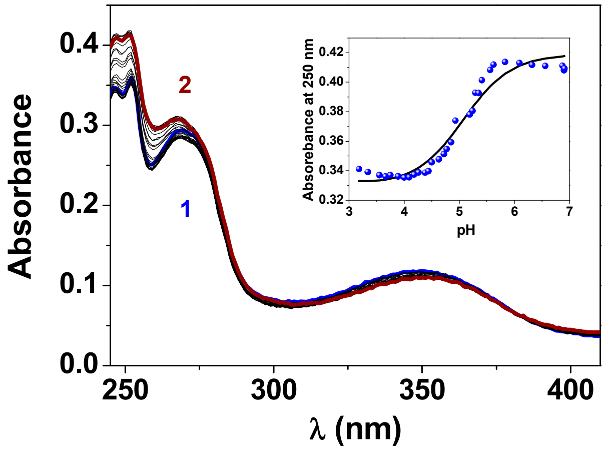

(**A**)

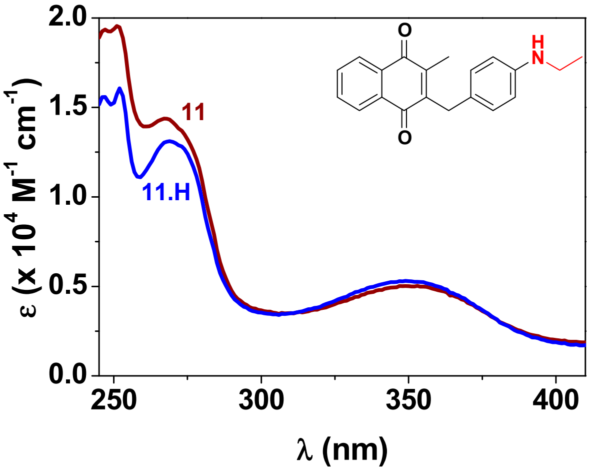

(**B**)

Figure S2. (**A**) Absorption spectrophotometric titration of compound 11 as a function of pH and (**B**) electronic spectra of 11 and its protonated species 11.H. Solvent: Water/DMSO (91/9 v/v); I = 0.1 M $NaClO_4$; T = 25.0 ± 0.2 °C; l = 1 cm; $[11]_{tot}$ = 2.18 × 10^{-5} M; (1) pH = 3.18; (2) pH = 6.91. The charges have been omitted for the sake of clarity. The inset of (**A**) shows the spectral variation at 250 nm as a function of pH. The UV-visible absorption spectra have not been corrected from dilution effects.

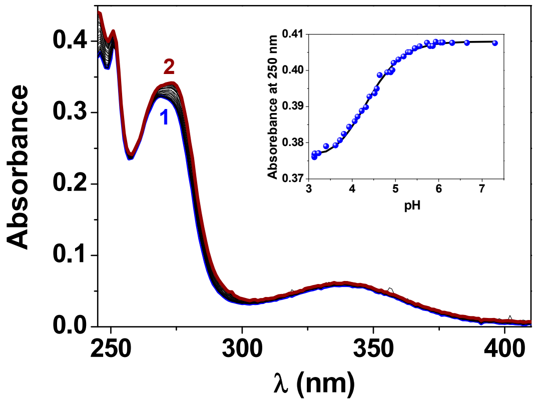

(**A**)

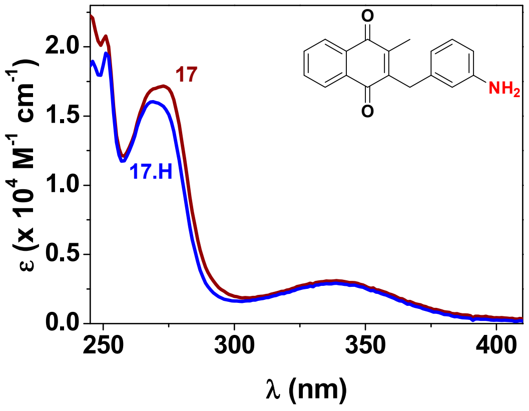

(**B**)

Figure S3. (**A**) Absorption spectrophotometric titration of compound 17 as a function of pH and (**B**) electronic spectra of 17 and its protonated species 17.H. Solvent: Water; I = 0.1 M $NaClO_4$; T = 25.0 ± 0.2 °C; l = 1 cm; $[17]_{tot}$ = 2.00 × 10^{-5} M; (1) pH = 3.13; (2) pH = 7.30. The charges have been omitted for the sake of clarity. The inset of (**A**) shows the spectral variation at 250 nm as a function of pH. The UV-visible absorption spectra have not been corrected from dilution effects.

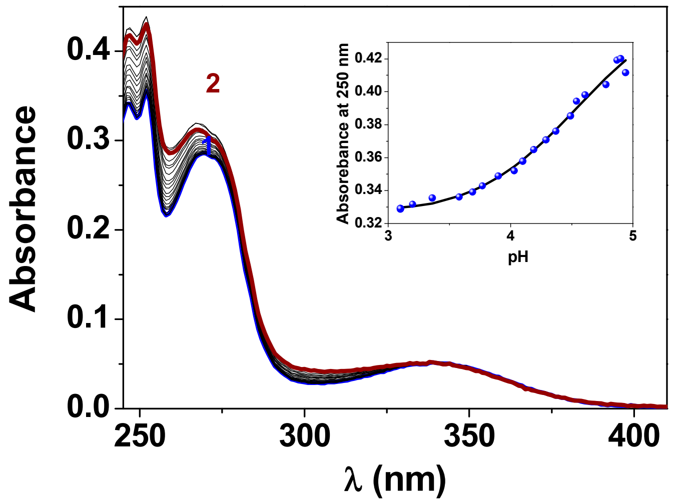

(**A**)

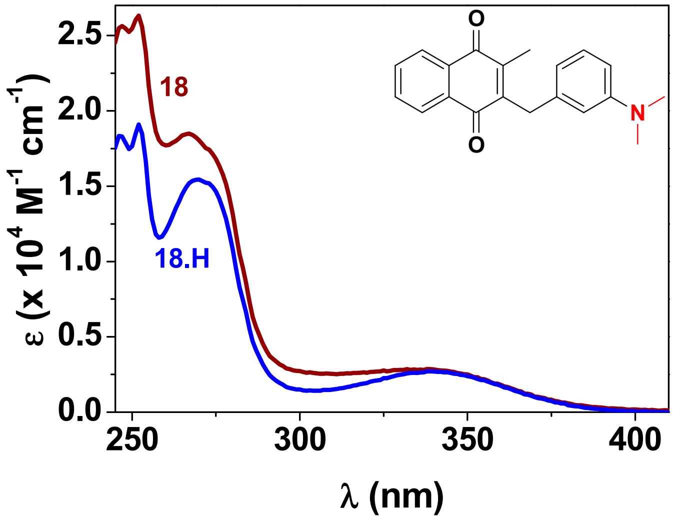

(**B**)

Figure S4. (**A**) Absorption spectrophotometric titration of compound 18 as a function of pH and (**B**) electronic spectra of 18 and its protonated species 18.H. Solvent: Water; $I = 0.1$ M $NaClO_4$; $T = 25.0 \pm 0.2$ °C; $l = 1$ cm; $[18]_{tot} = 1.84 \times 10^{-5}$ M; (1) pH = 3.10; (2) pH = 4.94. The charges have been omitted for the sake of clarity. The inset of (**A**) shows the spectral variation at 250 nm as a function of pH. The UV-visible absorption spectra have not been corrected from dilution effects.

2. ^{1}H-NMR and ^{13}C-NMR spectra

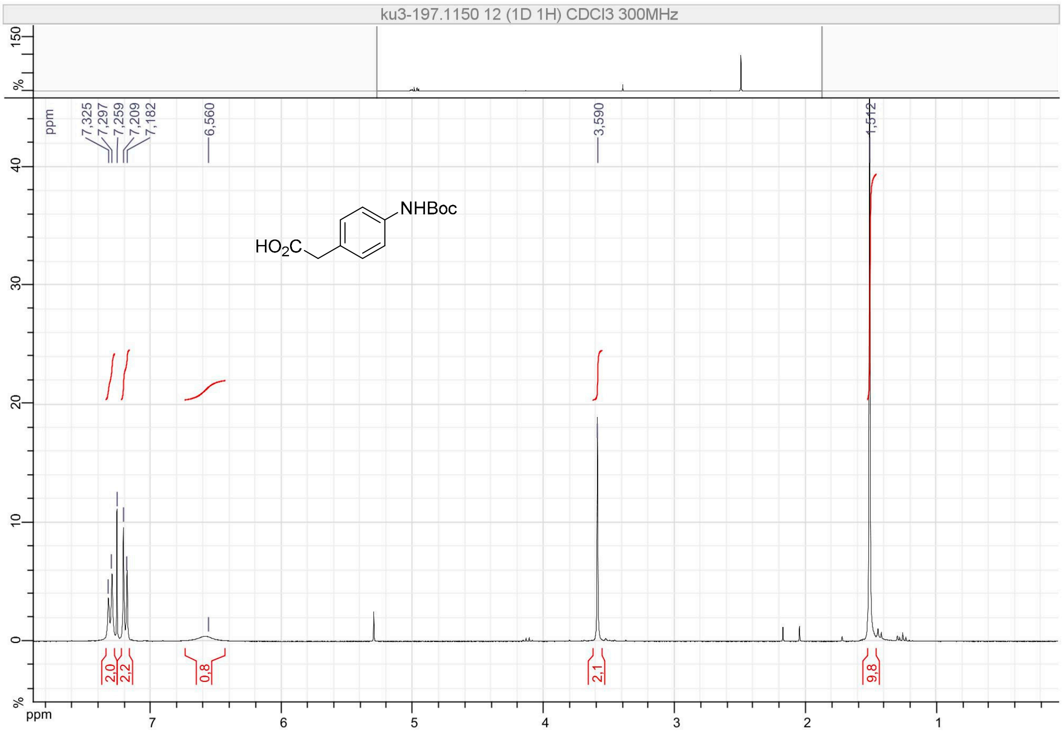

Figure S5. ^{1}H-NMR (300 MHz, $CDCl_3$) spectrum of compound **5**.

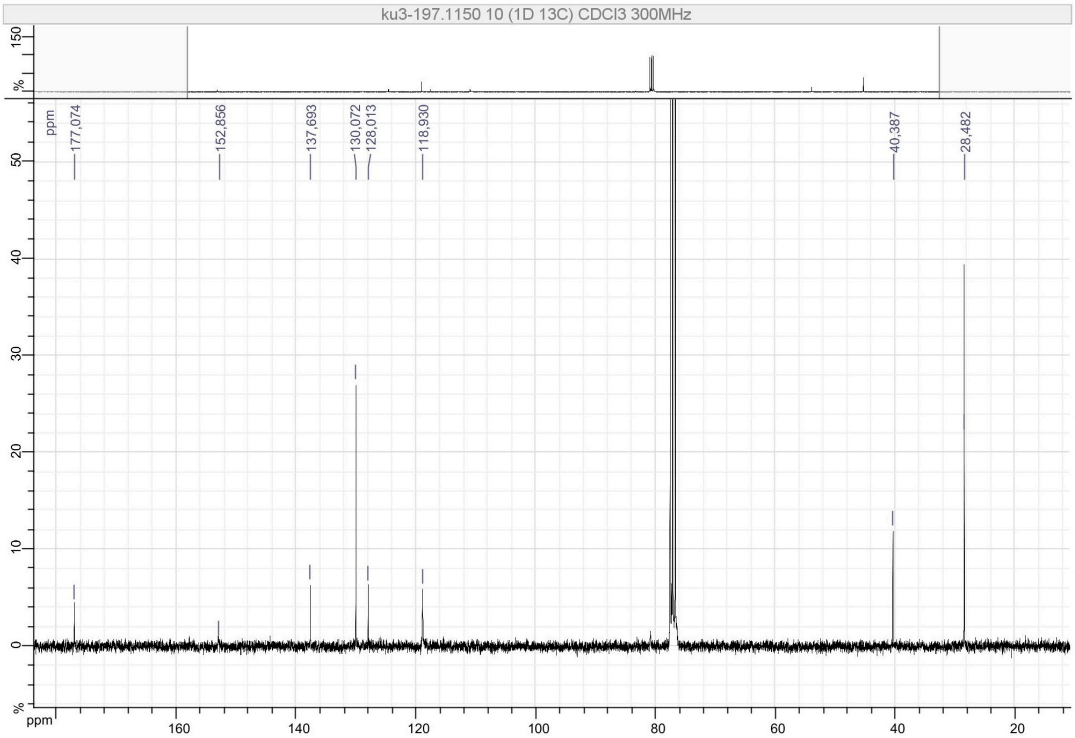

Figure S6. ^{13}C-NMR (75 MHz, $CDCl_3$) spectrum of compound **5**.

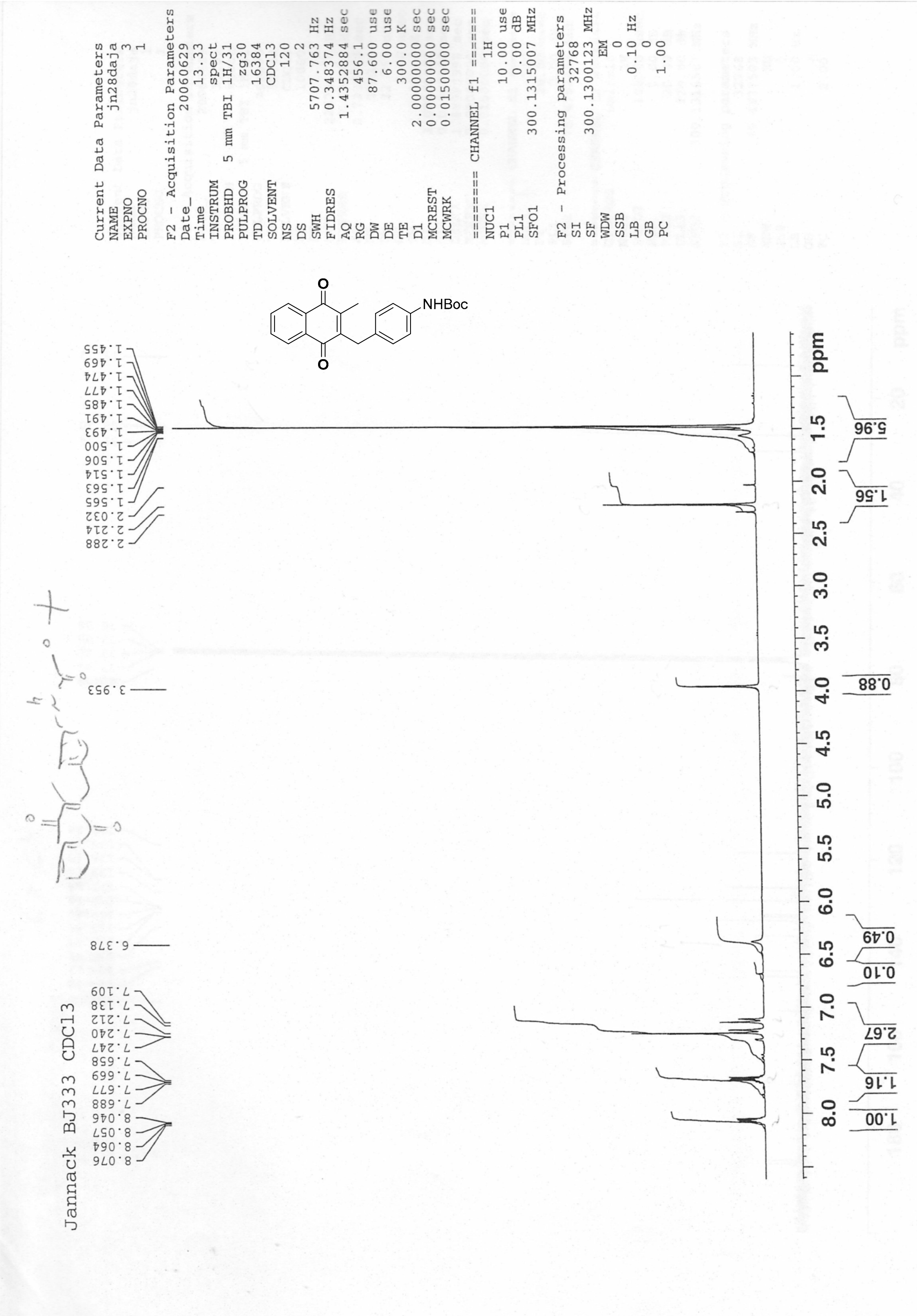

Figure S7. ^{1}H NMR (300 MHz, $CDCl_3$) spectrum of compound **6**.

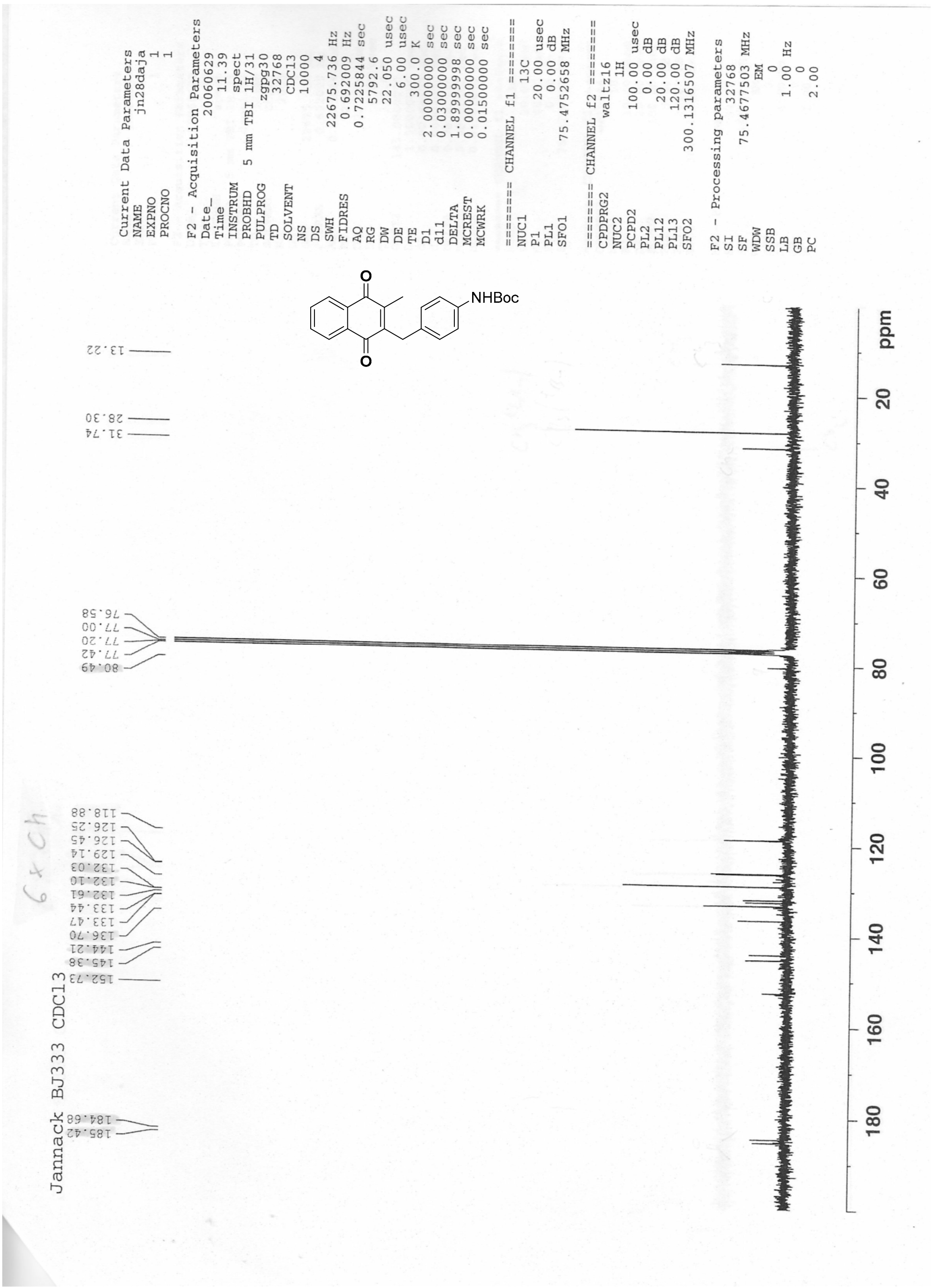

Figure S8. ^{13}C-NMR (75 MHz, $CDCl_3$) spectrum of compound **6**.

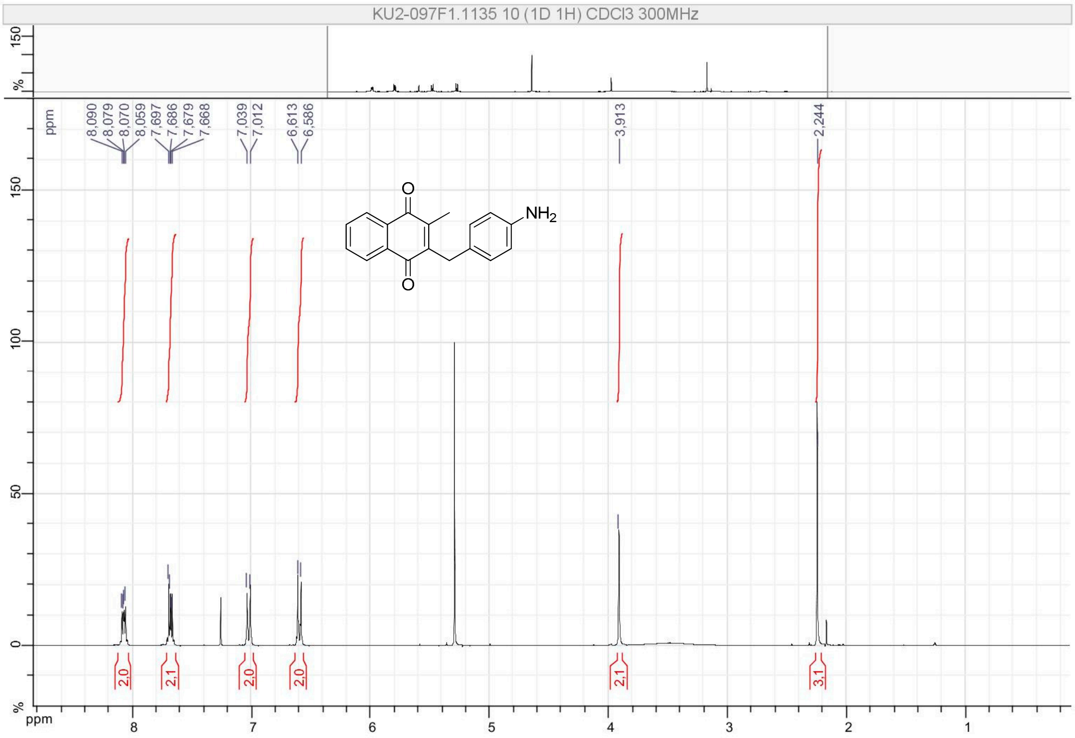

Figure S9. ^{1}H-NMR (300 MHz, $CDCl_3$) spectrum of compound **7**.

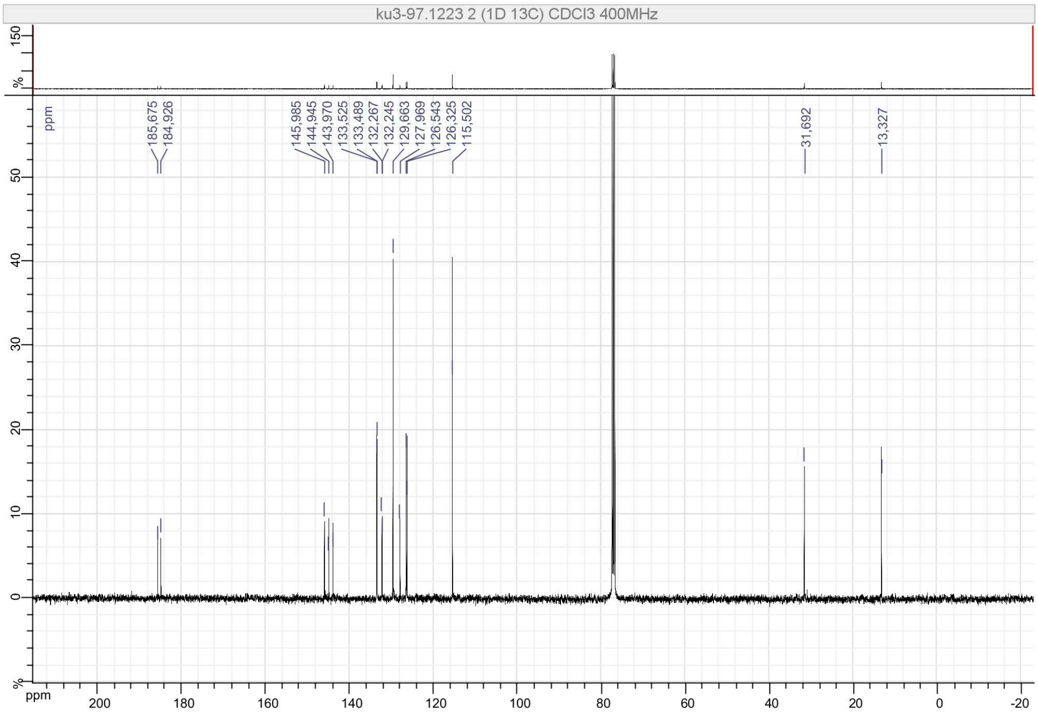

Figure S10. ^{13}C-NMR (100 MHz, $CDCl_3$) spectrum of compound **7**.

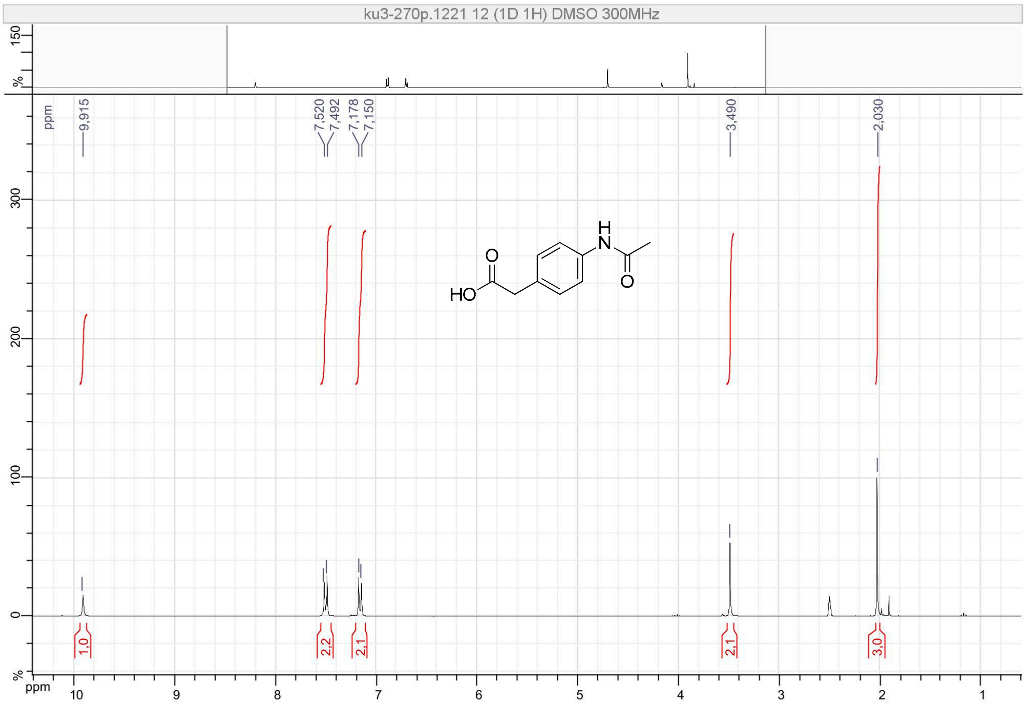

Figure S11. ^{1}H-NMR (300 MHz, DMSO-d6) spectrum of compound **8**.

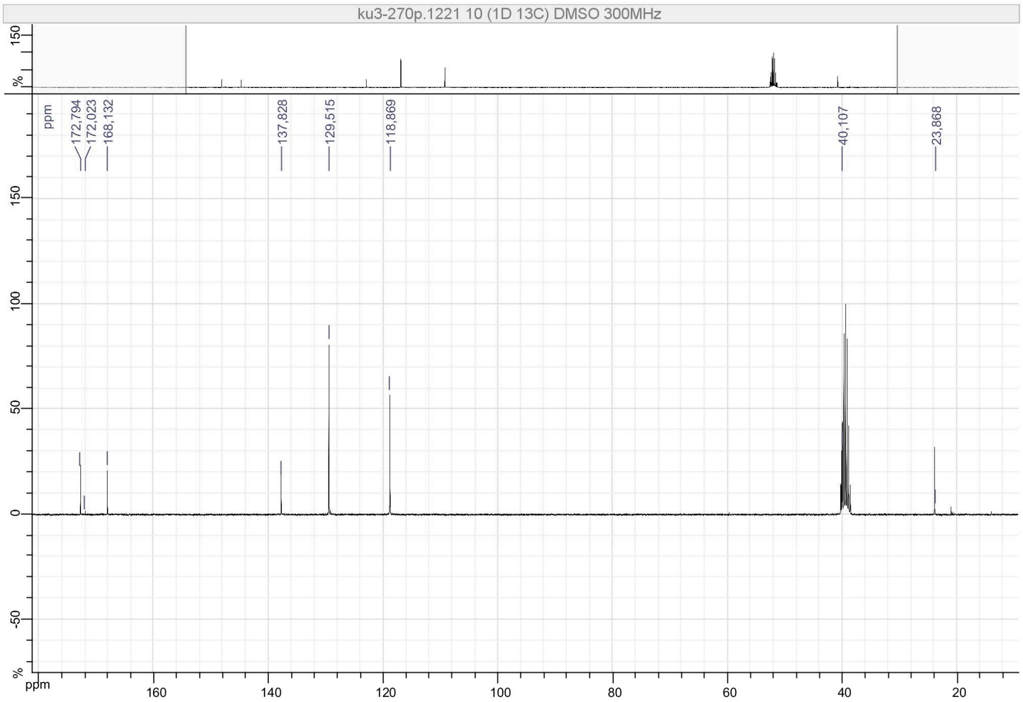

Figure S12. ^{13}C-NMR (75 MHz, DMSO-d6) spectrum of compound **8**.

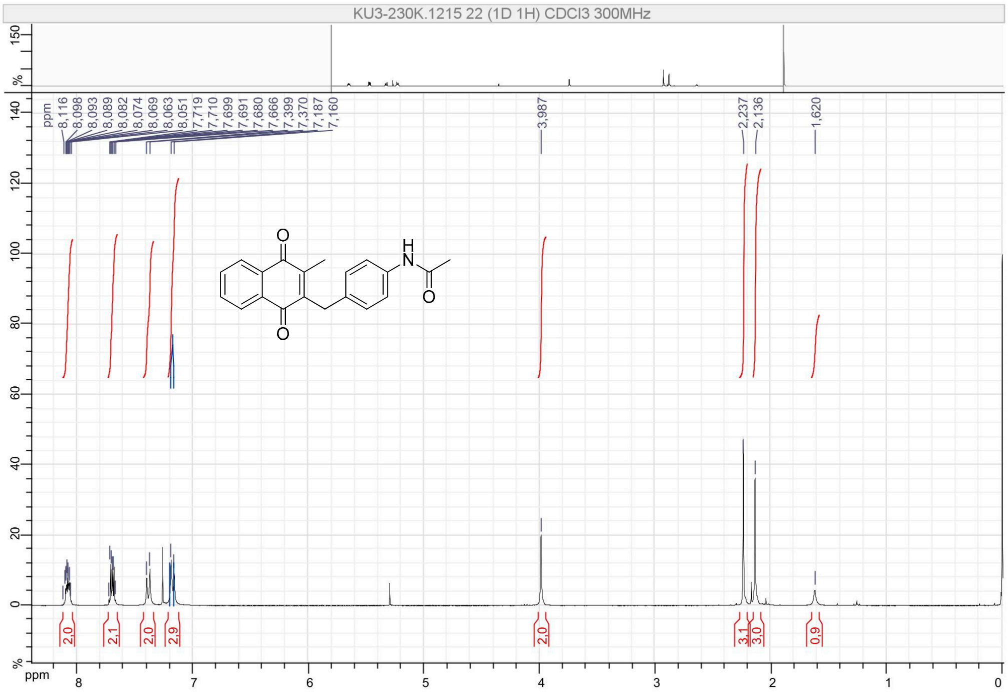

Figure S13. ^{1}H-NMR (300 MHz, $CDCl_3$) spectrum of compound **9**.

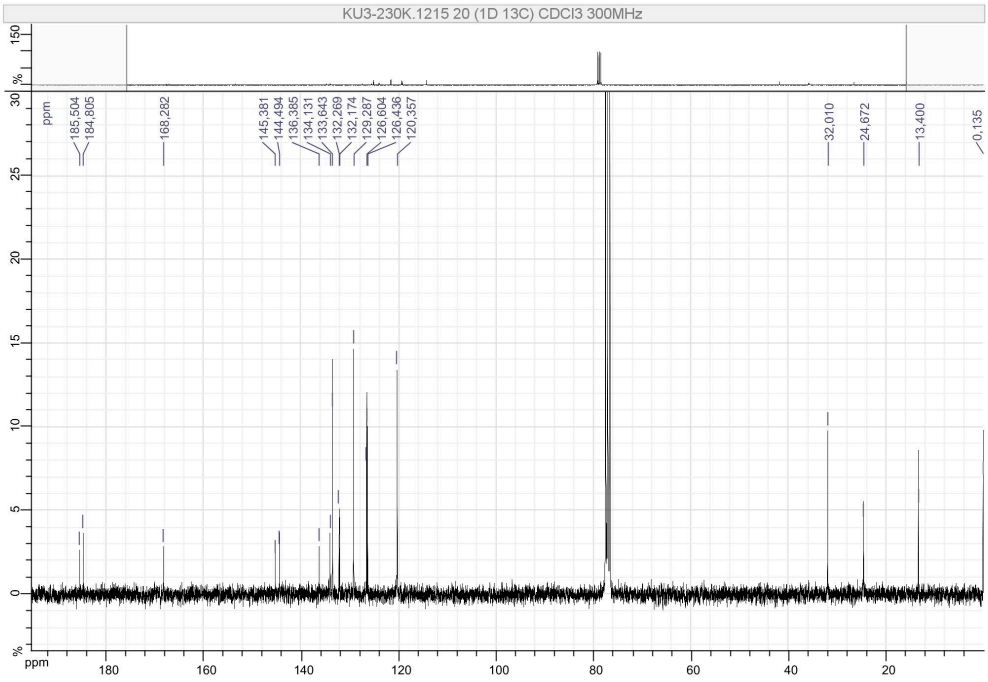

Figure S14. ^{13}C-NMR (75 MHz, $CDCl_3$) spectrum of compound **9**.

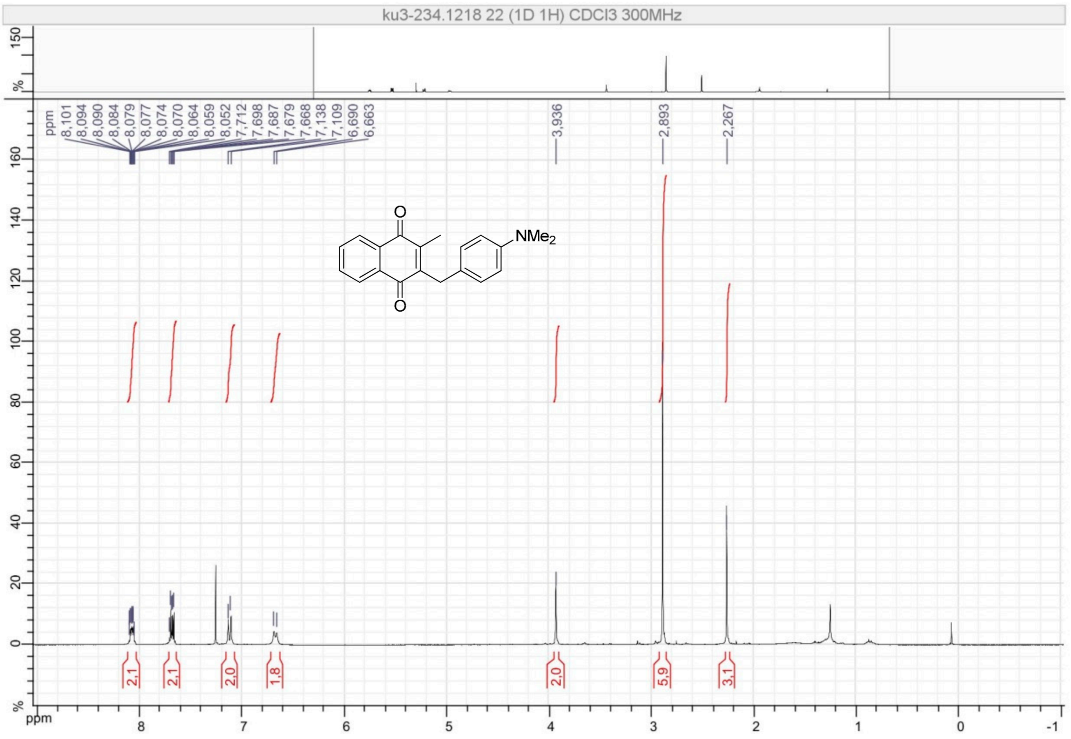

Figure S15. ^{1}H-NMR (300 MHz, $CDCl_3$) spectrum of compound **3**.

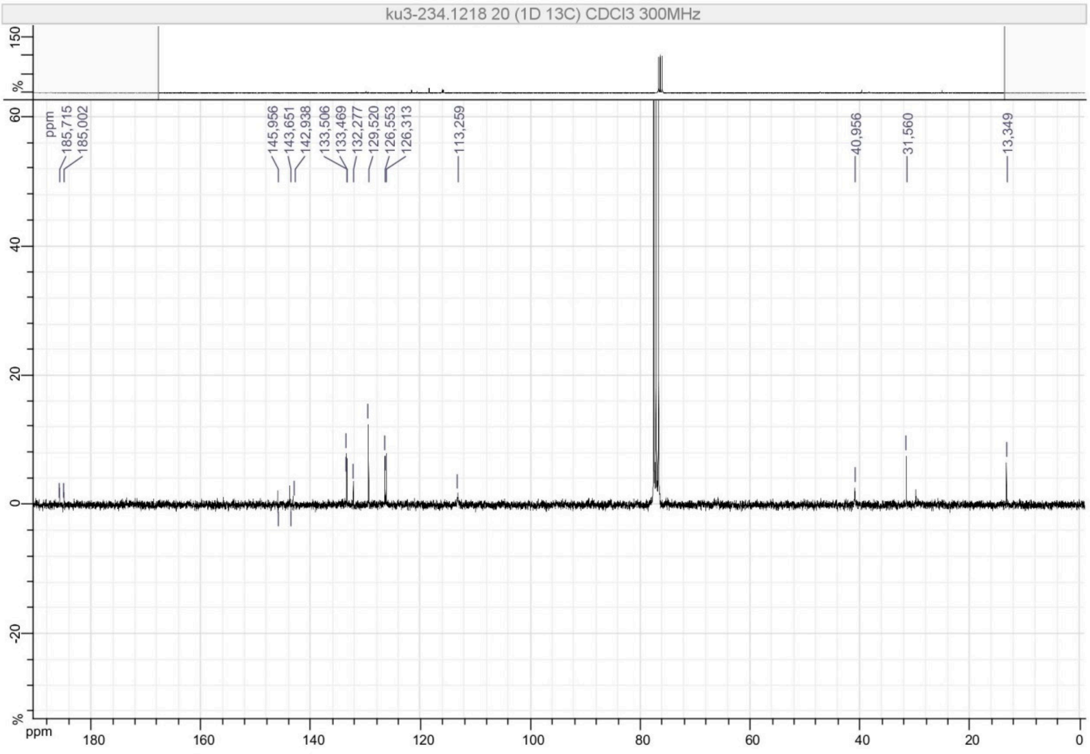

Figure S16. ^{13}C-NMR (75 MHz, $CDCl_3$) spectrum of compound **3**.

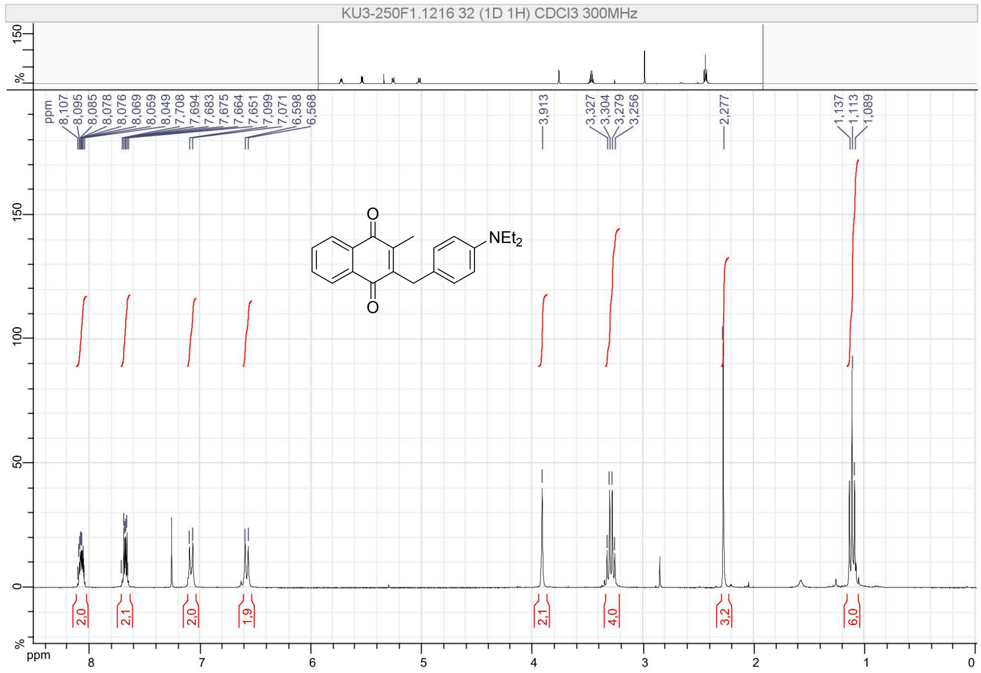

Figure S17. ^{1}H-NMR (300 MHz, $CDCl_3$) spectrum of compound **11**.

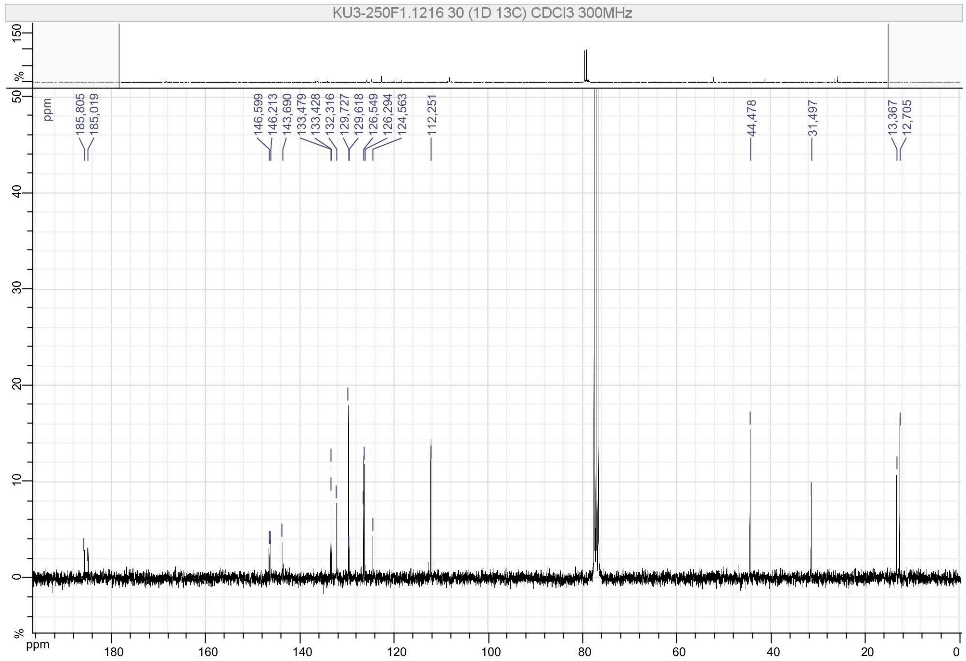

Figure S18. ^{13}C-NMR (75 MHz, $CDCl_3$) spectrum of compound **11**.

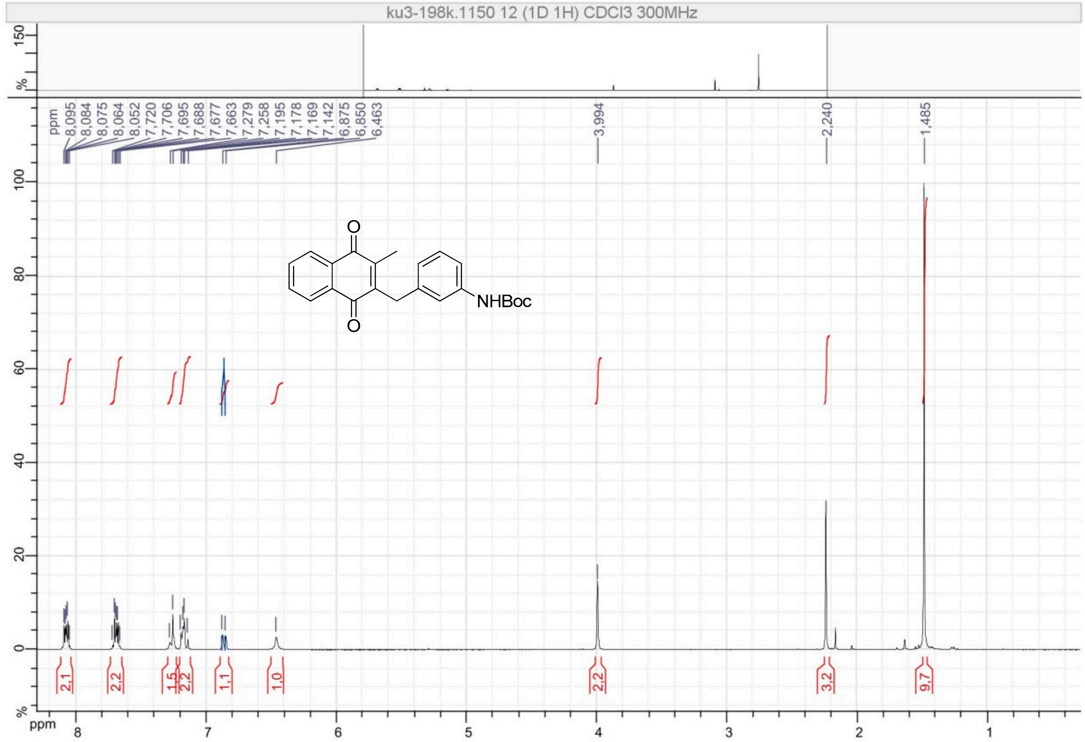

Figure S19. ^{1}H-NMR (300 MHz, $CDCl_3$) spectrum of compound **14**.

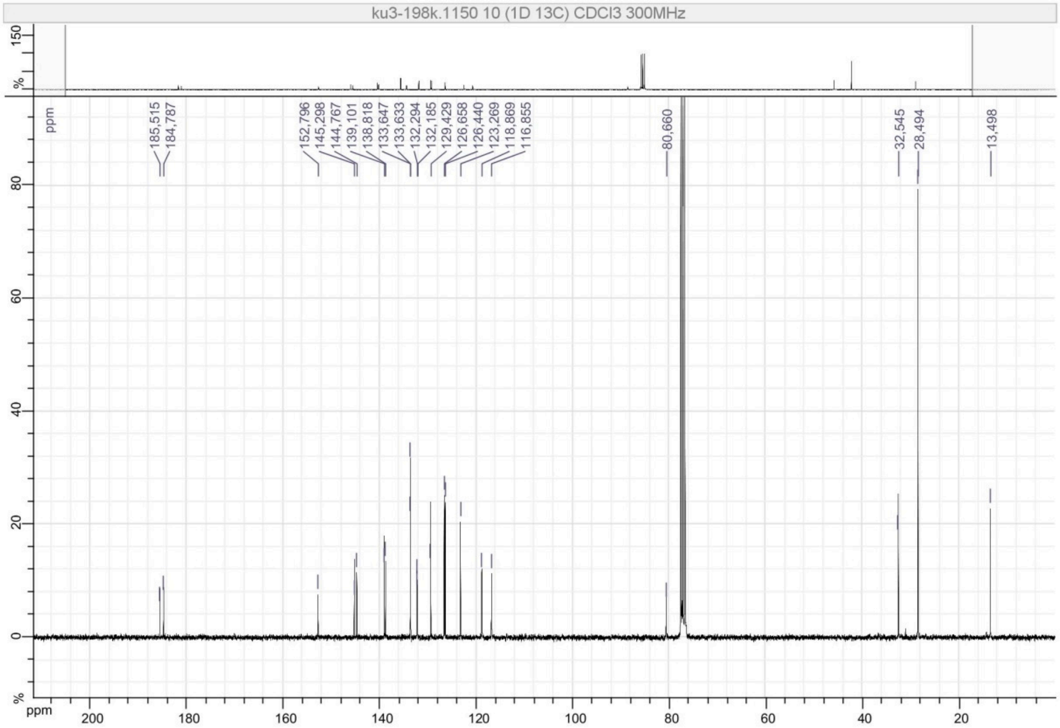

Figure S20. ^{13}C-NMR (75 MHz, $CDCl_3$) spectrum of compound **14**.

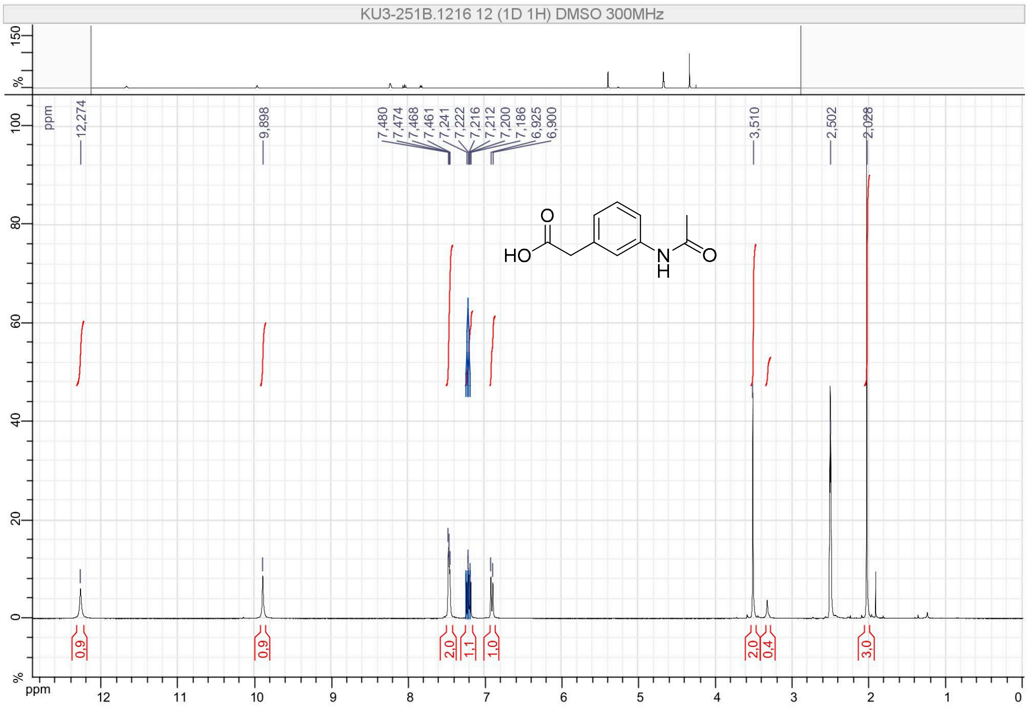

Figure S21. ^{1}H-NMR (300 MHz, DMSO-d$_6$) spectrum of compound **15**.

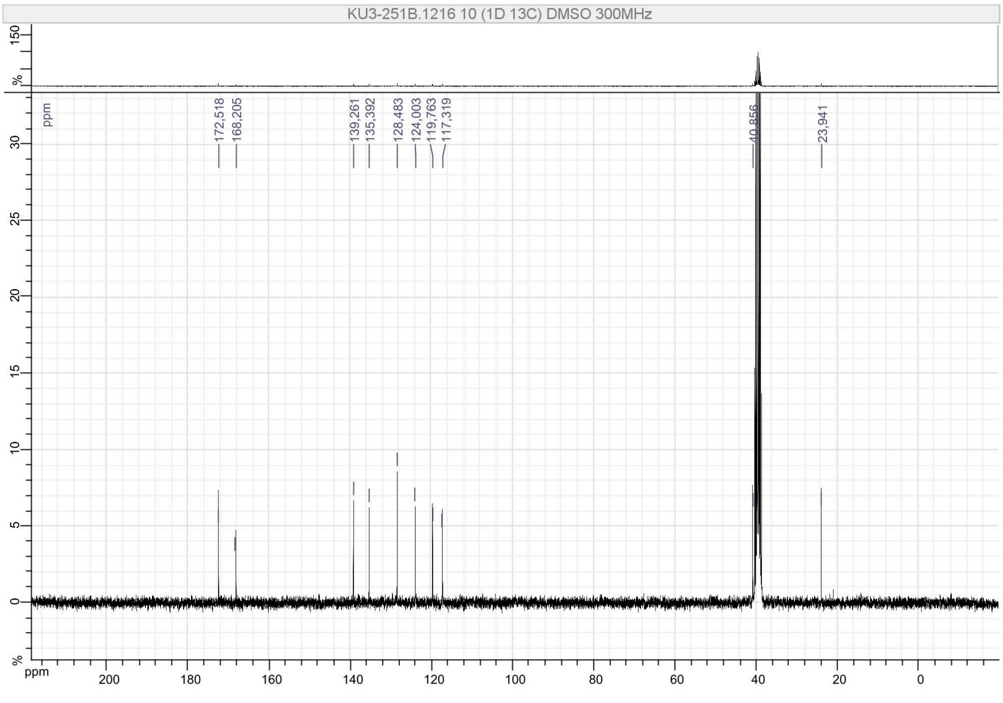

Figure S22. ^{13}C-NMR (75 MHz, DMSO-d$_6$) spectrum of compound **15**.

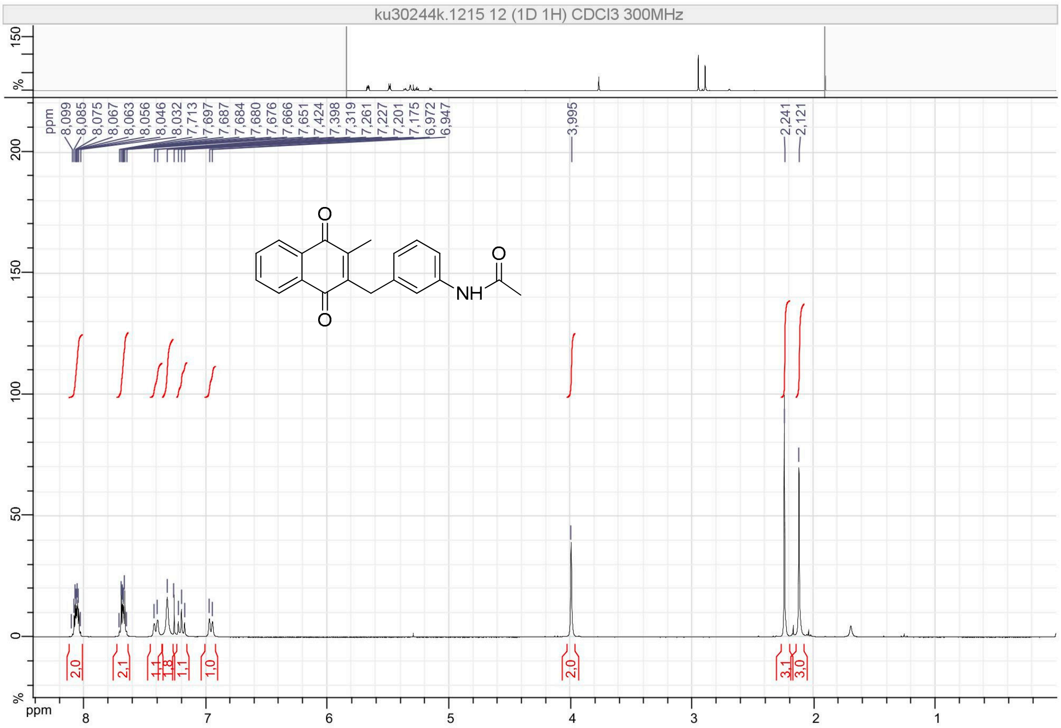

Figure S23. ^{1}H-NMR (300 MHz, $CDCl_3$) spectrum of compound **16**.

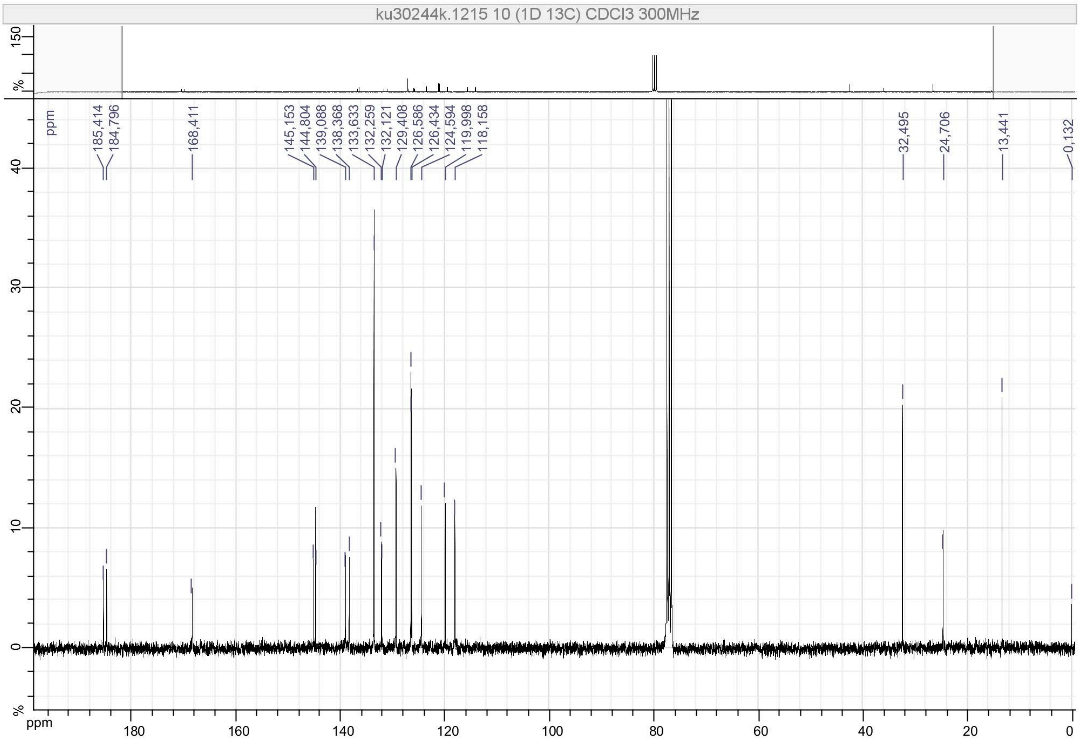

Figure S24. ^{13}C-NMR (75 MHz, $CDCl_3$) spectrum of compound **16**.

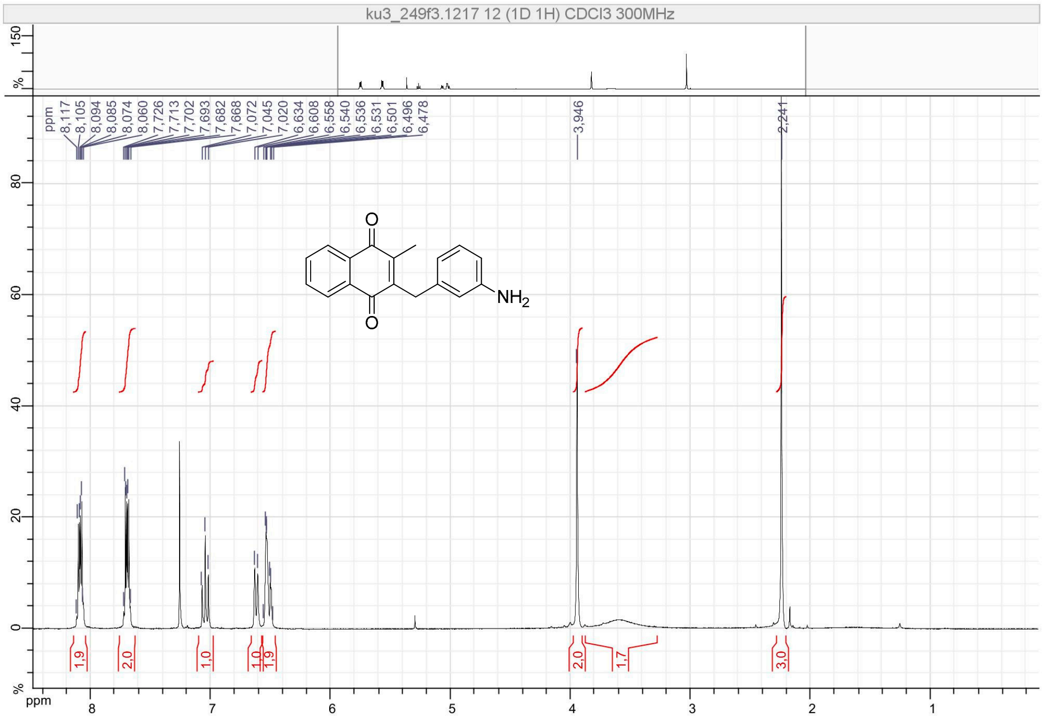

Figure S25. ^{1}H-NMR (300 MHz, $CDCl_3$) spectrum of compound **17**.

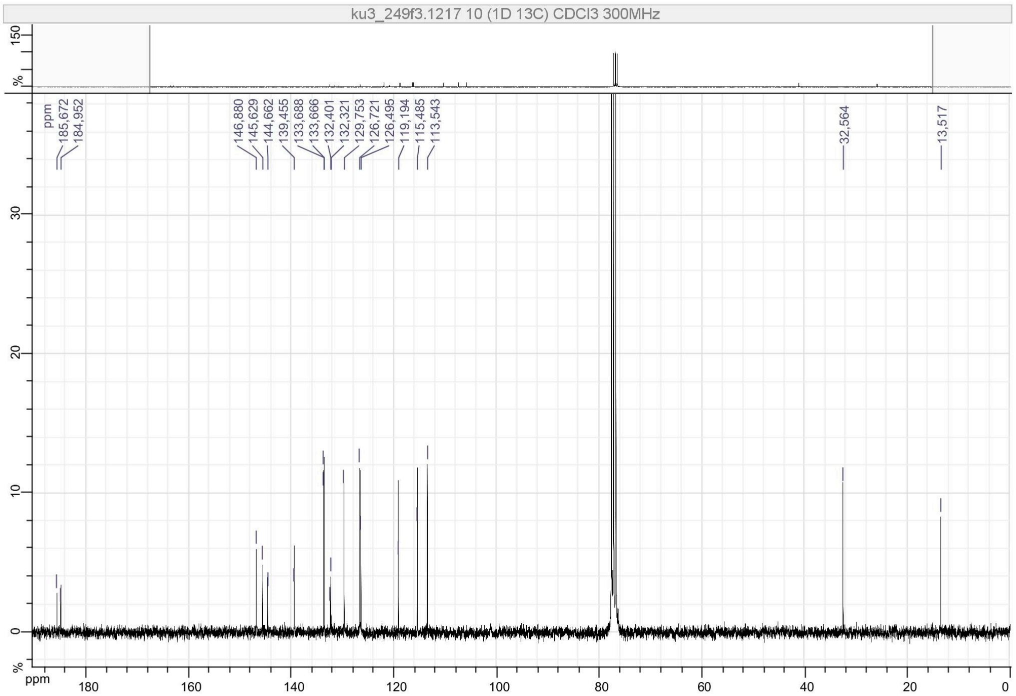

Figure S26. ^{13}C-NMR (75 MHz, $CDCl_3$) spectrum of compound **17**.

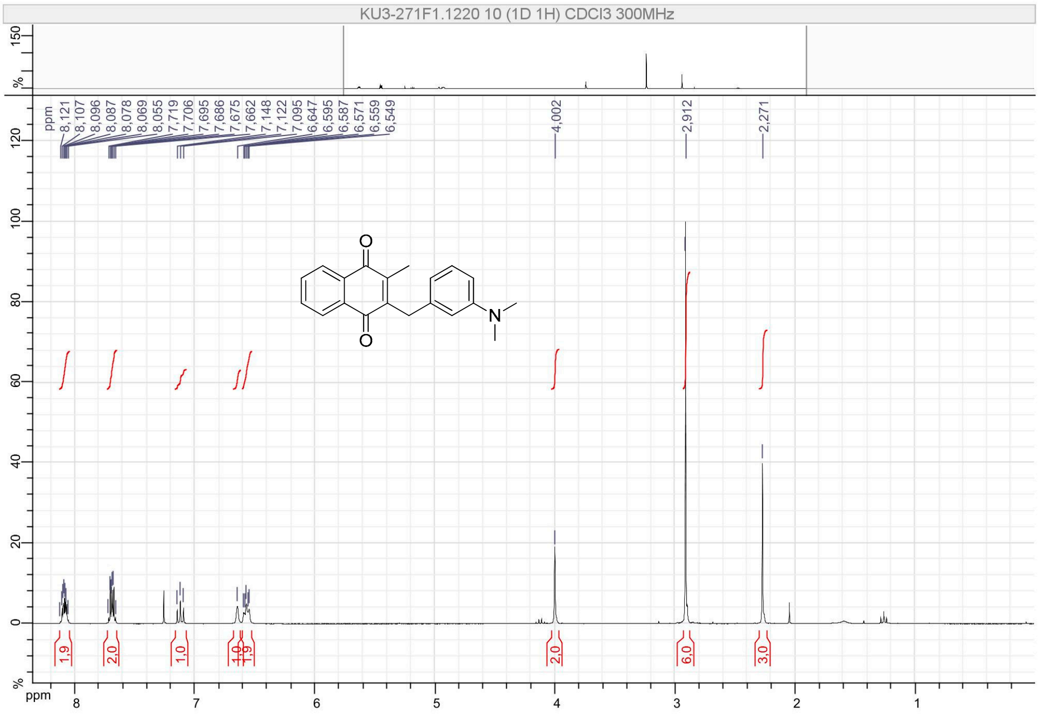

Figure S27. ^{1}H-NMR (300 MHz, $CDCl_3$) spectrum of compound **18**.

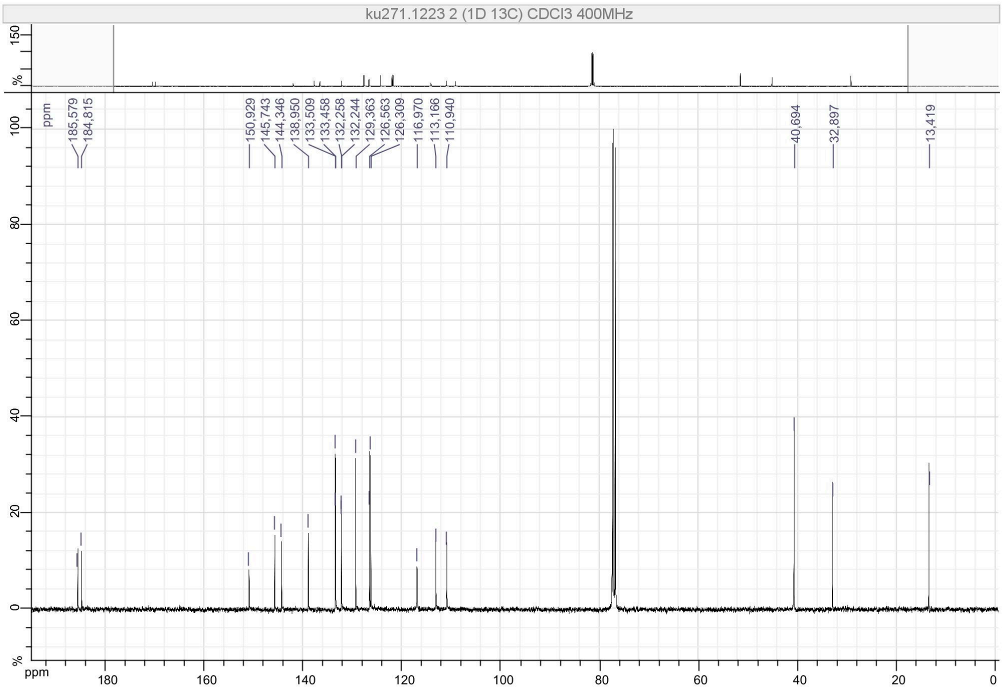

Figure S28. ^{13}C-NMR (100 MHz, $CDCl_3$) spectrum of compound **18**.

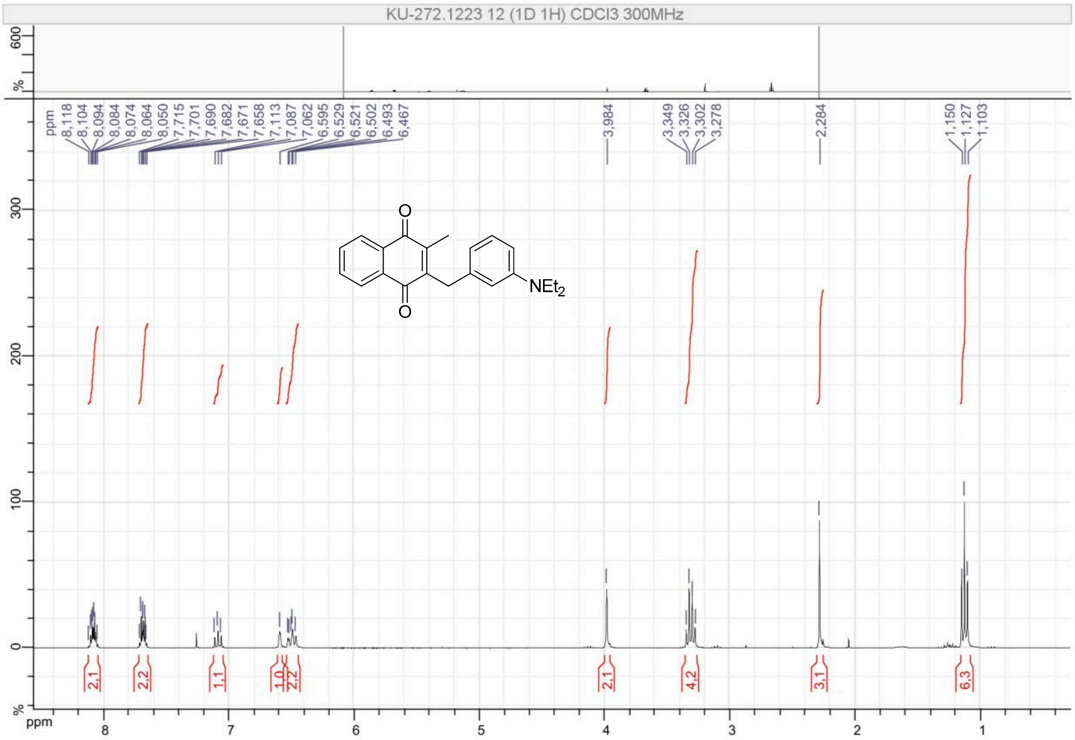

Figure S29. ^{1}H-NMR (300 MHz, $CDCl_3$) spectrum of compound **19**.

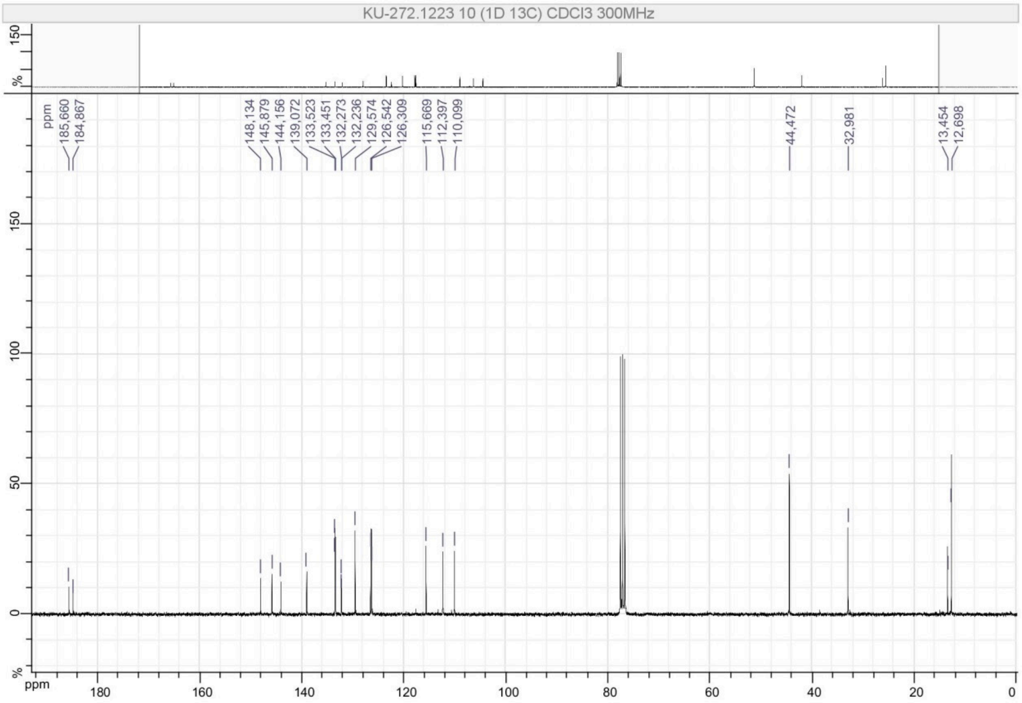

Figure S30. ^{13}C-NMR (75 MHz, $CDCl_3$) spectrum of compound **19**.

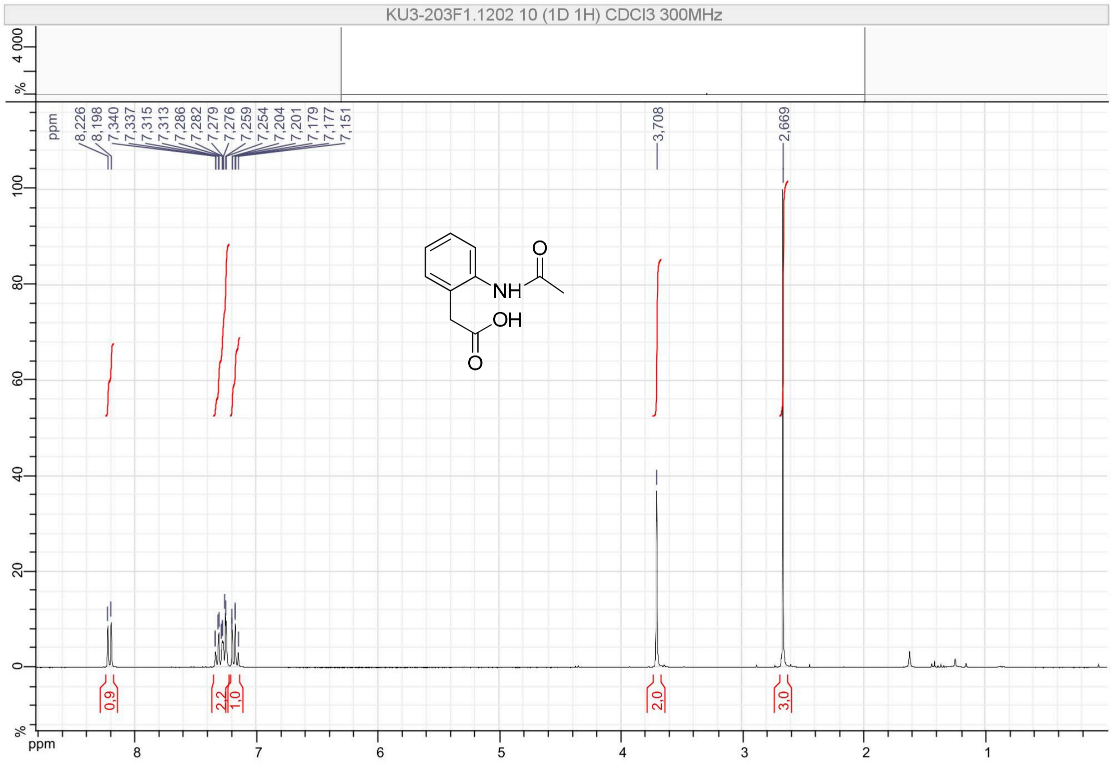

Figure S31. ^{1}H-NMR (300 MHz, $CDCl_3$) spectrum of compound **21a**.

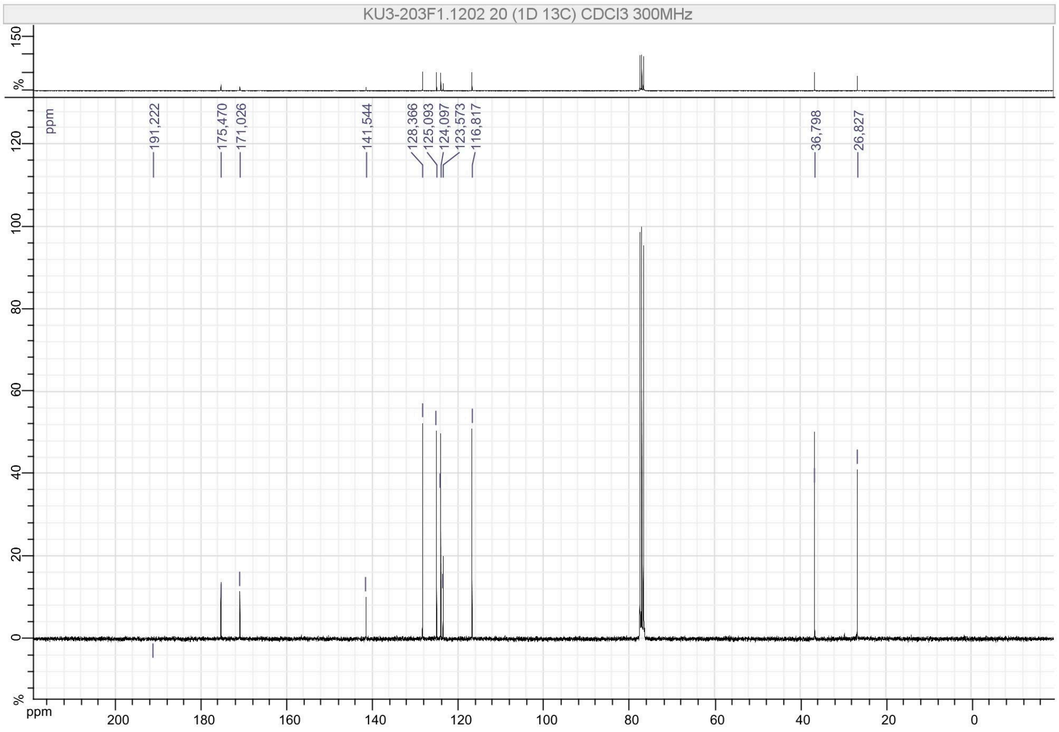

Figure S32. ^{13}C-NMR (75 MHz, $CDCl_3$) spectrum of compound **21a**.

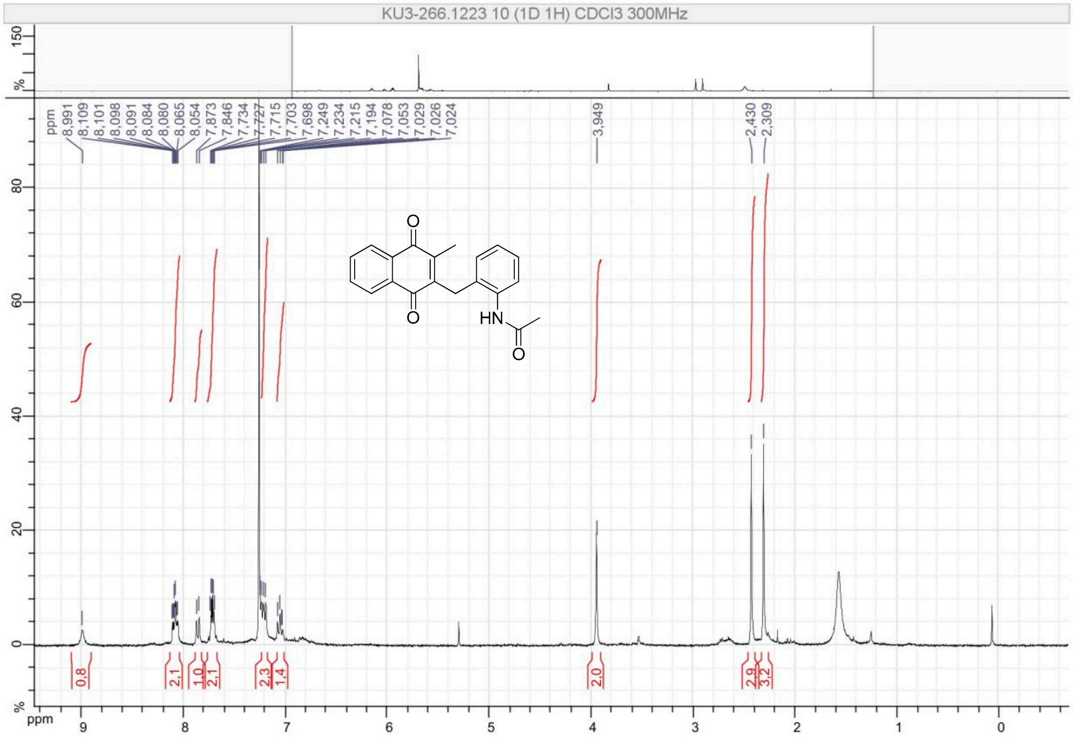

Figure S33. ^{1}H-NMR (300 MHz, $CDCl_3$) spectrum of compound **22**.

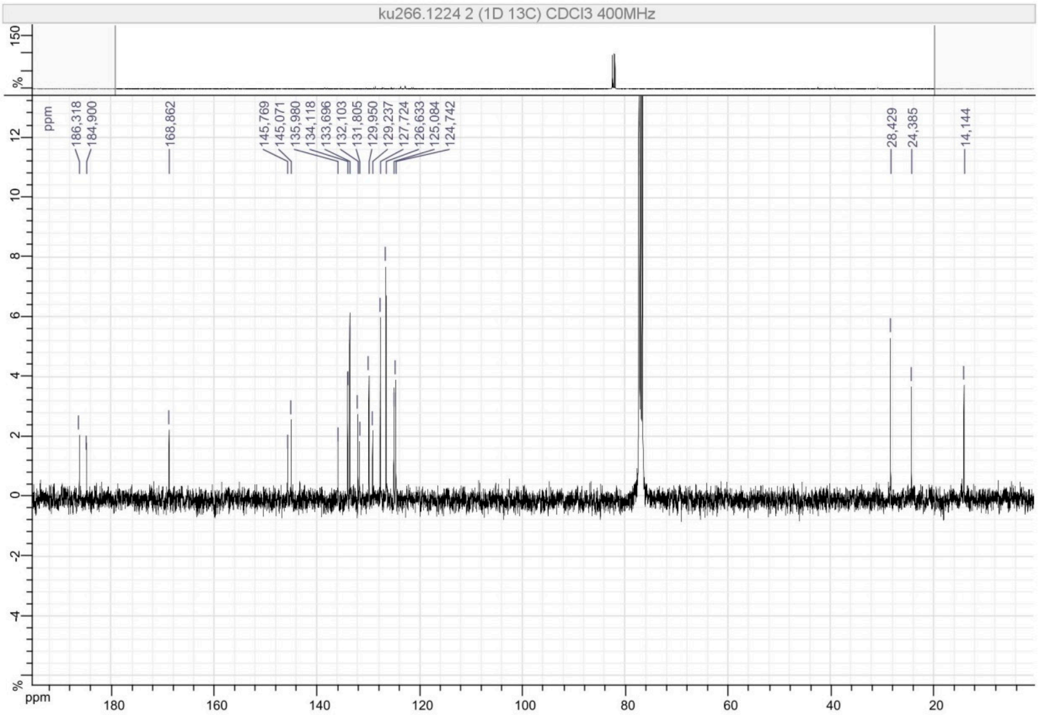

Figure S34. ^{13}C-NMR (100 MHz, $CDCl_3$) spectrum of compound **22**.

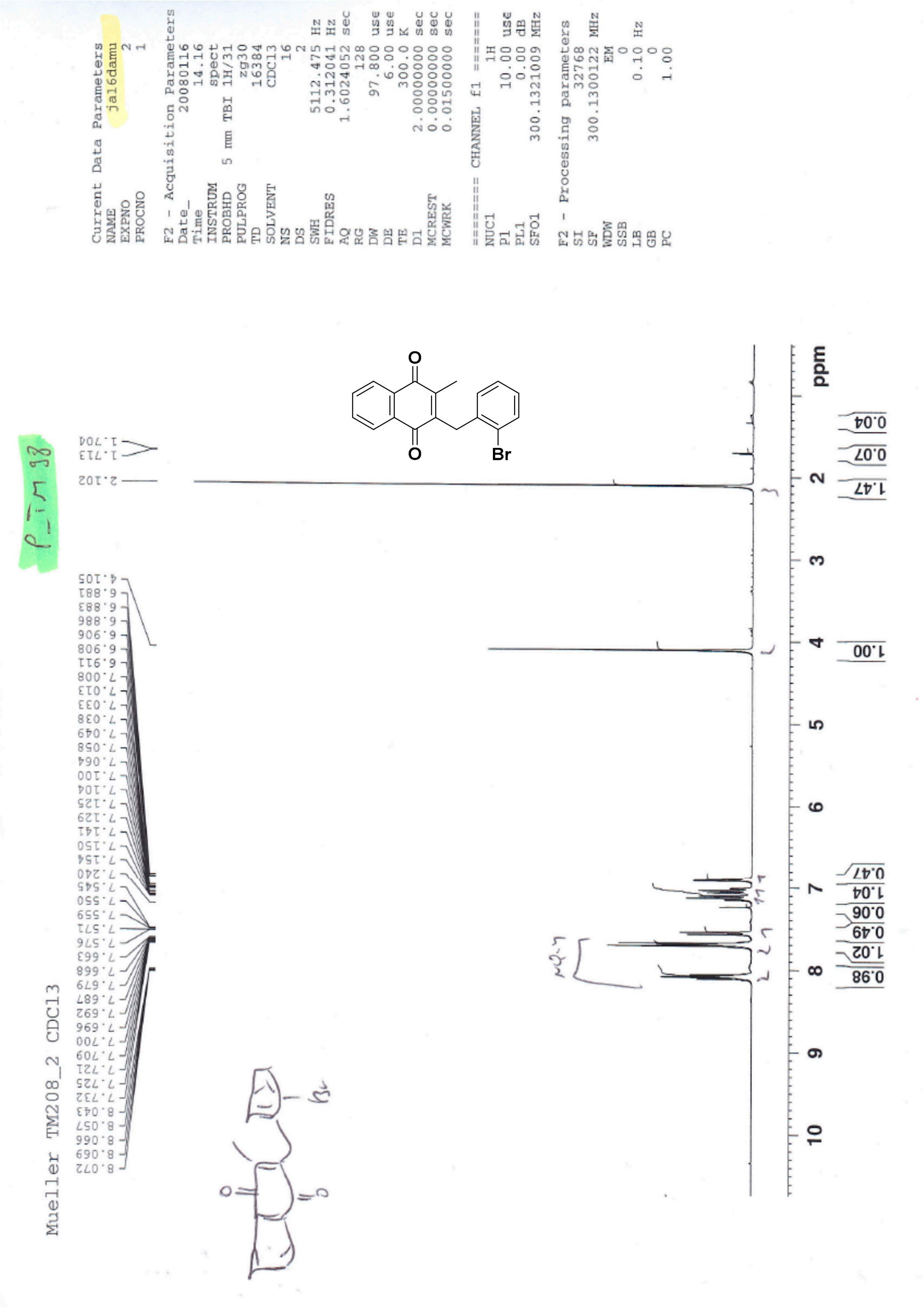

Figure S35. ^{1}H-NMR (300 MHz, $CDCl_3$) spectrum of compound **25a**.

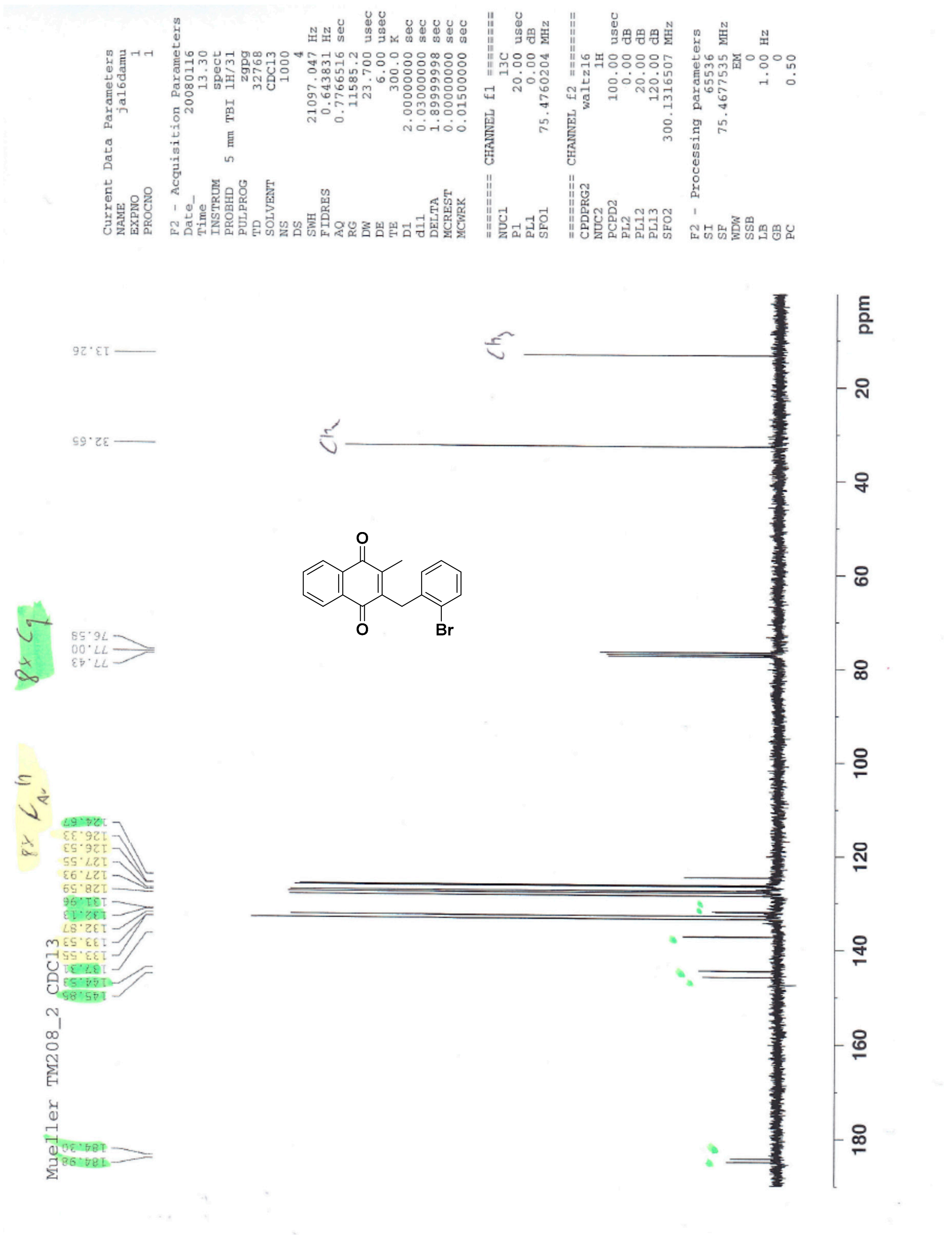

Figure S36. ^{13}C-NMR (75 MHz, $CDCl_3$) spectrum of compound **25a**.

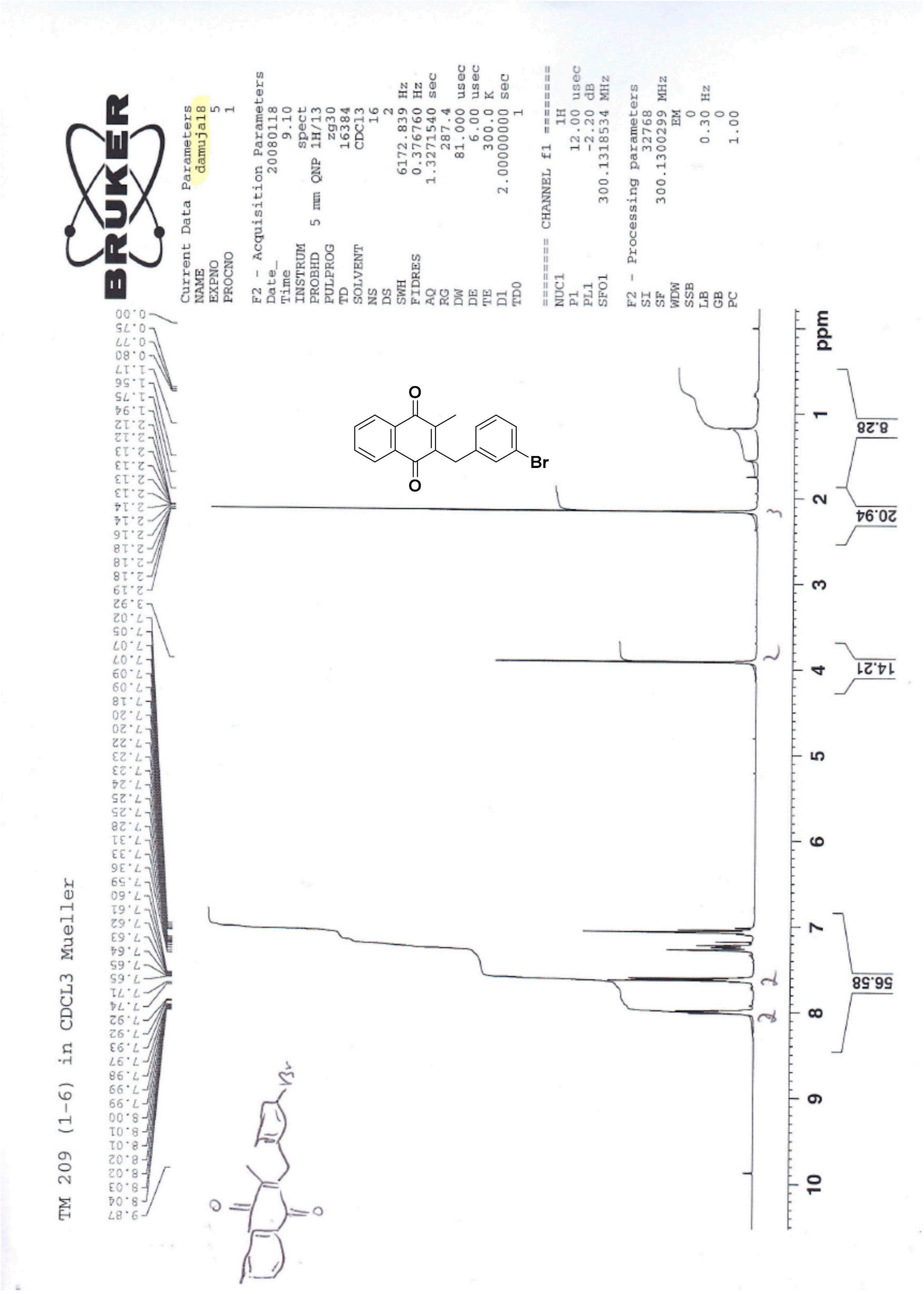

Figure S37. ^{1}H-NMR (300 MHz, $CDCl_3$) spectrum of compound **25b**.

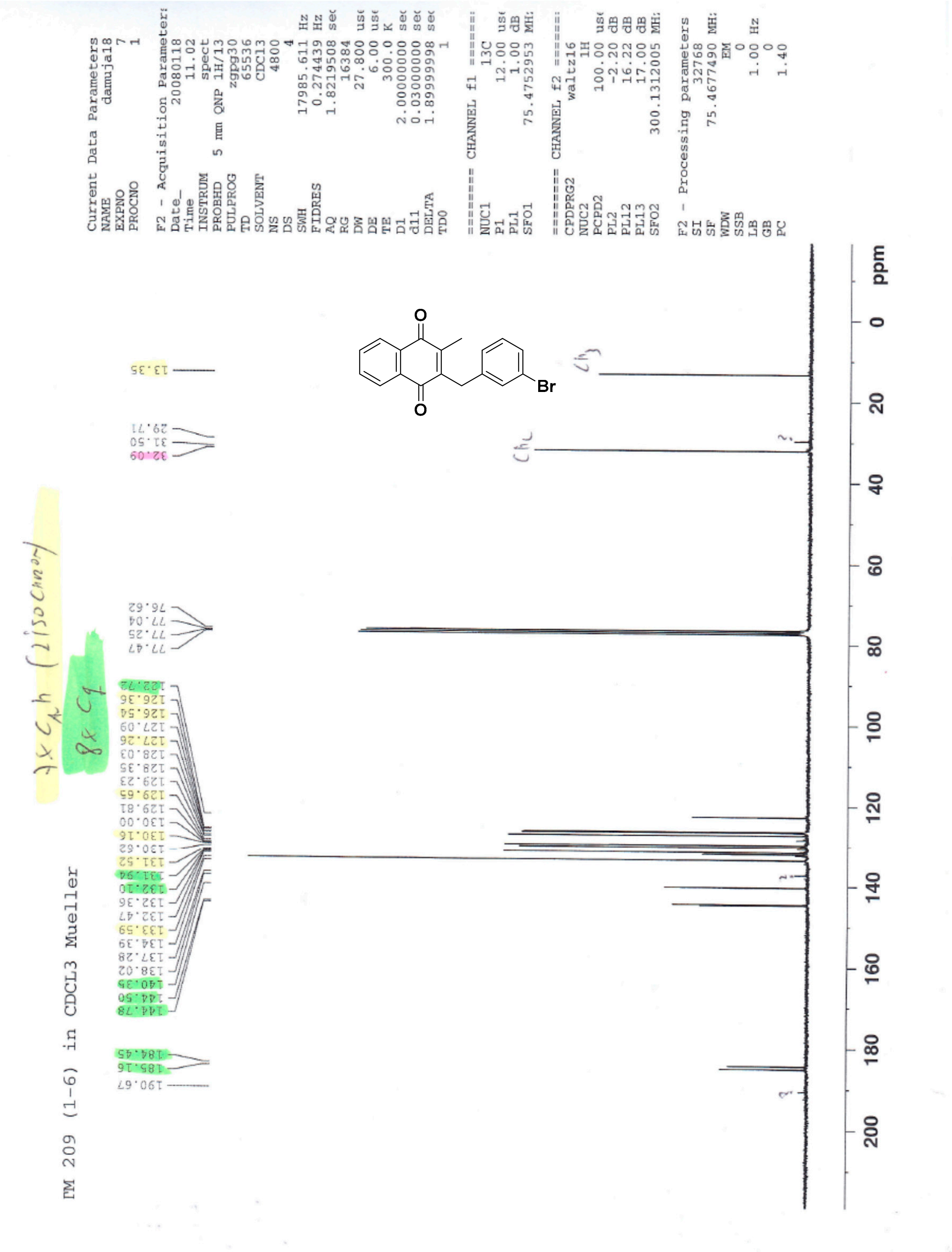

Figure S38. ^{13}C-NMR (75 MHz, $CDCl_3$) spectrum of compound **25b**.

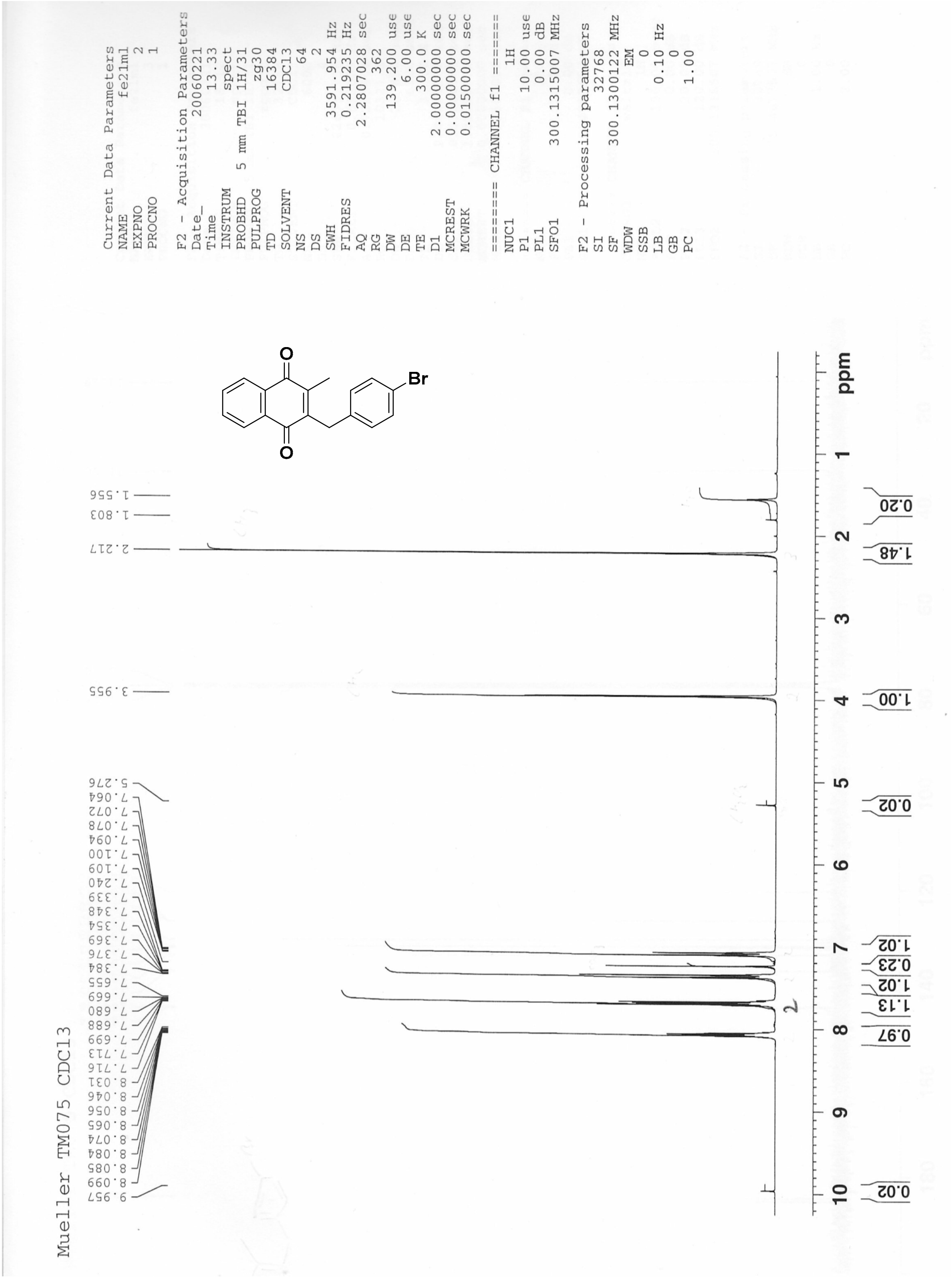

Figure S39. ^{1}H-NMR (300 MHz, $CDCl_3$) spectrum of compound **25c**.

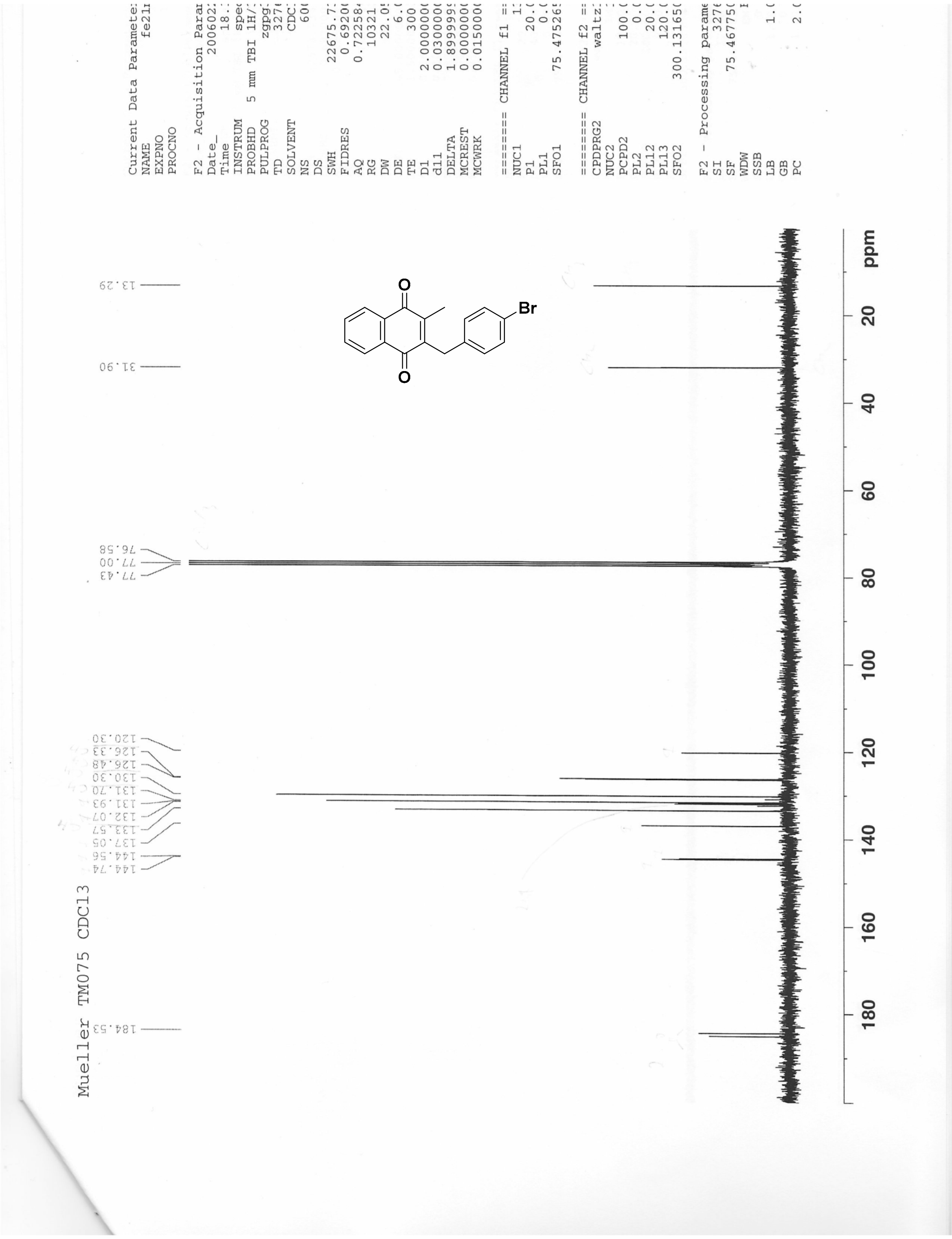

Figure S40. ^{13}C-NMR (75 MHz, $CDCl_3$) spectrum of compound **25c**.

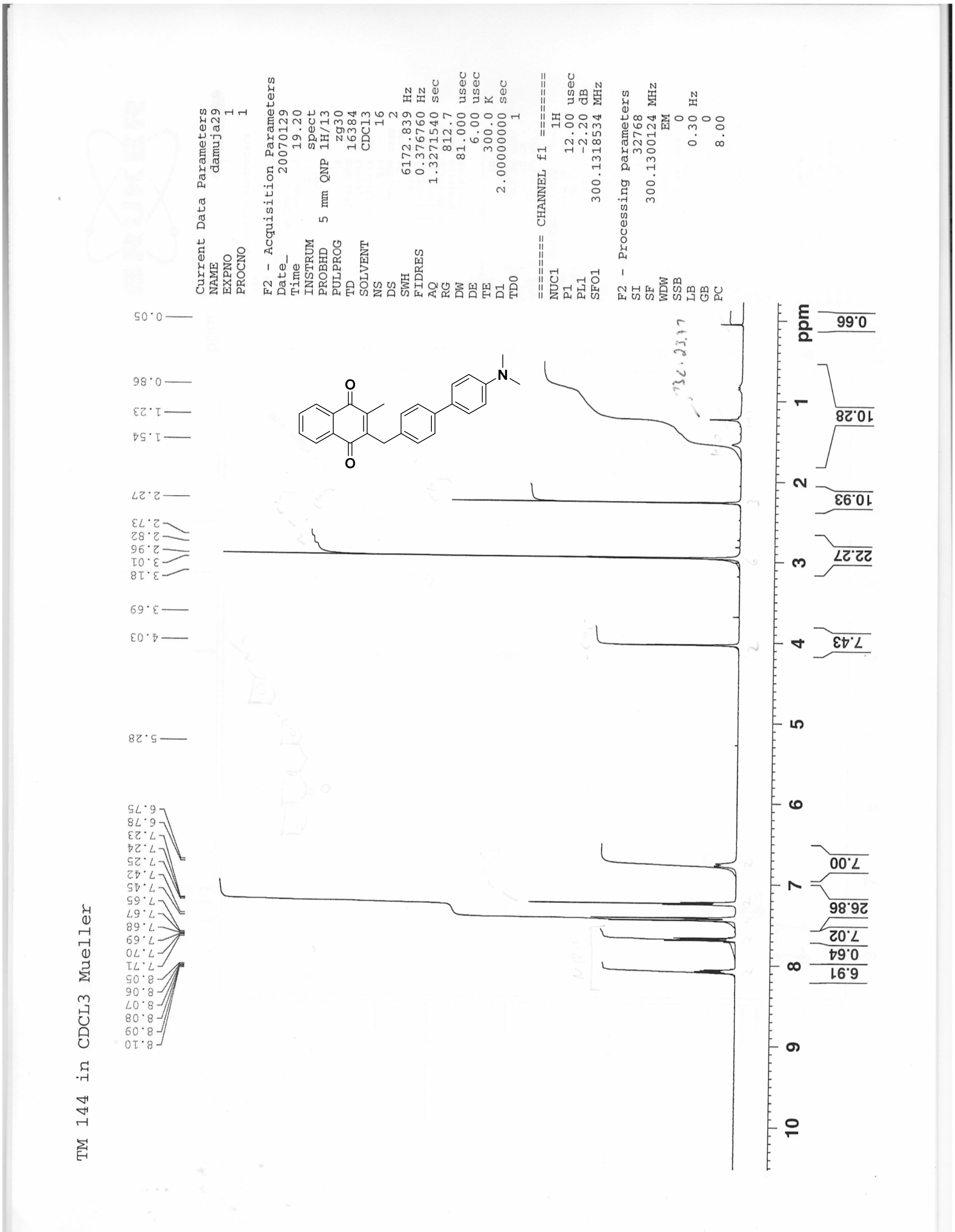

Figure S41. ^{1}H-NMR (300 MHz, $CDCl_3$) spectrum of compound **26**.

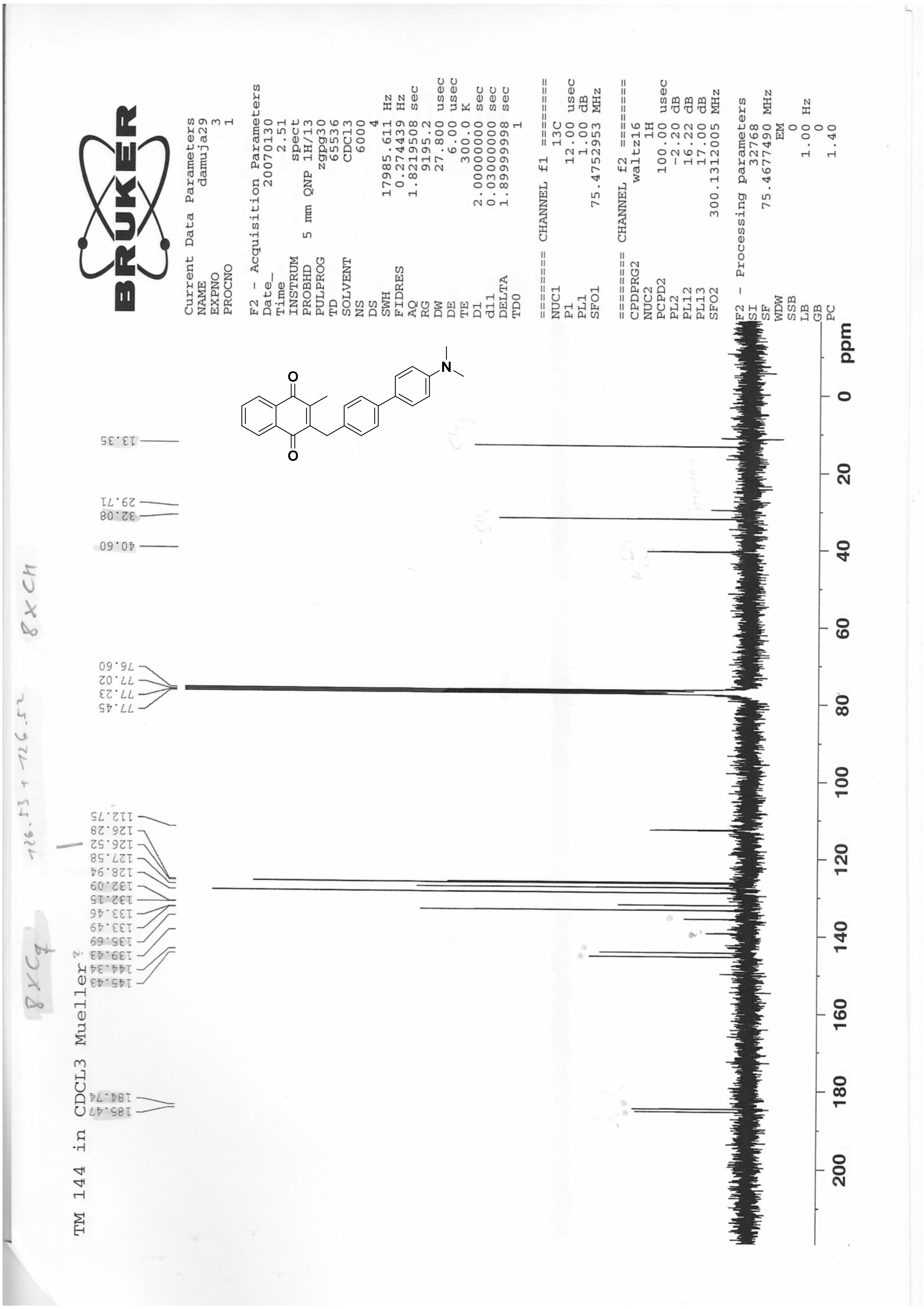

Figure S42. ^{13}C-NMR (75 MHz, $CDCl_3$) spectrum of compound **26**.

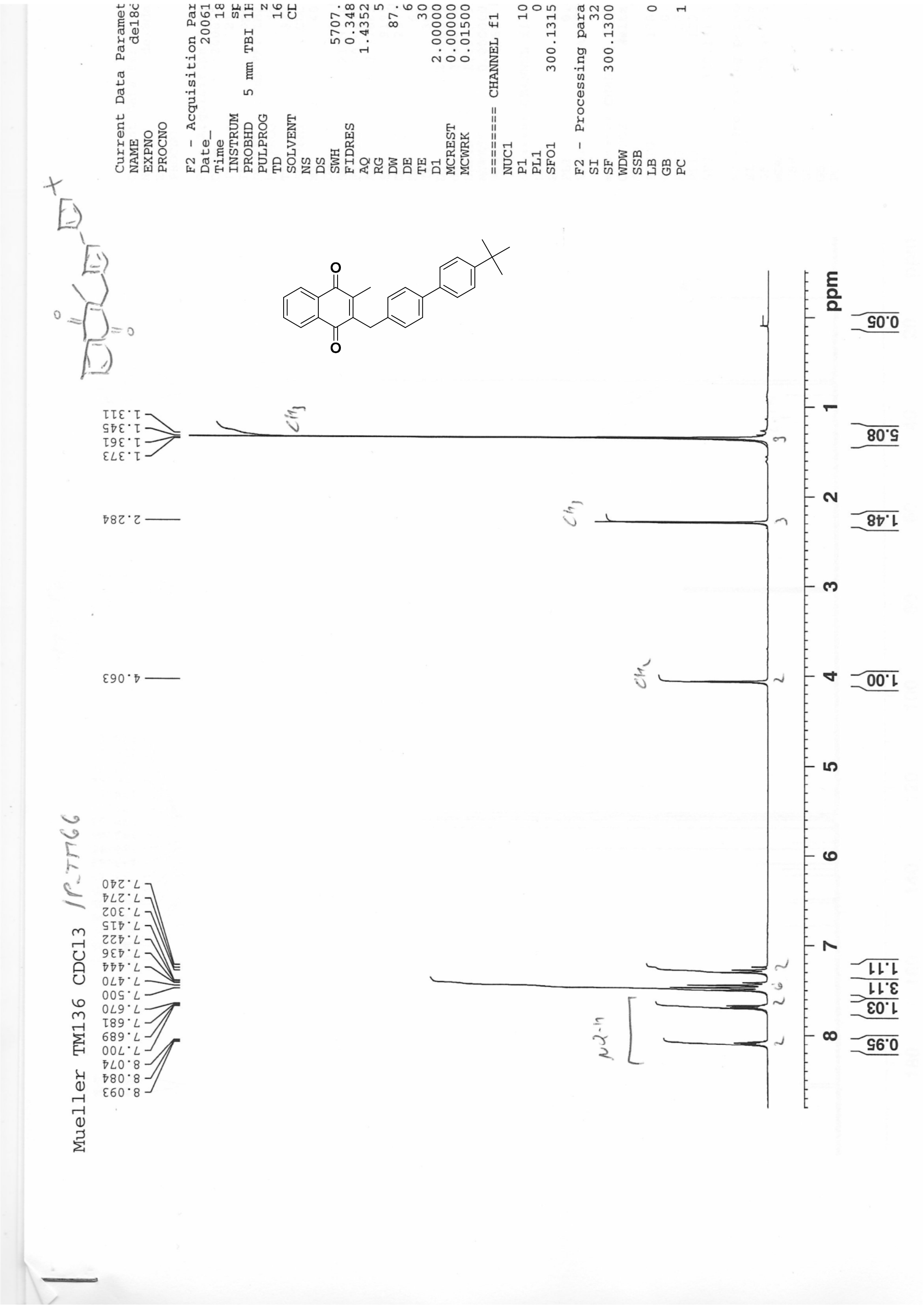

Figure S43. ^{1}H-NMR (300 MHz, $CDCl_3$) spectrum of compound **27**.

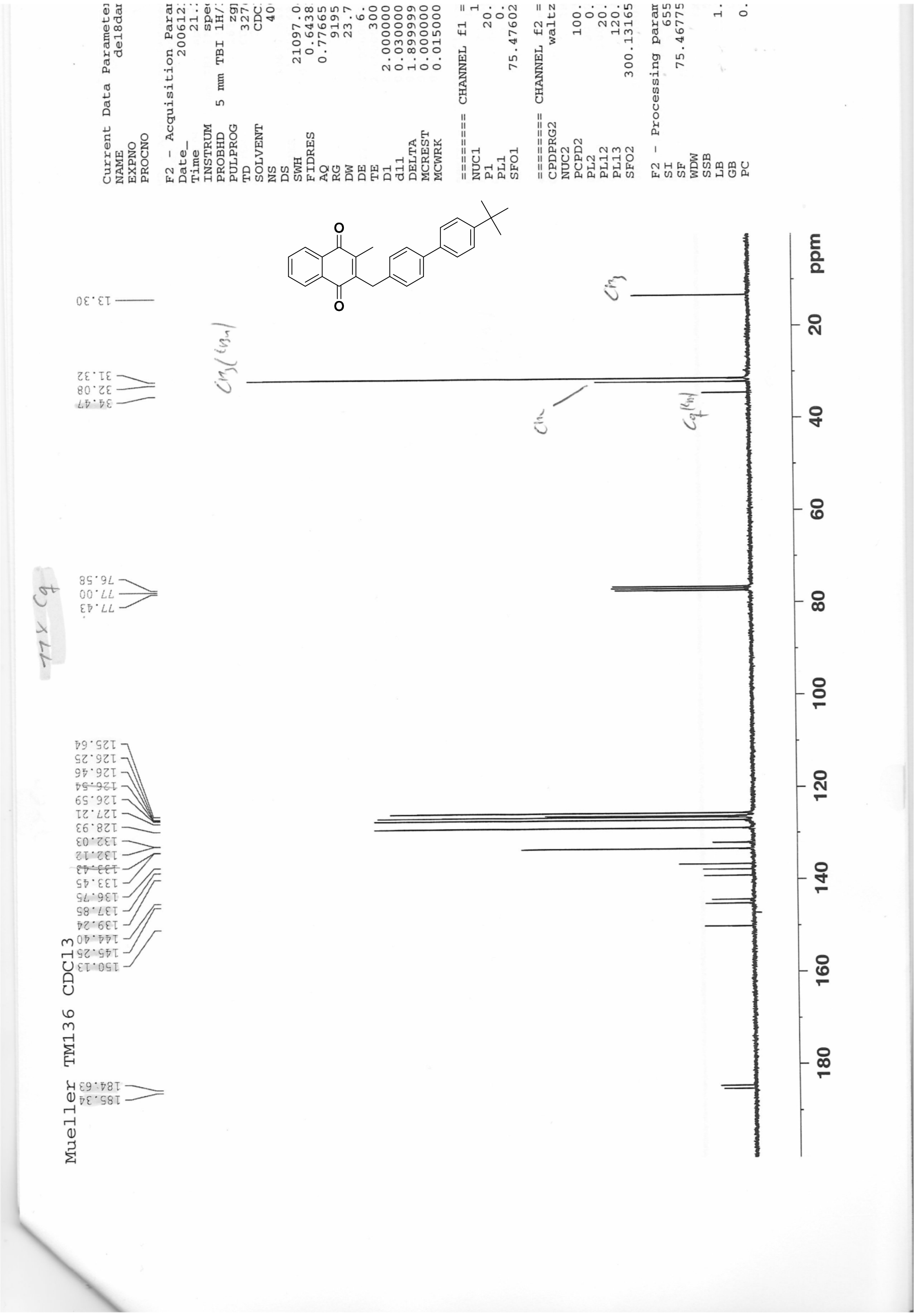

Figure S44. ^{13}C-NMR (75 MHz, $CDCl_3$) spectrum of compound **27**.

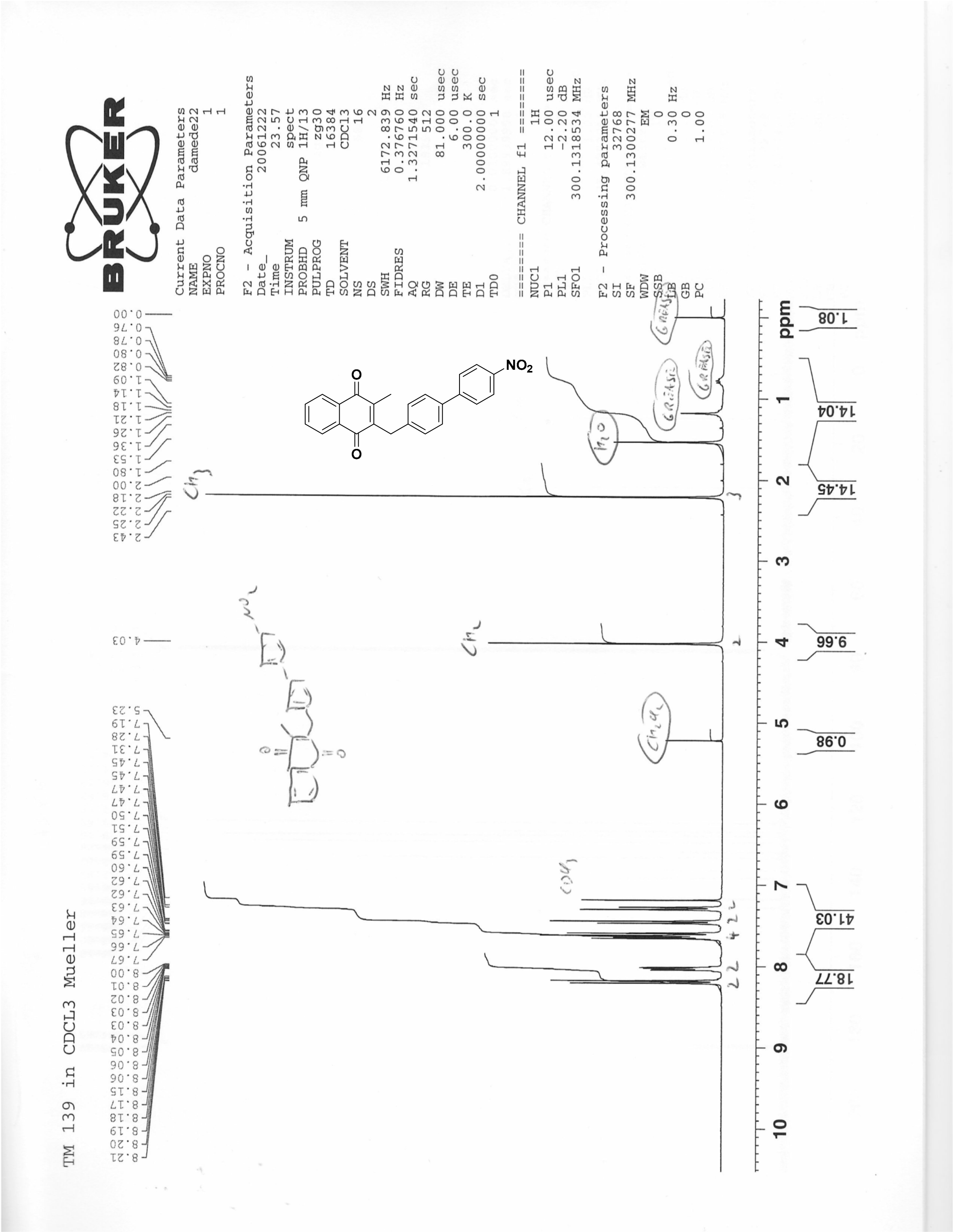

Figure S45. ^{1}H-NMR (300 MHz, $CDCl_3$) spectrum of compound **28**.

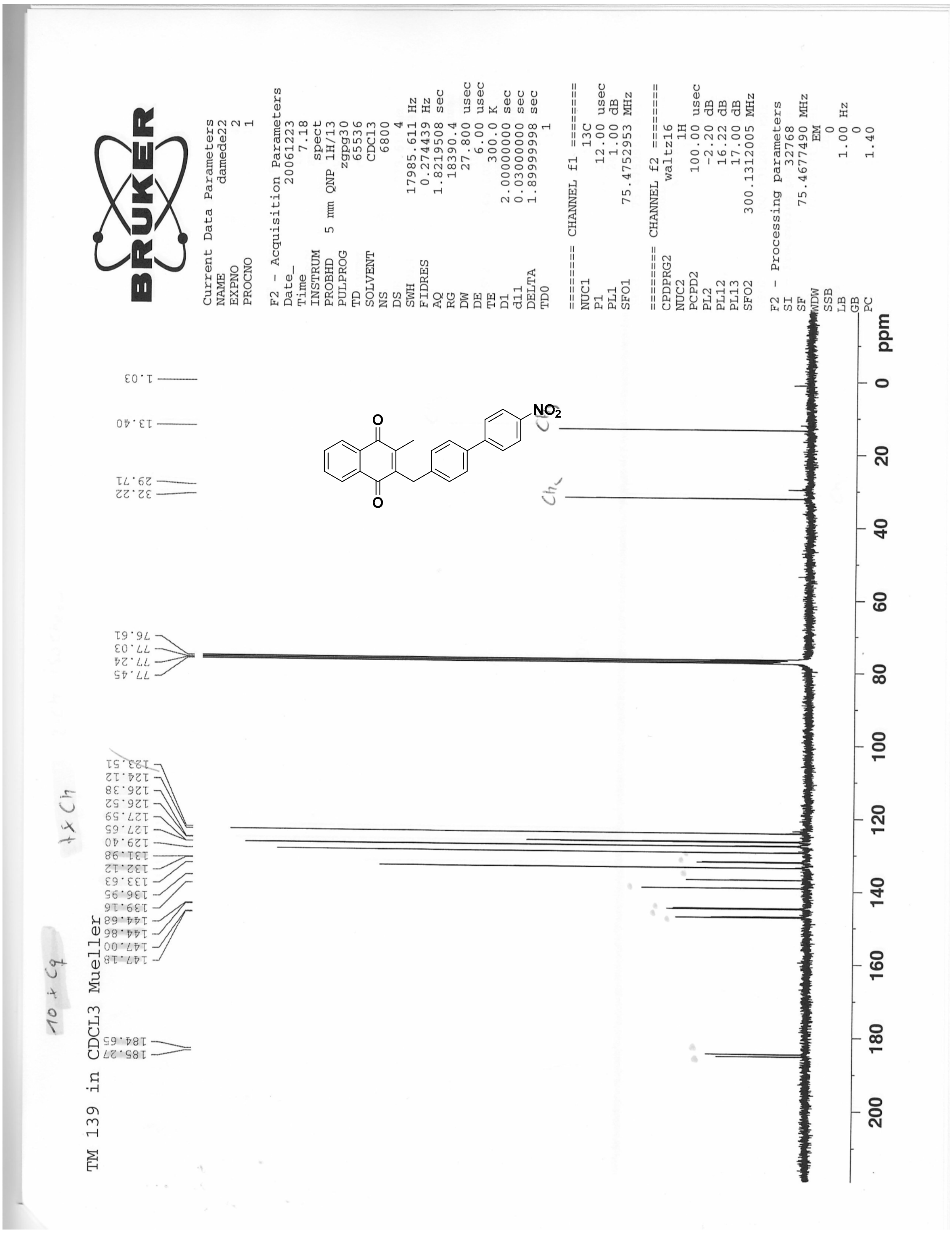

Figure S46. ^{13}C-NMR (75 MHz, $CDCl_3$) spectrum of compound **28**.
